# Supplementary material for: Genome-wide association and HLA region fine-mapping studies identify susceptibility loci for multiple common infections
Source: Nat Commun. 2017 Sep 19;8:599. doi: 10.1038/s41467-017-00257-5 (PMC5605711; doi:10.1038/s41467-017-00257-5)
Supplement: Supplementary file 1 — Supplementary Information [file 41467_2017_257_MOESM1_ESM.pdf]

File name: Supplementary Information
Description: Supplementary Figures, Supplementary Tables, Supplementary Notes and Supplementary References

File name: Supplementary Data 1
Description: GWAS summary statistics for the top 8,000 SNPs for each phenotype.

File name: Supplementary Data 2
Description: Association test statistics for the HLA alleles and HLA amino acid with $P < 0.05$ for each phenotype.

File name: Peer Review File
Description:

Supplementary Figure 1: Manhattan plots and Q-Q plots of GWAS results

Manhattan plot: The gray line corresponds to $p=5\text{x}10^{-8}$, and results above this threshold are shown in red. Gene labels are provided for cross-referencing with other results and are not intended to suggest that we have established a causal basis for the observed association. The test statistics in the Manhattan plots have already been adjusted using genomic control.

Q-Q plot: The Q-Q plot depicts observed versus expected quantiles for the GWAS *p* values, where the expected distribution of *p* values is uniform under the null hypothesis, plotted on a log scale. A solid red line is shown with a slope of 1, and dashed red lines represent a 95% confidence envelope under the assumption that the test results are independent. A "good" Q-Q plot follows the null distribution for larger *p* values ($p>0.01$) then diverges from the null distribution for small *p* values. The test statistics in the Q-Q plots have already been adjusted using genomic control.

Chickenpox:

The results in the plots have been adjusted for a genomic control inflation factor

$\lambda=1.052$.

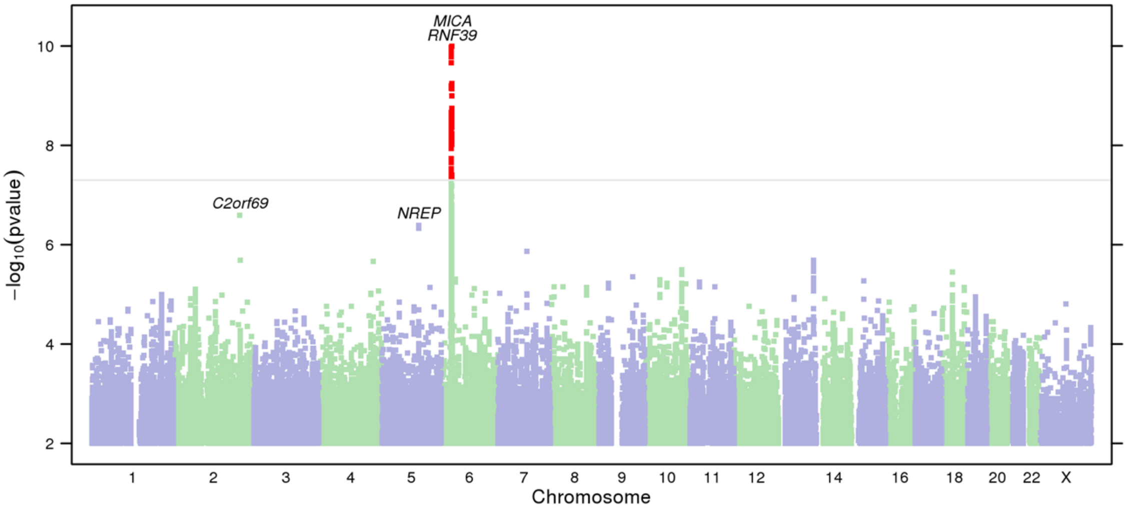

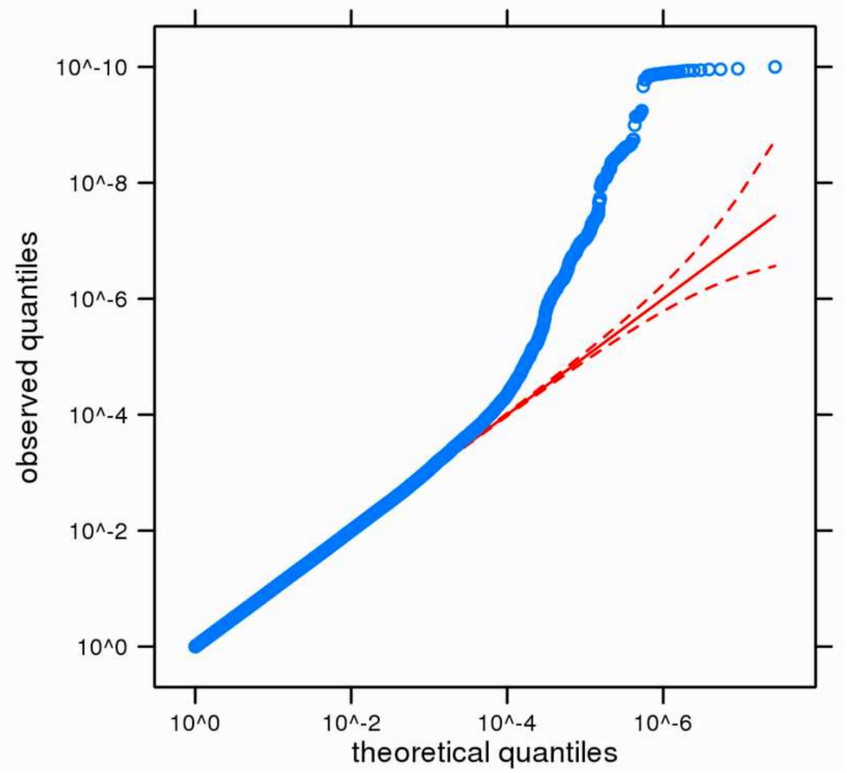

Shingles

The results in the plots have been adjusted for a genomic control inflation factor λ=1.025.

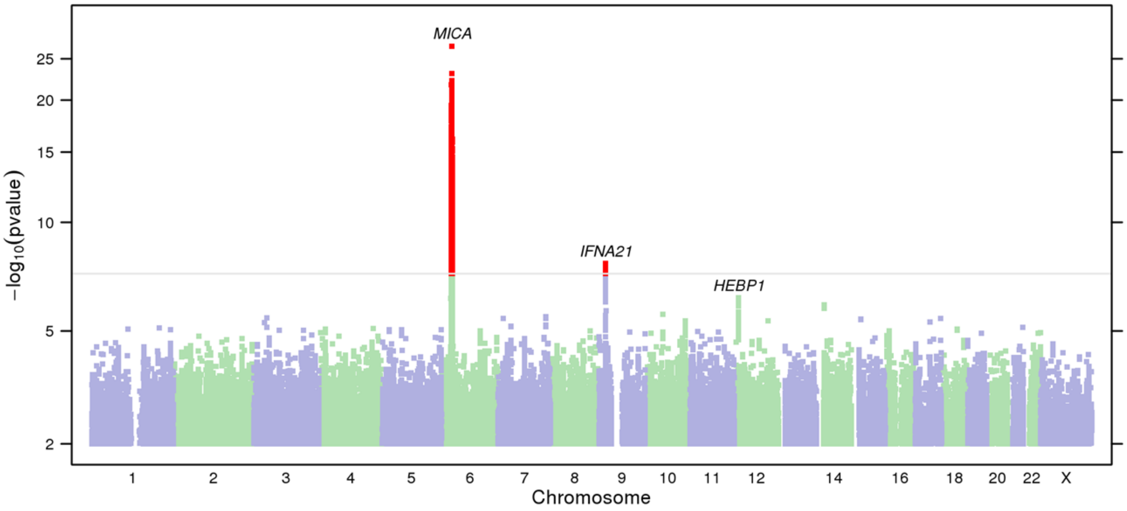

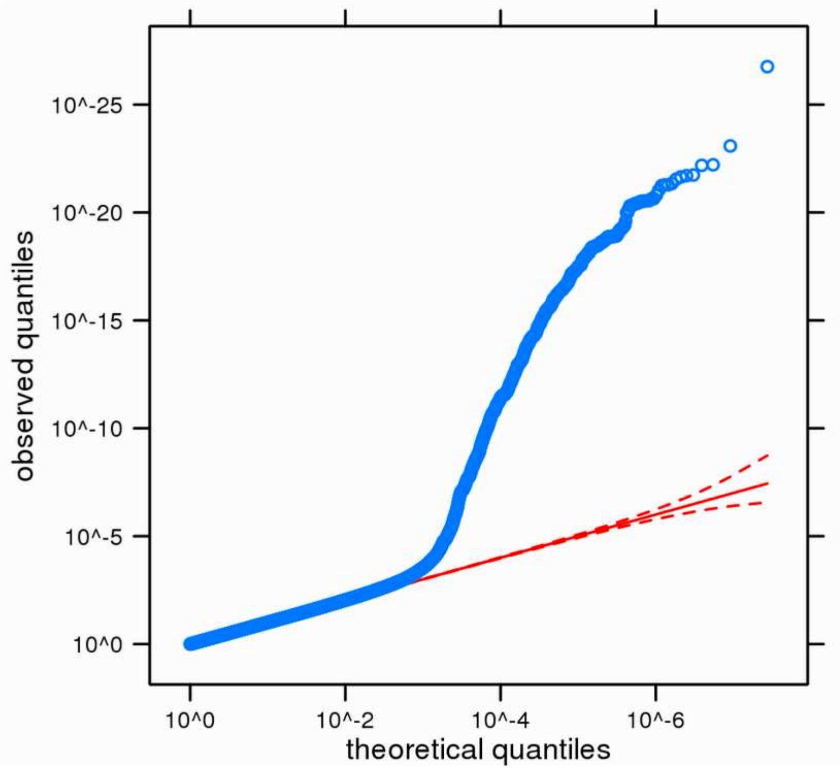

Cold sores:

The results in the plots have been adjusted for a genomic control inflation factor λ=1.015.

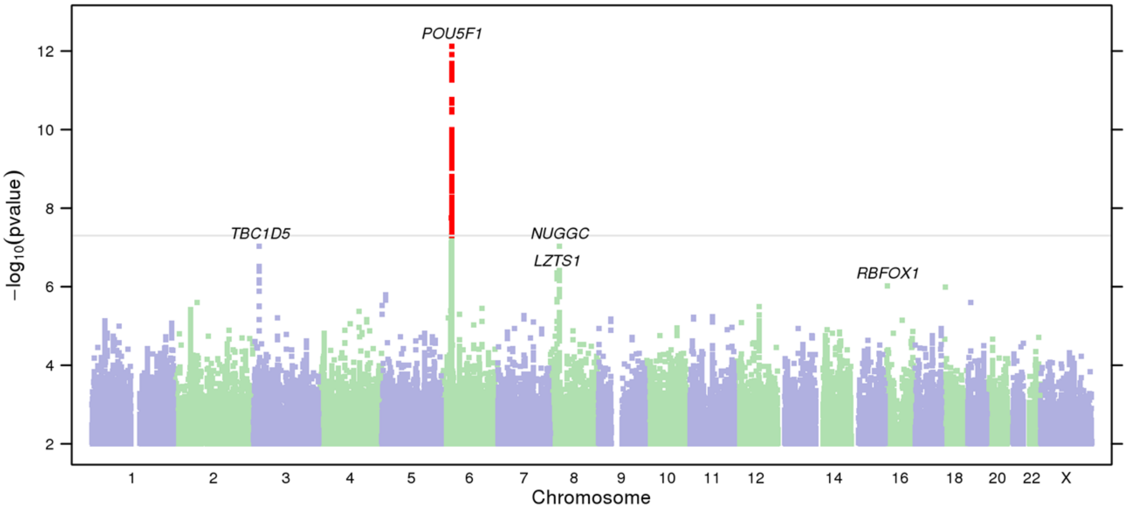

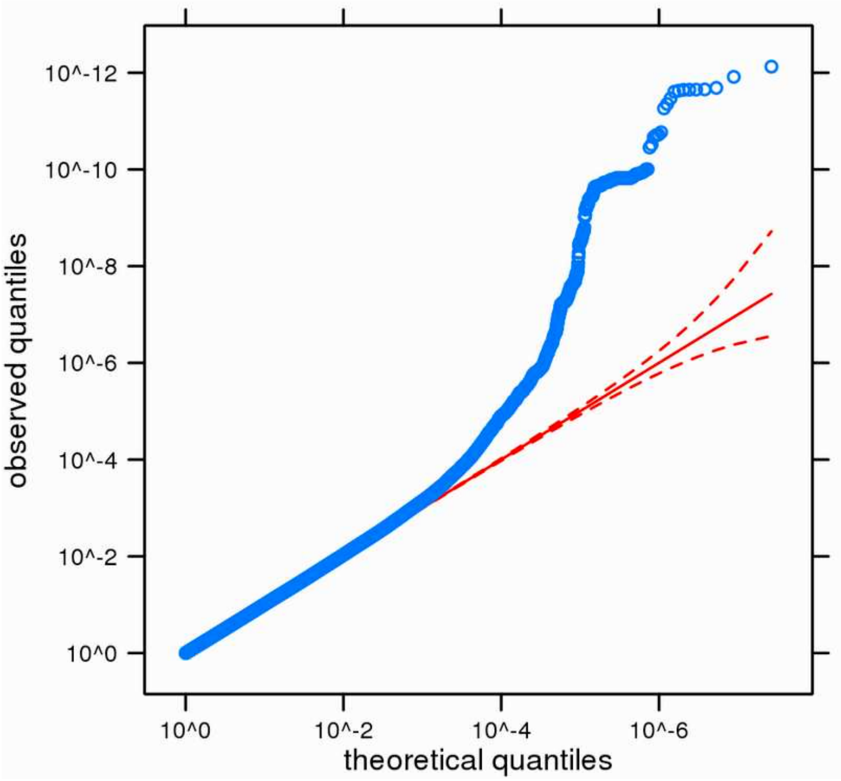

Mononucleosis:

The results in the plots have been adjusted for a genomic control inflation factor λ=1.019.

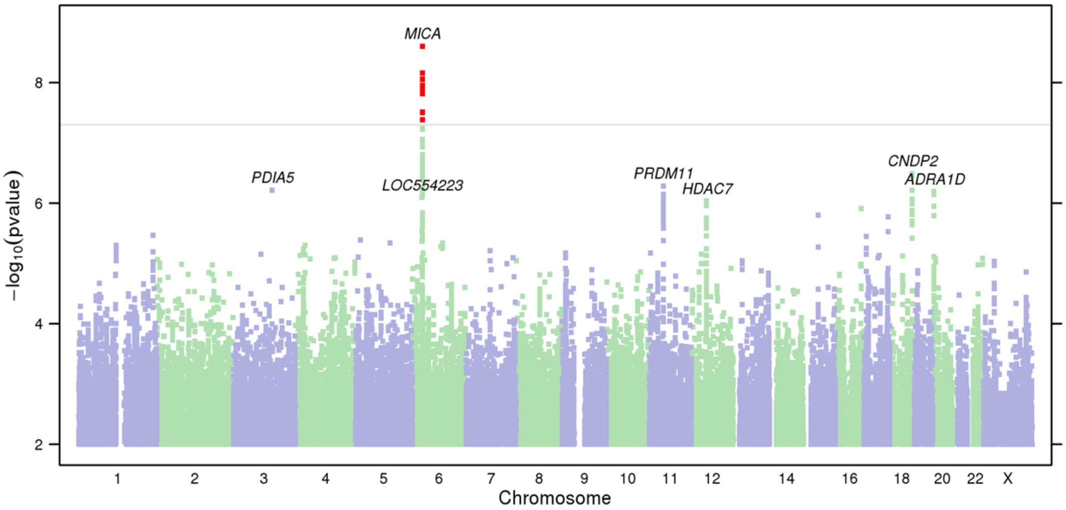

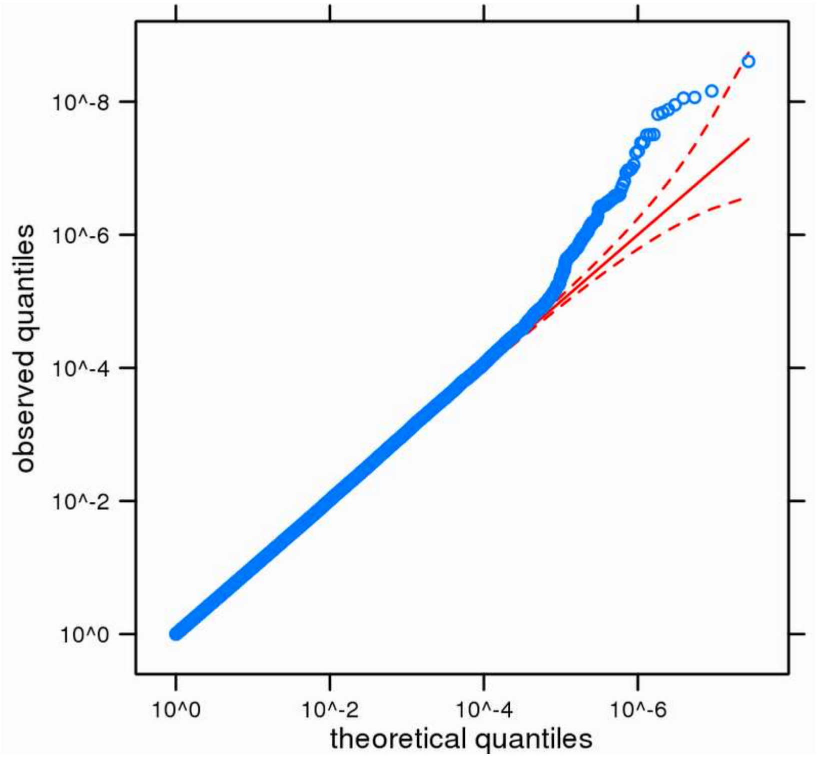

Mumps:

The results in the plots have been adjusted for a genomic control inflation factor λ=1.024.

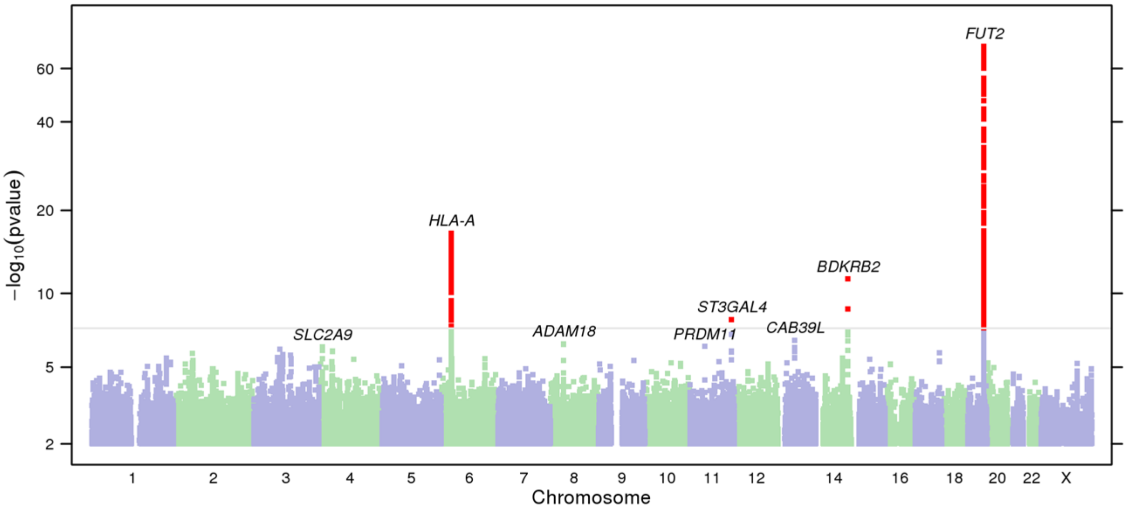

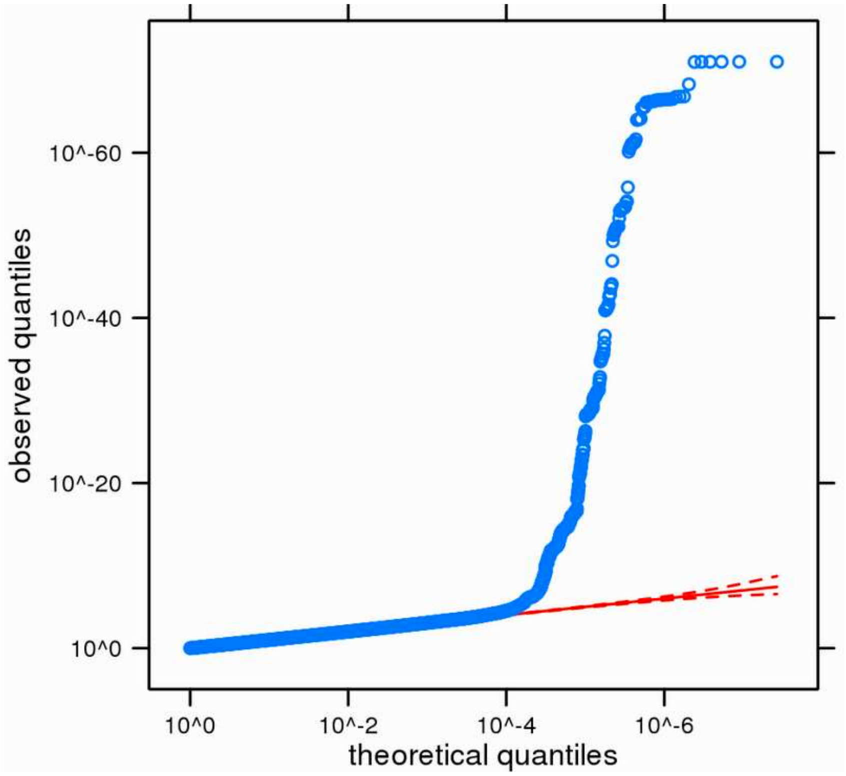

Hepatitis A:

The results in the plots have been adjusted for a genomic control inflation factor λ=1.010.

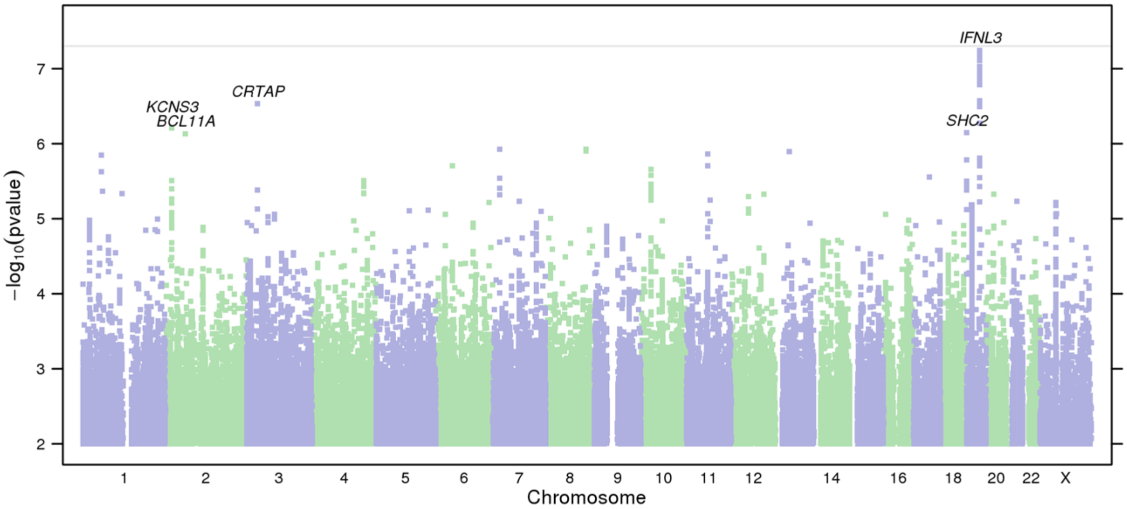

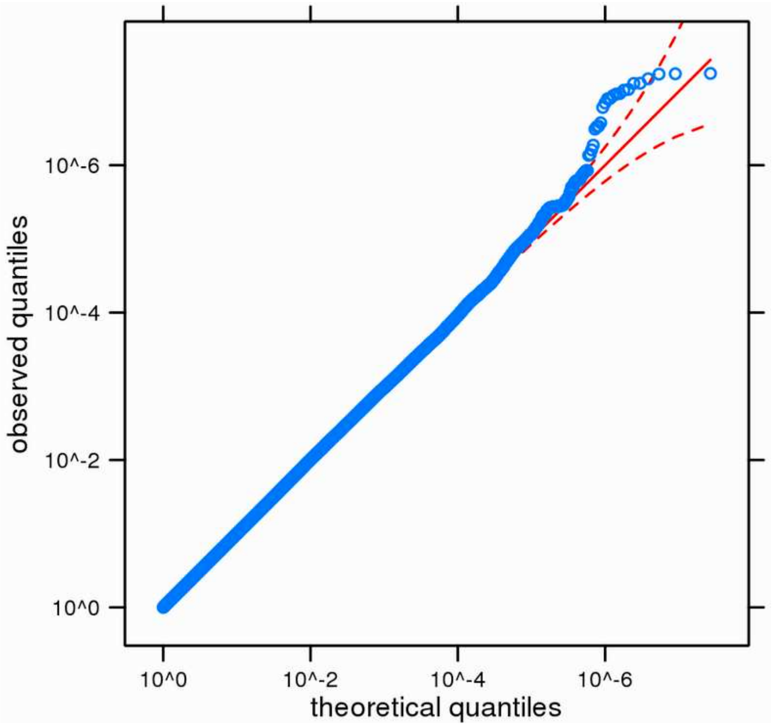

Hepatitis B:

The results in the plots have been adjusted for a genomic control inflation factor λ=1.017.

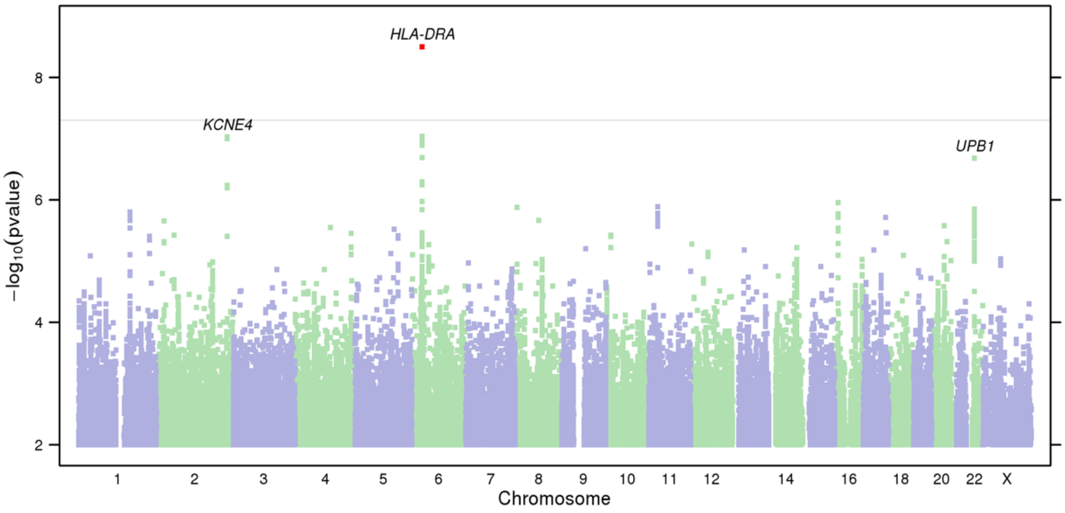

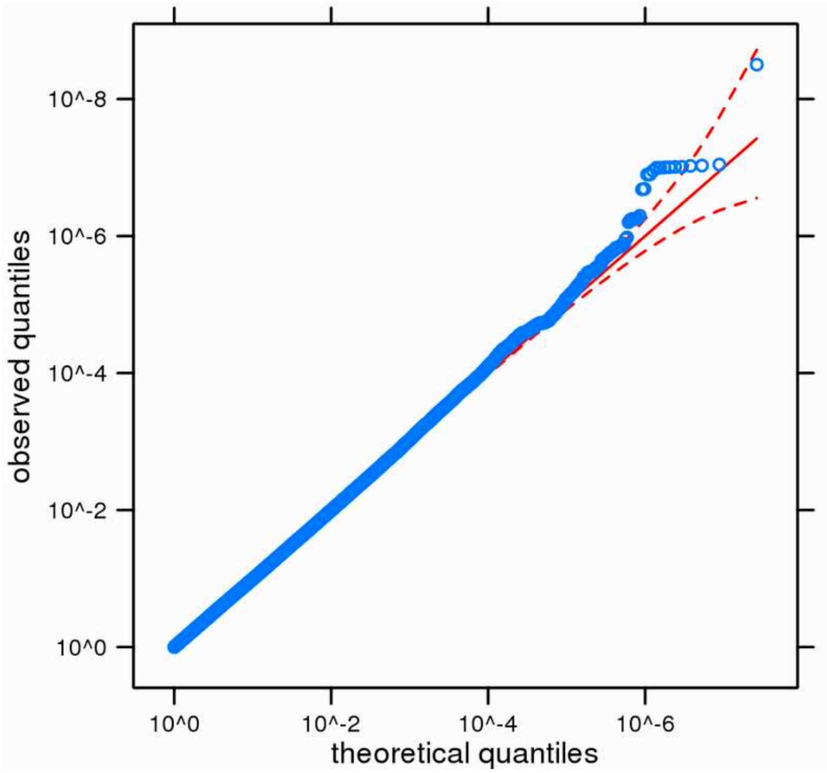

Plantar warts:

The results in the plots have been adjusted for a genomic control inflation factor λ=1.057.

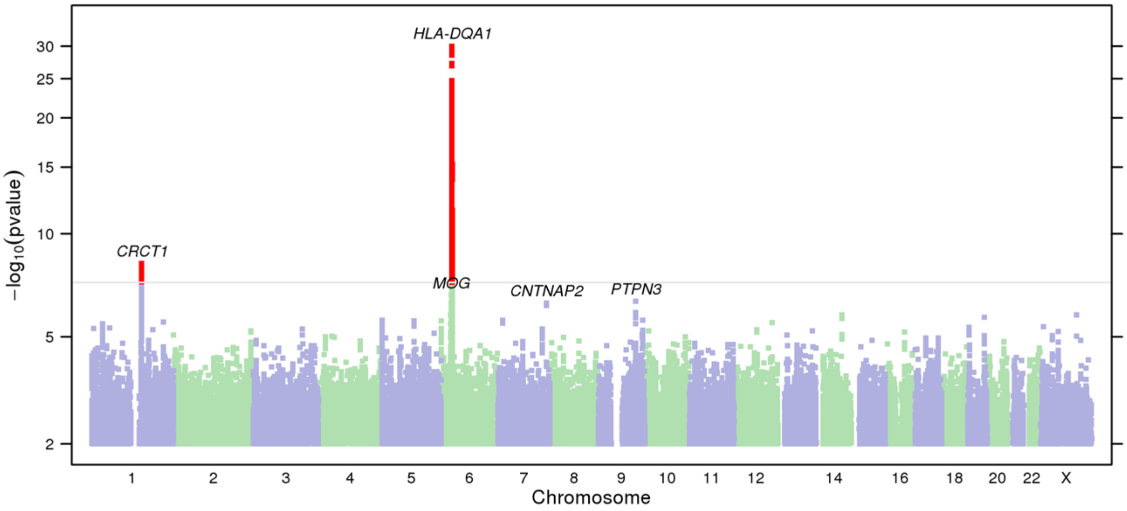

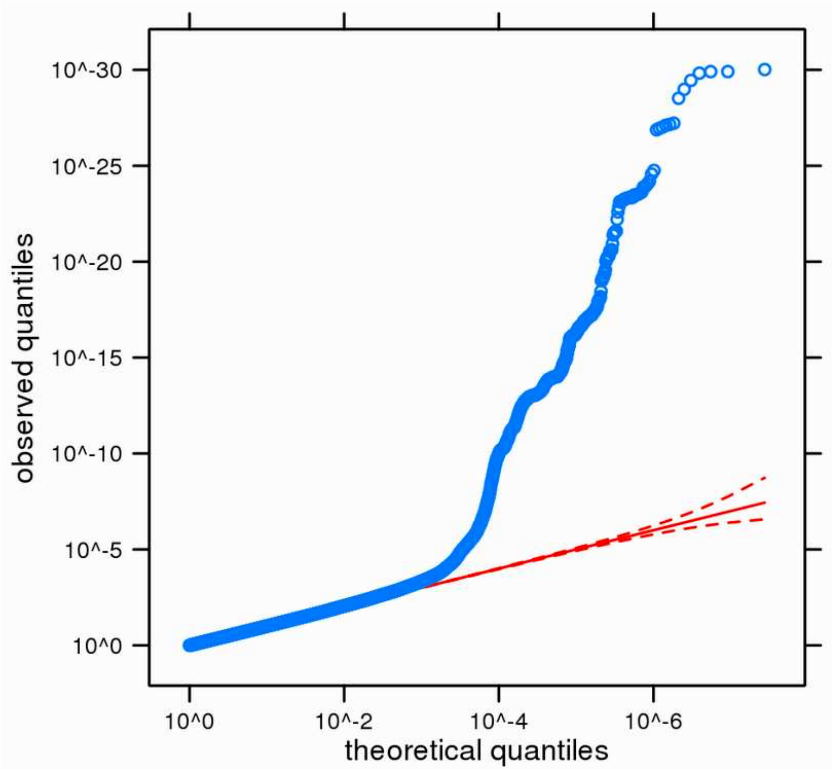

10^-30
10^-25
10^-20
10^-15
10^-10
10^-5
10^0
observed quantiles
10^0
10^-2
10^-4
10^-6
theoretical quantiles

Positive TB test:

The results in the plots have been adjusted for a genomic control inflation factor λ=1.020.

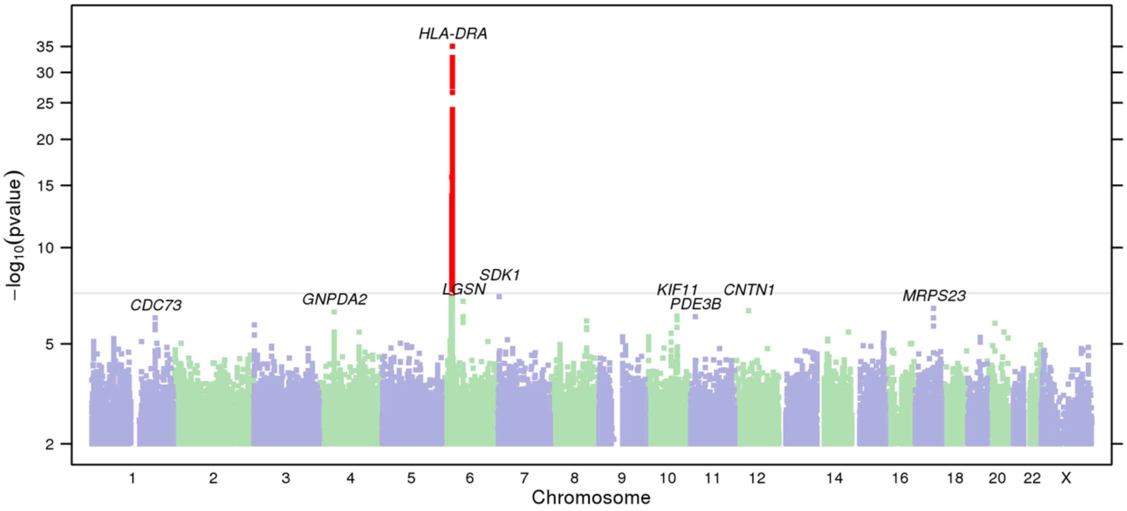

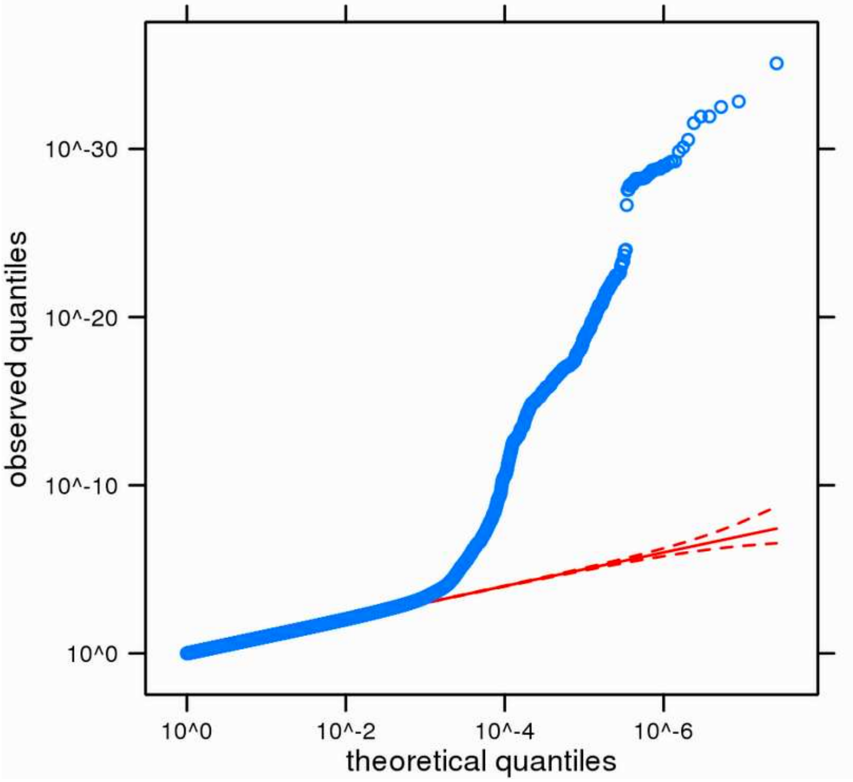

Strep Throat:

The results in the plots have been adjusted for a genomic control inflation factor λ=1.047.

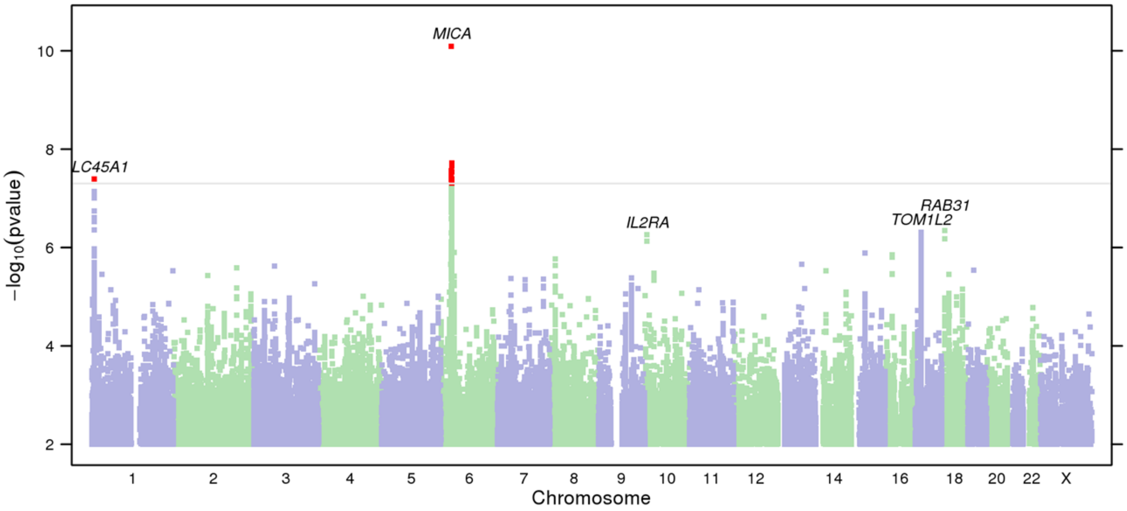

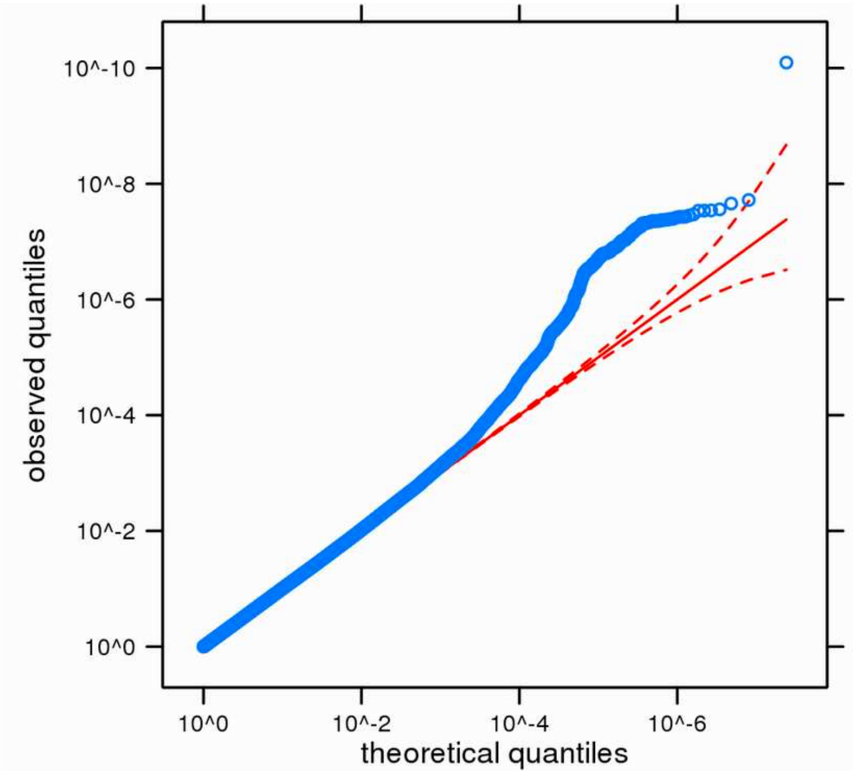

Scarlet fever:

The results in the plots have been adjusted for a genomic control inflation factor λ=1.020.

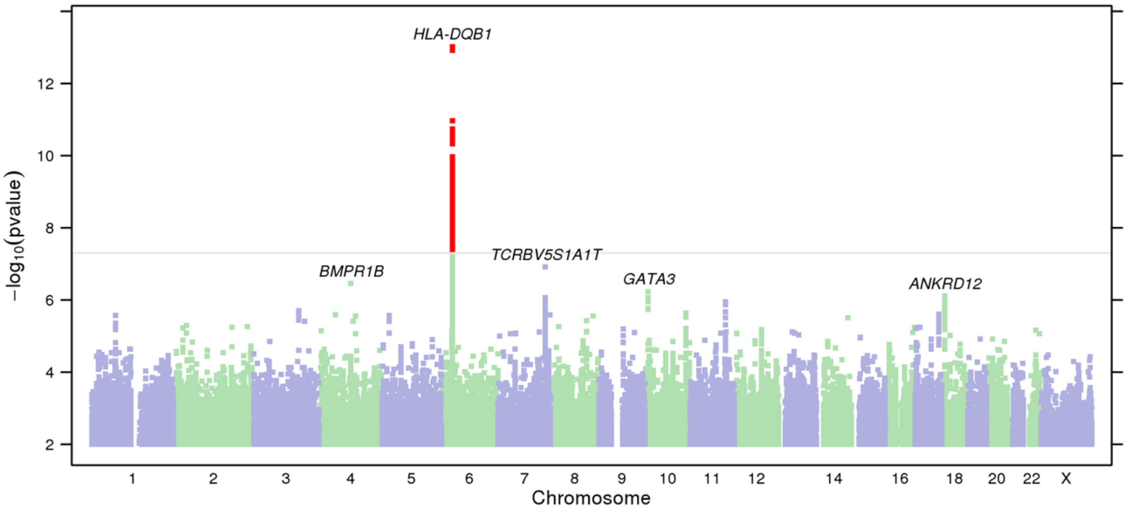

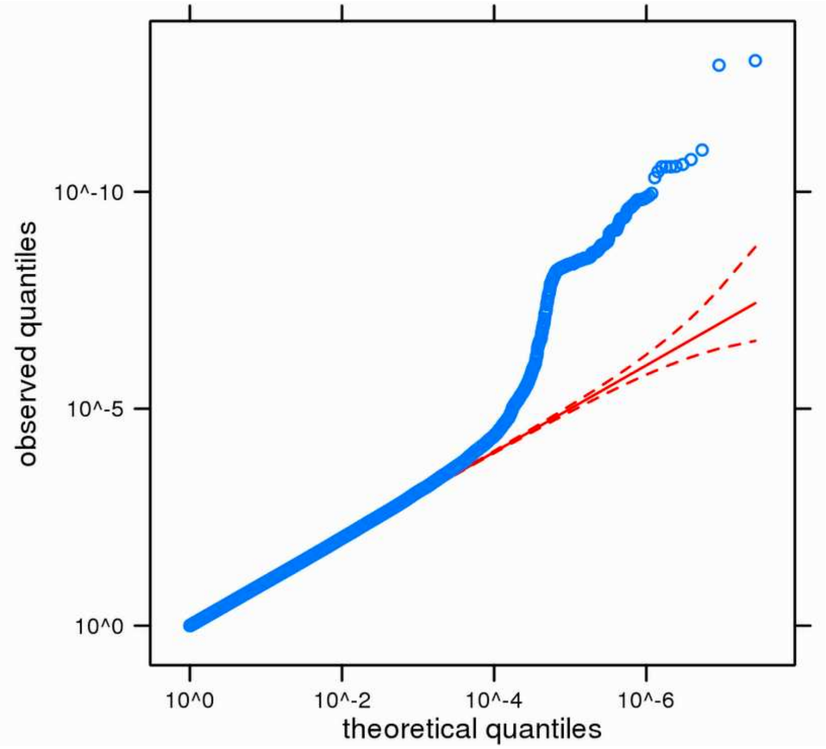

Pneumonia:

The results in the plots have been adjusted for a genomic control inflation factor λ=1.067.

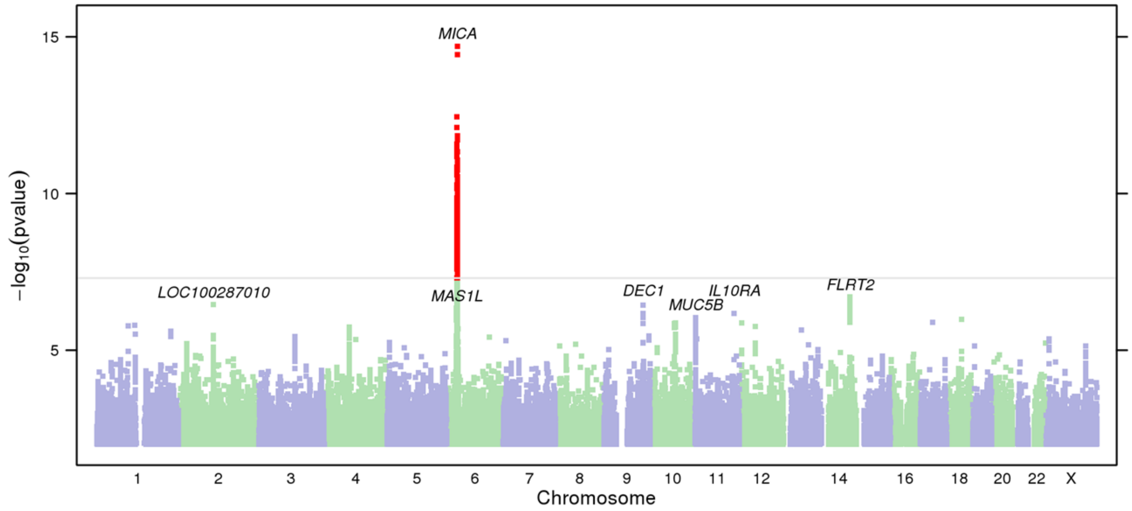

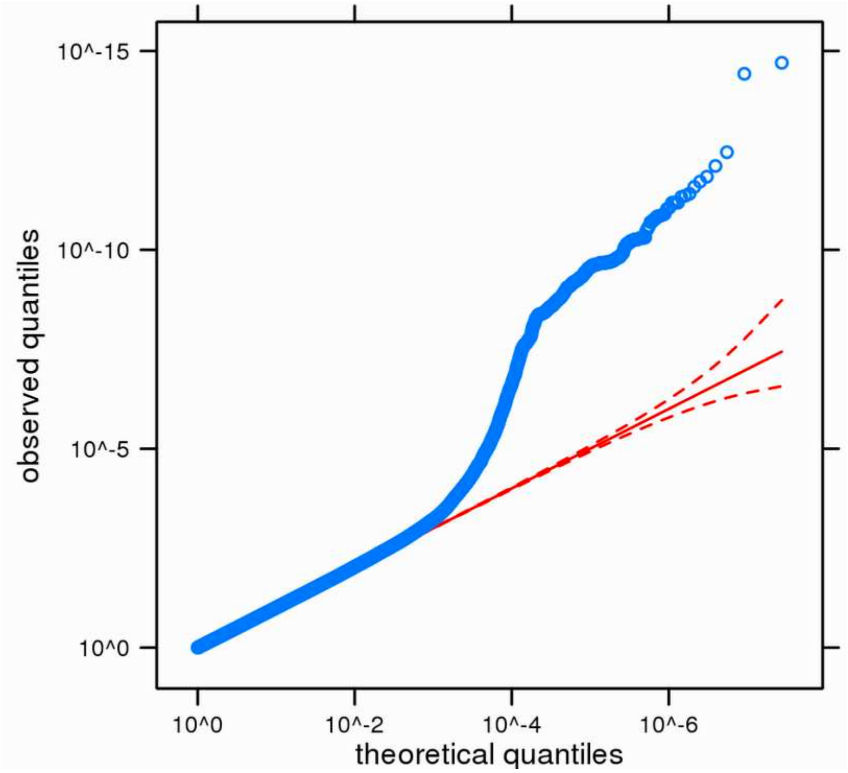

Bacterial meningitis:

The results in the plots have been adjusted for a genomic control inflation factor λ=1.006.

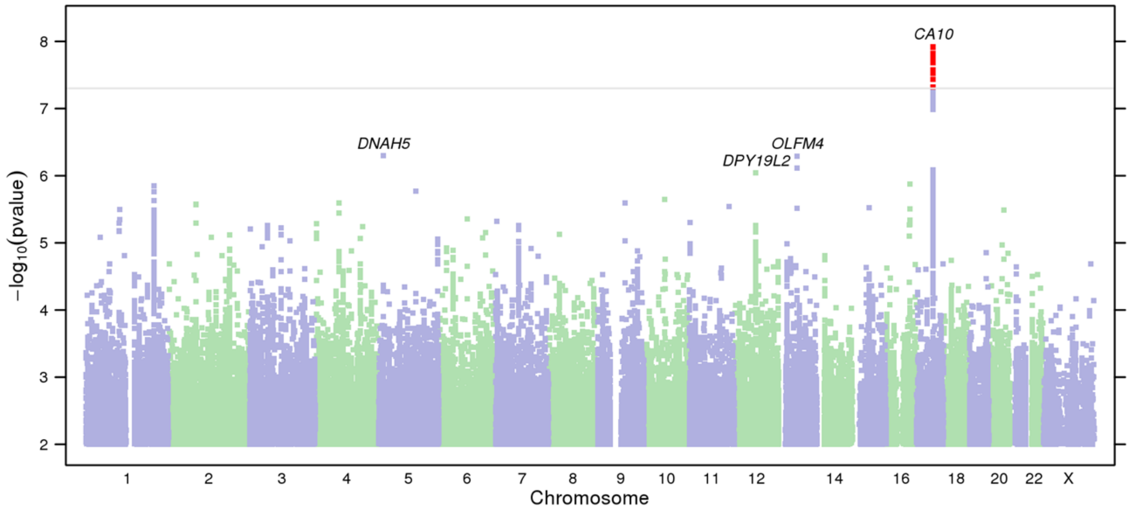

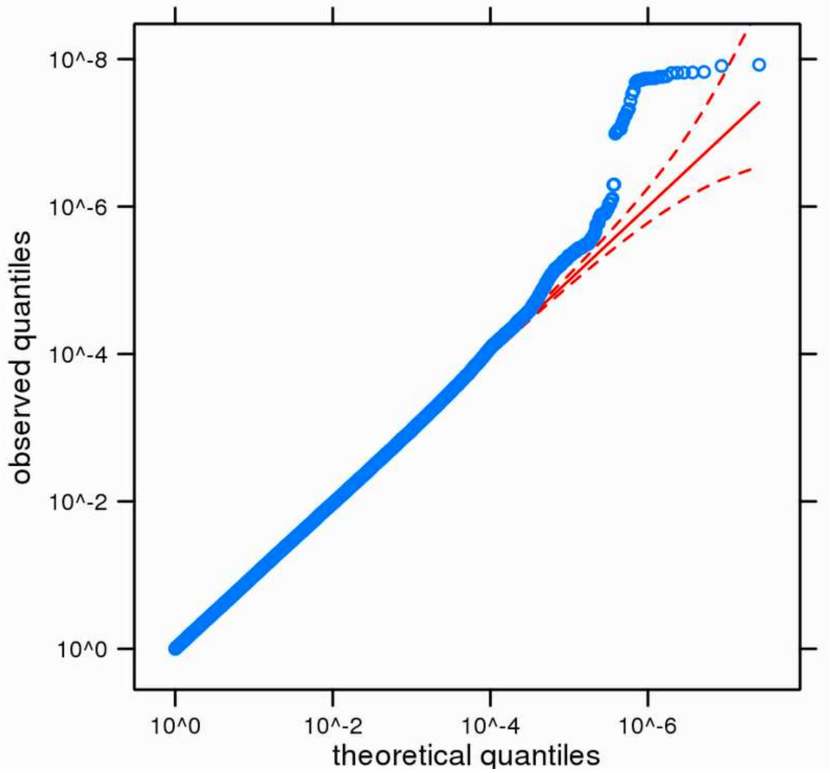

Yeast infections:

The results in the plots have been adjusted for a genomic control inflation factor λ=1.057.

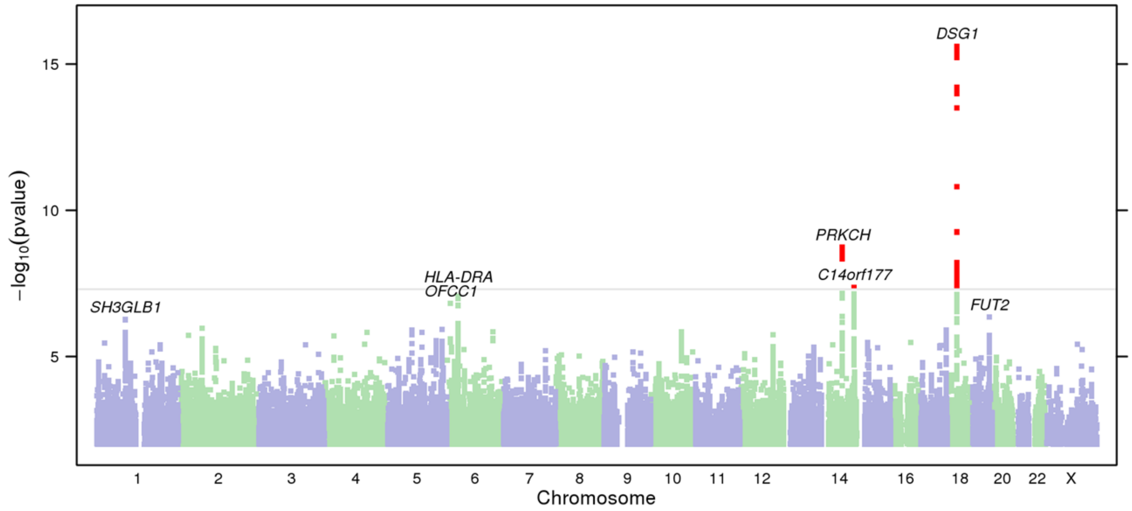

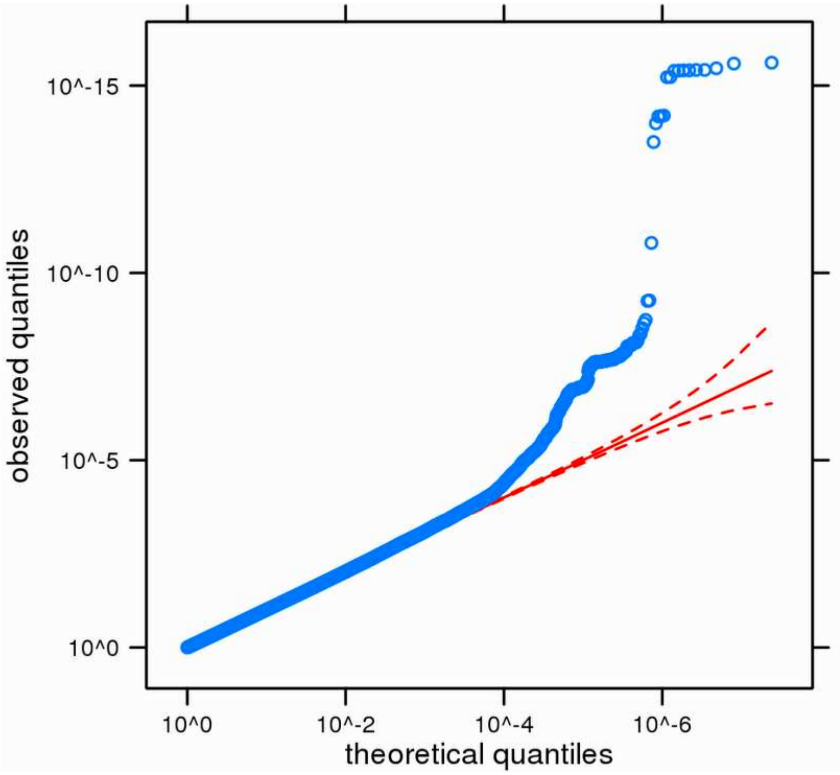

Urinary tract infection frequency:

The results in the plots have been adjusted for a genomic control inflation factor λ=1.047.

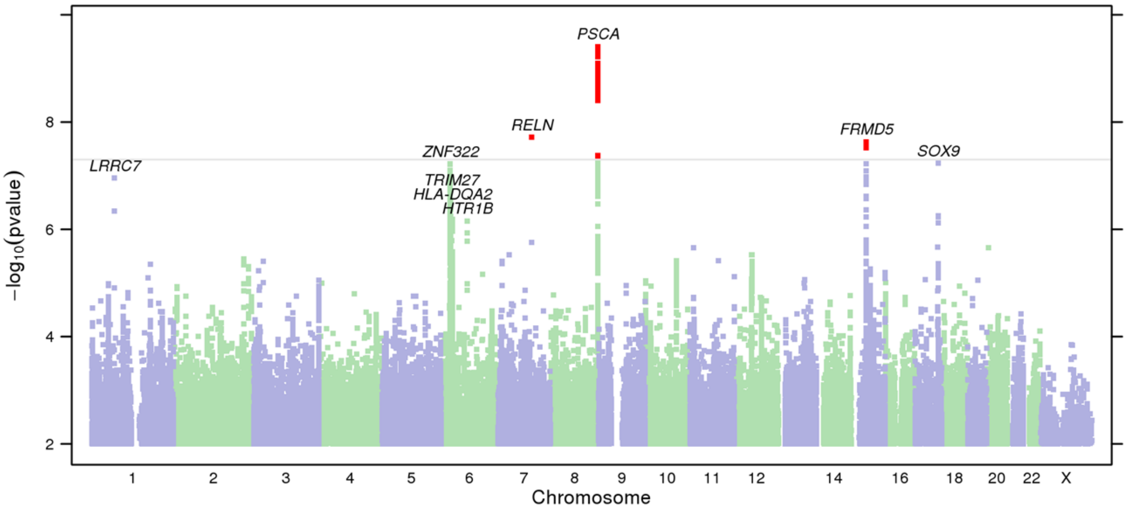

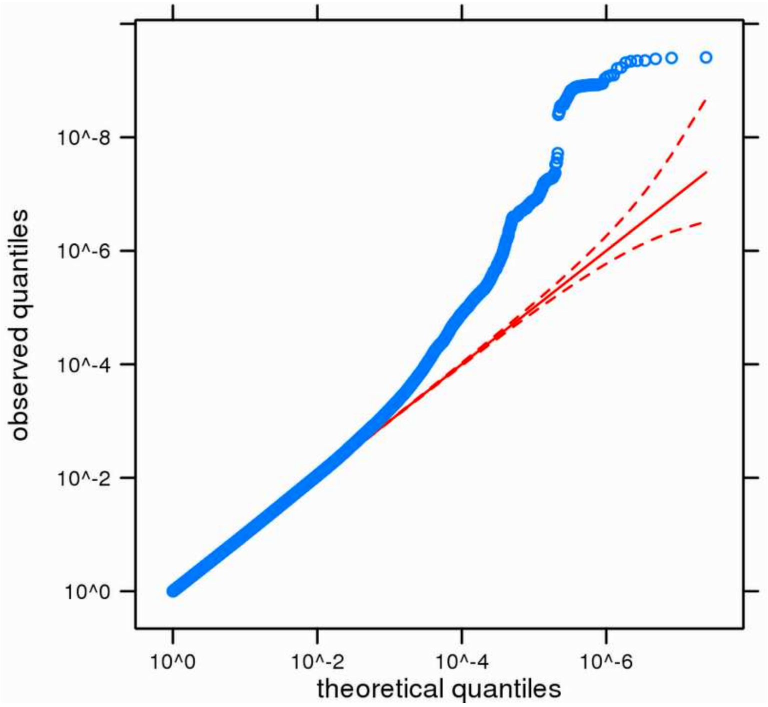

Tonsillectomy:

The results in the plots have been adjusted for a genomic control inflation factor λ=1.103.

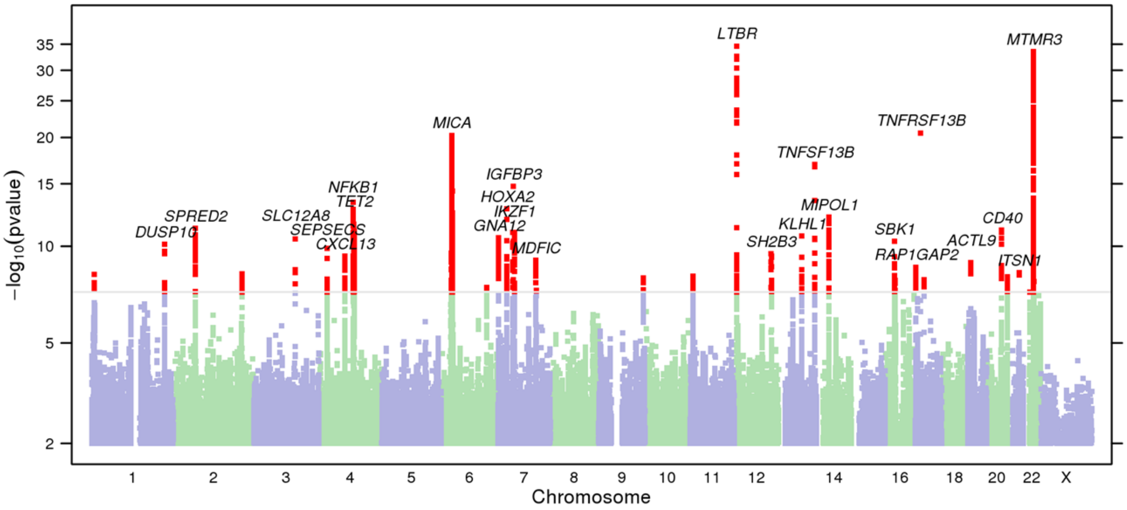
LTBR
MTMR3
TNFRSF13B
MICA
TNFSF13B
IGFBP3
NFKB1
TET2
HOXA2
IKZF1
MIPOL1
SPRED2
SLC12A8
GNA12
KLHL1
CD40
DUSP1G
SEPSECS
SBK1
CXCL13
SH2B3
ACTL9
MDFIC
RAP1GAP2
ITSN1
$-\log_{10}$(pvalue)
Chromosome

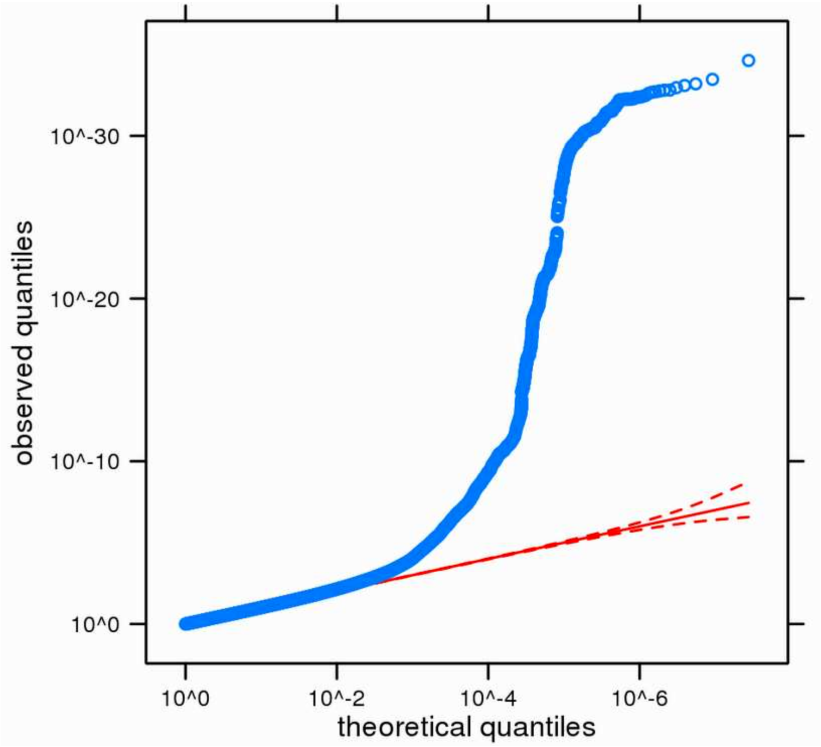
10^-30
10^-20
10^-10
10^0
observed quantiles
10^0
10^-2
10^-4
10^-6
theoretical quantiles

Childhood ear infection:
The results in the plots have been adjusted for a genomic control inflation factor λ=1.087.

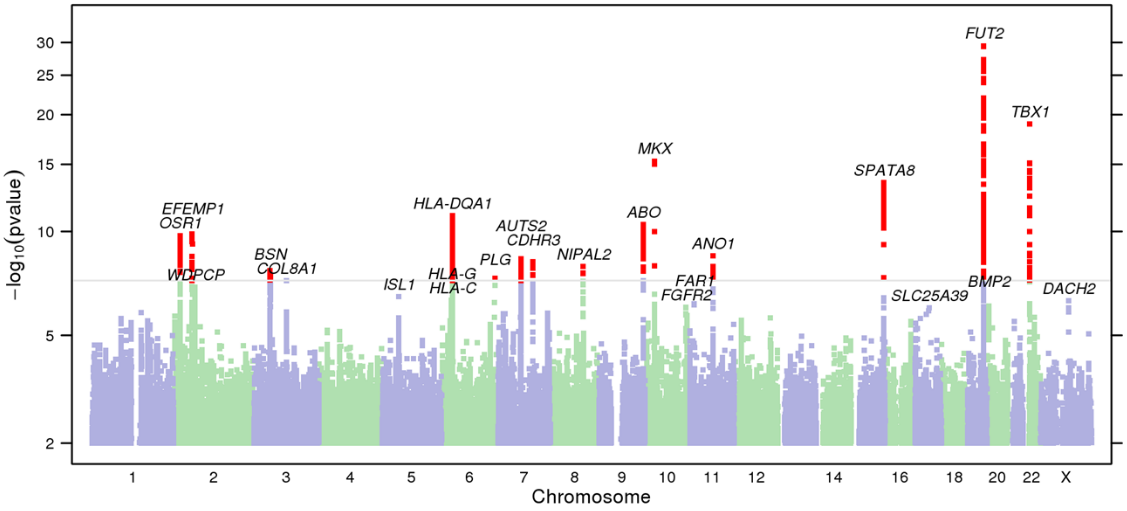

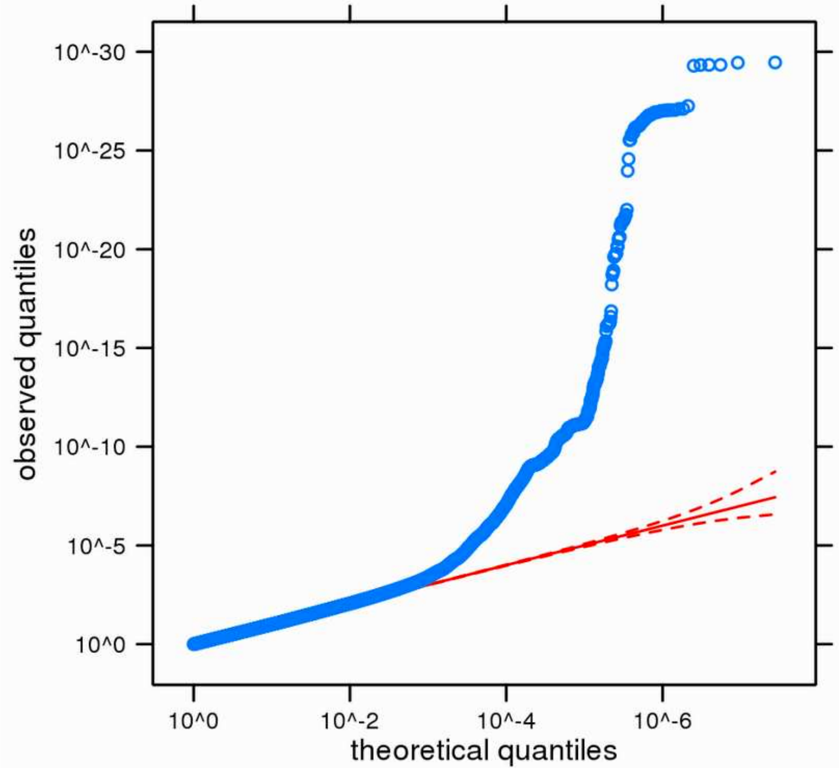

Myringotomy:
The results in the plots have been adjusted for a genomic control inflation factor λ=1.023.

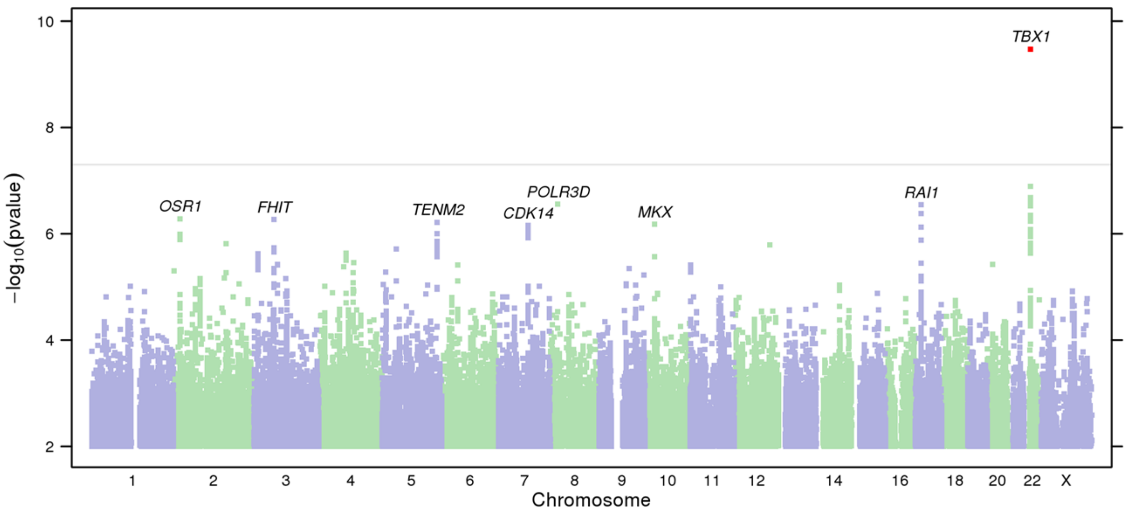
TBX1
OSR1
FHIT
TENM2
POLR3D
CDK14
MKX
RAI1
−log10(pvalue)
Chromosome
1
2
3
4
5
6
7
8
9
10
11
12
14
16
18
20
22
X

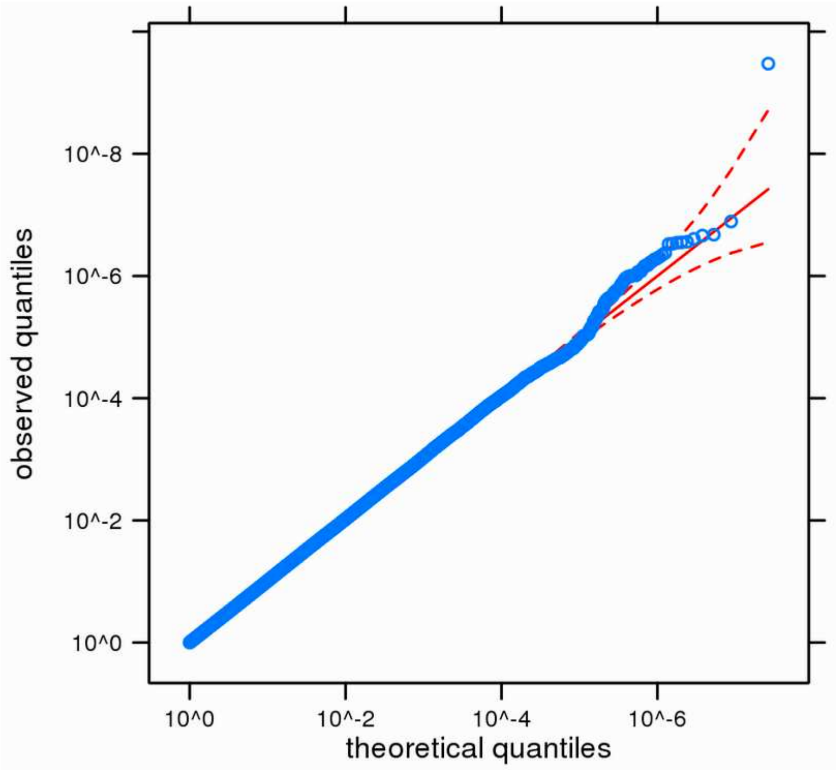
observed quantiles
theoretical quantiles
10^0
10^-2
10^-4
10^-6
10^-8

Supplementary Figure 2: Regional plots of GWAS associations

Symbol colors indicate linkage disequilibrium with the index SNP, which is labeled and colored purple. Open circles indicate imputed variants, filled circles indicate partially genotyped variants, and filled squares indicate fully genotyped variants. Plots were generated using LocusZoom[1]. Results are in NCBI Build 37 coordinates.

Chickenpox:

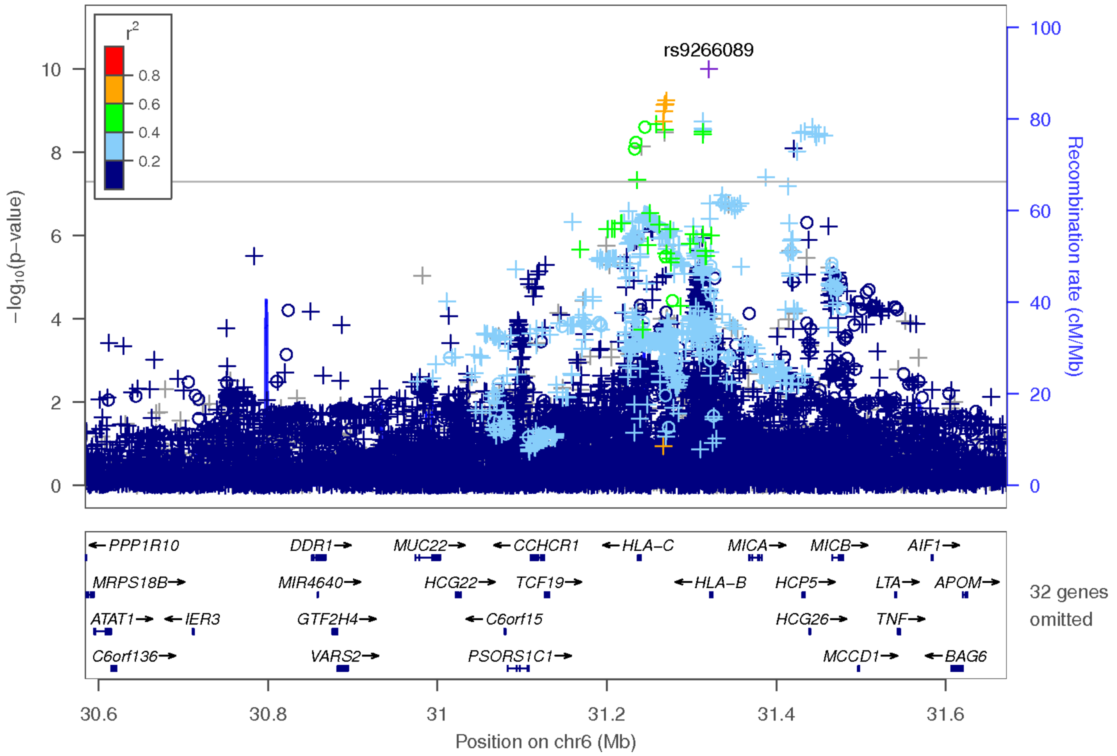

Shingles:

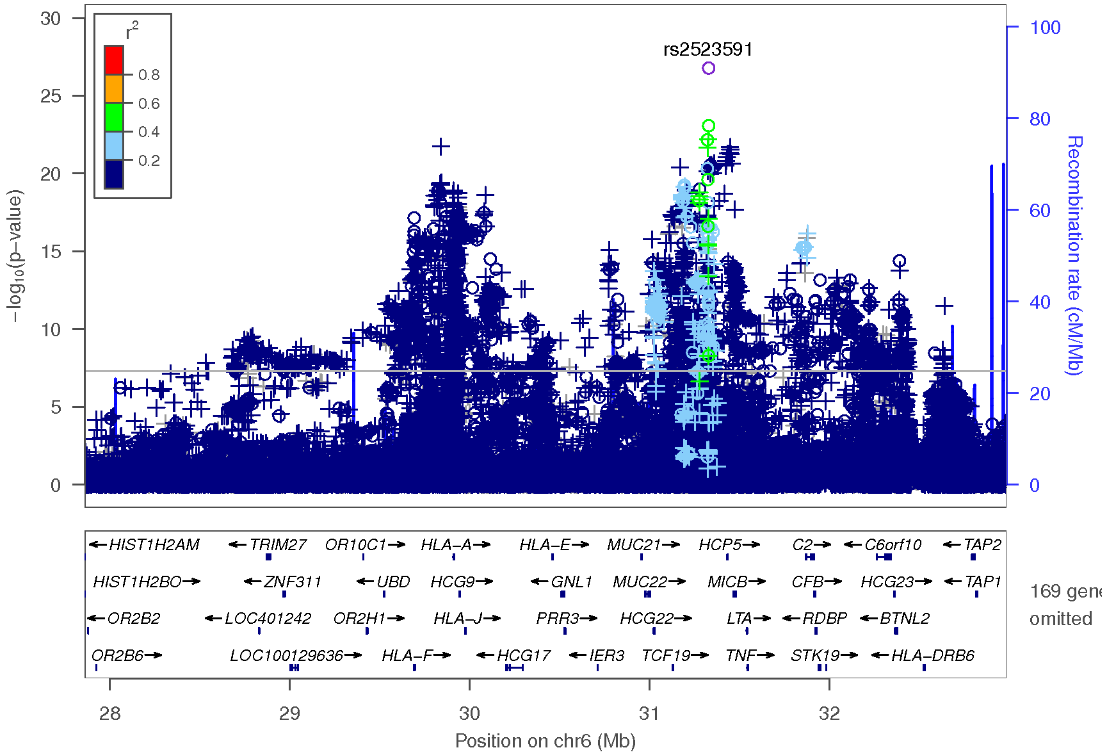
rs2523591
r2
0.8
0.6
0.4
0.2
−log10(p−value)
Recombination rate (cM/Mb)
HIST1H2AM
TRIM27
OR10C1
HLA−A
HLA−E
MUC21
HCP5
C2
C6orf10
TAP2
HIST1H2BO
ZNF311
UBD
HCG9
GNL1
MUC22
MICB
CFB
HCG23
TAP1
OR2B2
LOC401242
OR2H1
HLA−J
PRR3
HCG22
LTA
RDBP
BTNL2
OR2B6
LOC100129636
HLA−F
HCG17
IER3
TCF19
TNF
STK19
HLA−DRB6
169 genes omitted
Position on chr6 (Mb)

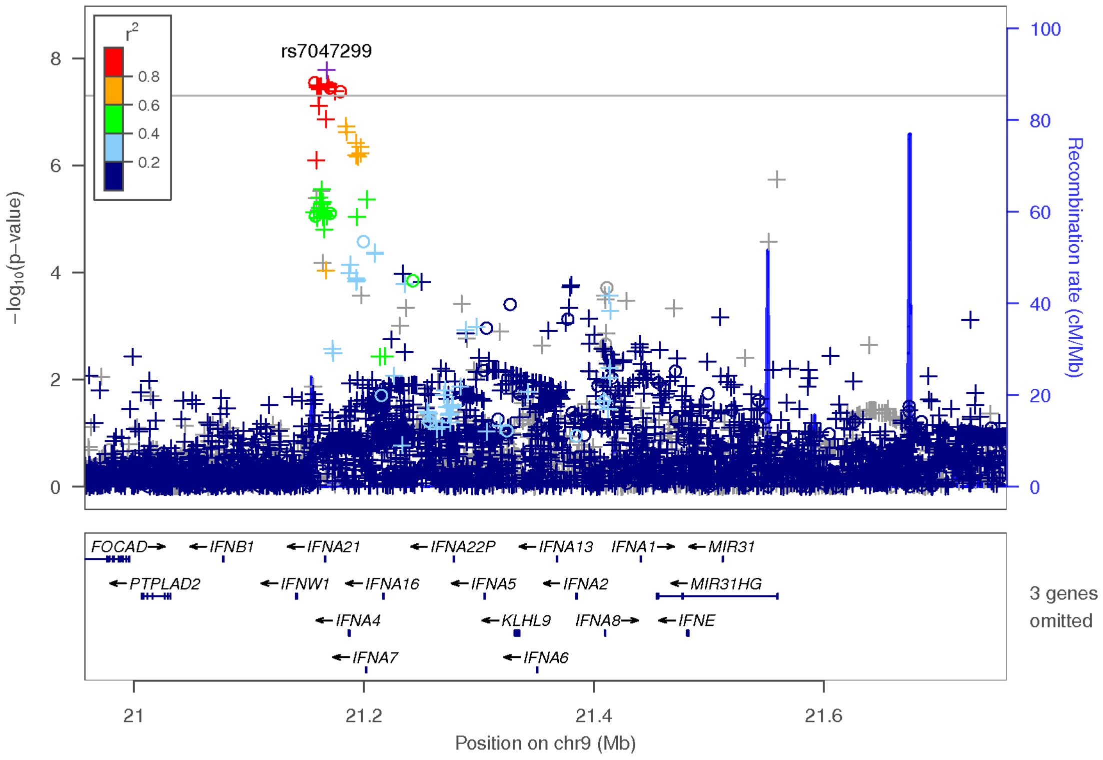
rs7047299
r2
0.8
0.6
0.4
0.2
−log10(p−value)
Recombination rate (cM/Mb)
FOCAD
IFNB1
IFNA21
IFNA22P
IFNA13
IFNA1
MIR31
PTPLAD2
IFNW1
IFNA16
IFNA5
IFNA2
MIR31HG
IFNA4
KLHL9
IFNA8
IFNE
IFNA7
IFNA6
3 genes omitted
Position on chr9 (Mb)

Cold sores:

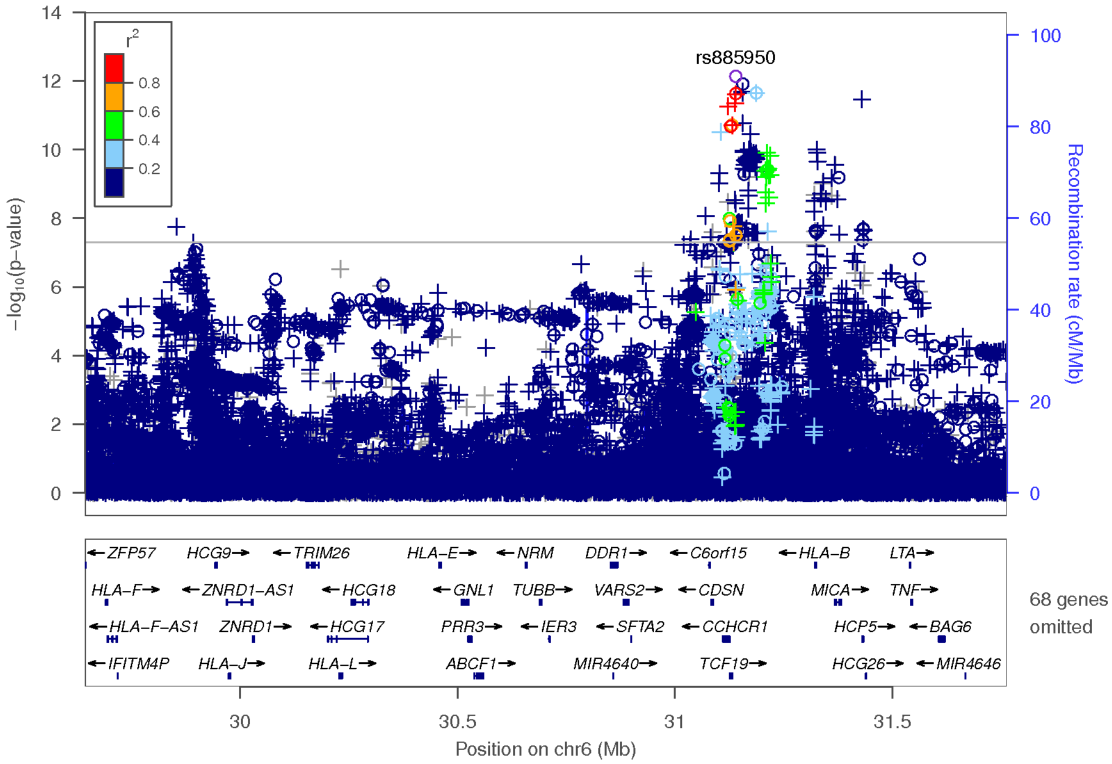
rs885950
r^2
0.8
0.6
0.4
0.2
−log10(p−value)
Recombination rate (cM/Mb)
ZFP57
HCG9
TRIM26
HLA−E
NRM
DDR1
C6orf15
HLA−B
LTA
HLA−F
ZNRD1−AS1
HCG18
GNL1
TUBB
VARS2
CDSN
MICA
TNF
HLA−F−AS1
ZNRD1
HCG17
PRR3
IER3
SFTA2
CCHCR1
HCP5
BAG6
IFITM4P
HLA−J
HLA−L
ABCF1
MIR4640
TCF19
HCG26
MIR4646
68 genes omitted
Position on chr6 (Mb)

Mononucleosis:

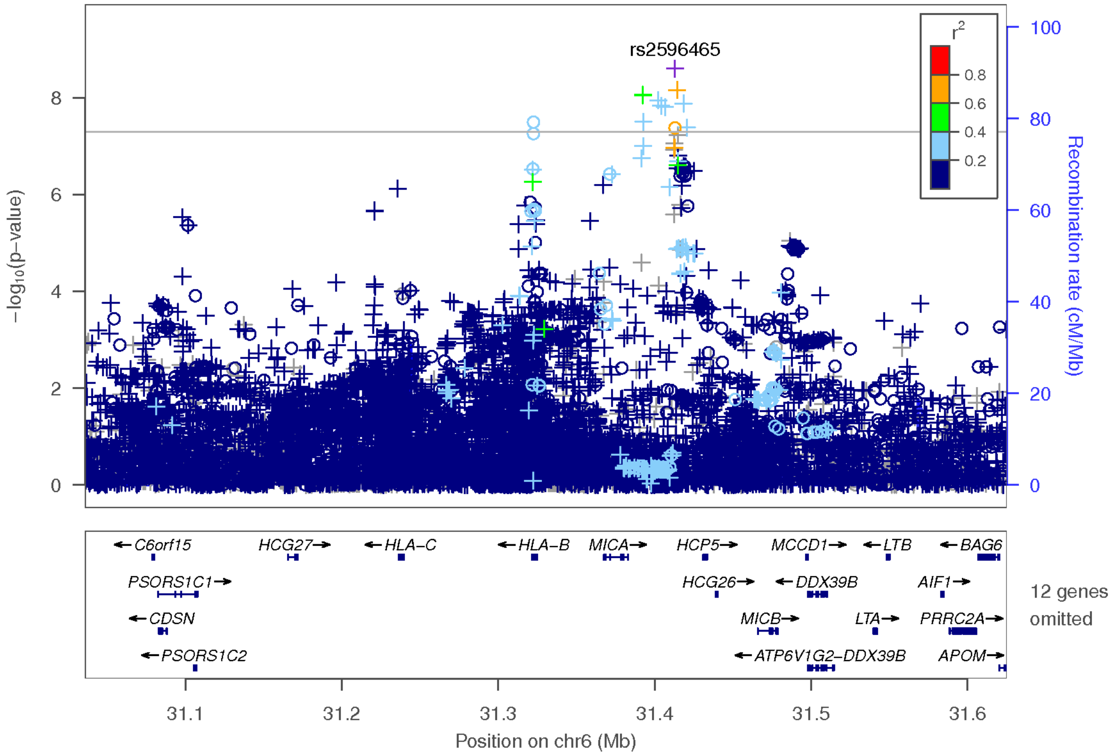

Mumps:

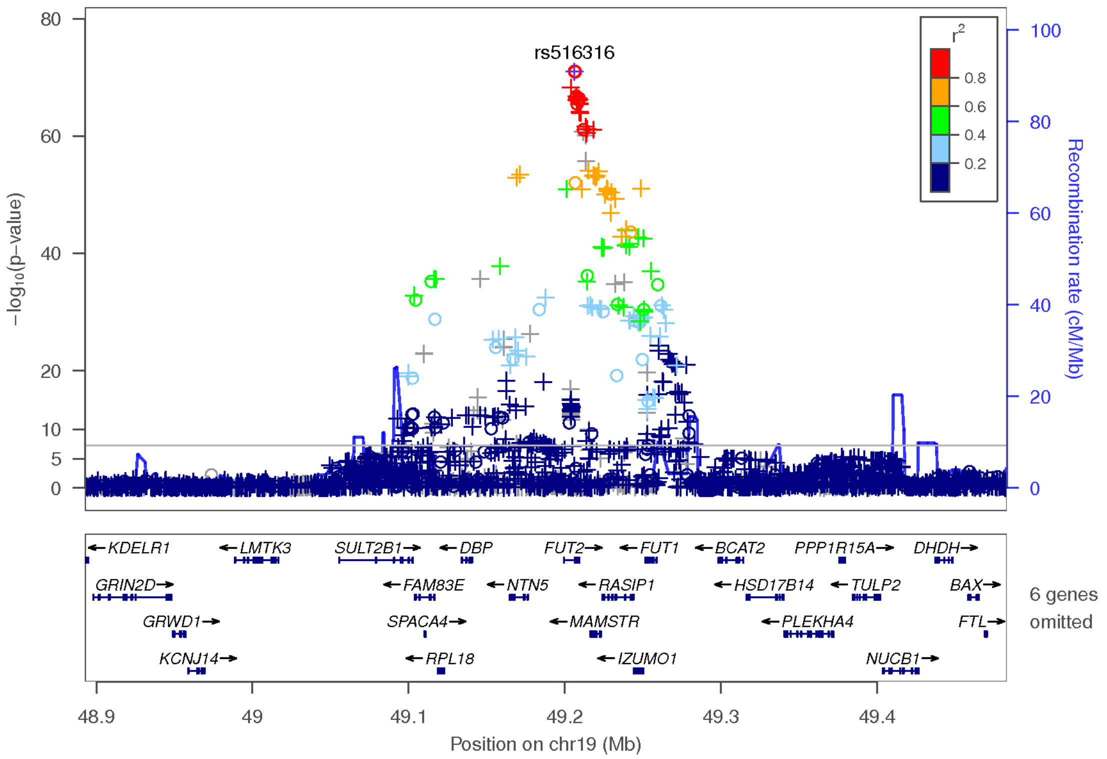

rs516316
−log₁₀(p−value)
Recombination rate (cM/Mb)
r²
0.8
0.6
0.4
0.2
KDELR1
LMTK3
SULT2B1
DBP
FUT2
FUT1
BCAT2
PPP1R15A
DHDH
GRIN2D
FAM83E
NTN5
RASIP1
HSD17B14
TULP2
BAX
GRWD1
SPACA4
MAMSTR
PLEKHA4
FTL
KCNJ14
RPL18
IZUMO1
NUCB1
6 genes omitted
48.9
49
49.1
49.2
49.3
49.4
Position on chr19 (Mb)

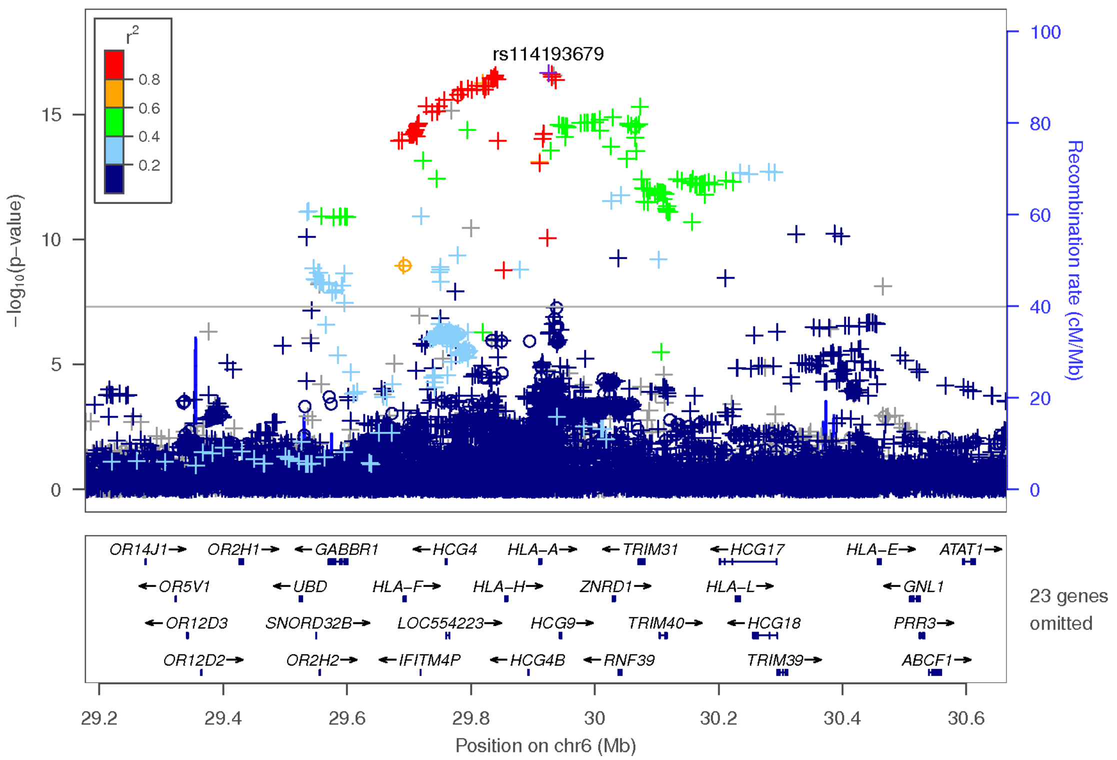
rs114193679
r2
0.8
0.6
0.4
0.2
−log10(p−value)
Recombination rate (cM/Mb)
OR14J1 OR2H1 GABBR1 HCG4 HLA−A TRIM31 HCG17 HLA−E ATAT1
OR5V1 UBD HLA−F HLA−H ZNRD1 HLA−L GNL1
OR12D3 SNORD32B LOC554223 HCG9 TRIM40 HCG18 PRR3
OR12D2 OR2H2 IFITM4P HCG4B RNF39 TRIM39 ABCF1
23 genes omitted
Position on chr6 (Mb)

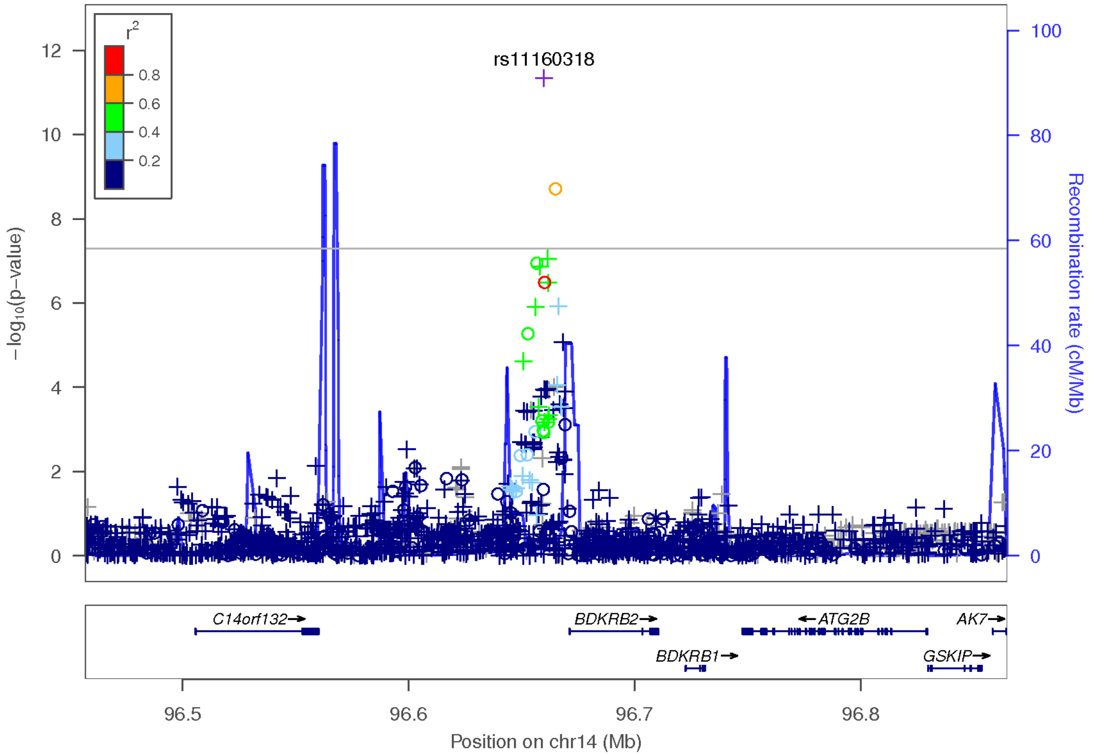
r2
0.8
0.6
0.4
0.2
rs11160318
−log10(p−value)
Recombination rate (cM/Mb)
C14orf132
BDKRB2
ATG2B
AK7
BDKRB1
GSKIP
96.5
96.6
96.7
96.8
Position on chr14 (Mb)

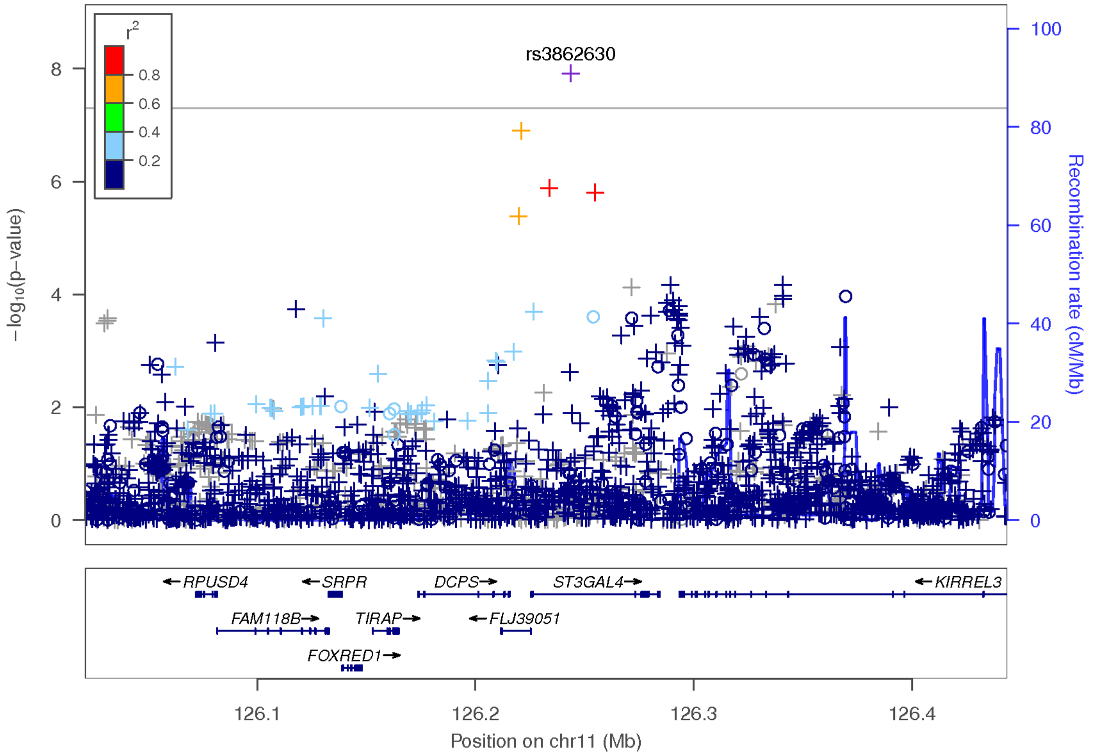
r2
0.8
0.6
0.4
0.2
rs3862630
−log10(p−value)
Recombination rate (cM/Mb)
RPUSD4
SRPR
DCPS
ST3GAL4
KIRREL3
FAM118B
TIRAP
FLJ39051
FOXRED1
126.1
126.2
126.3
126.4
Position on chr11 (Mb)

Hepatitis A:

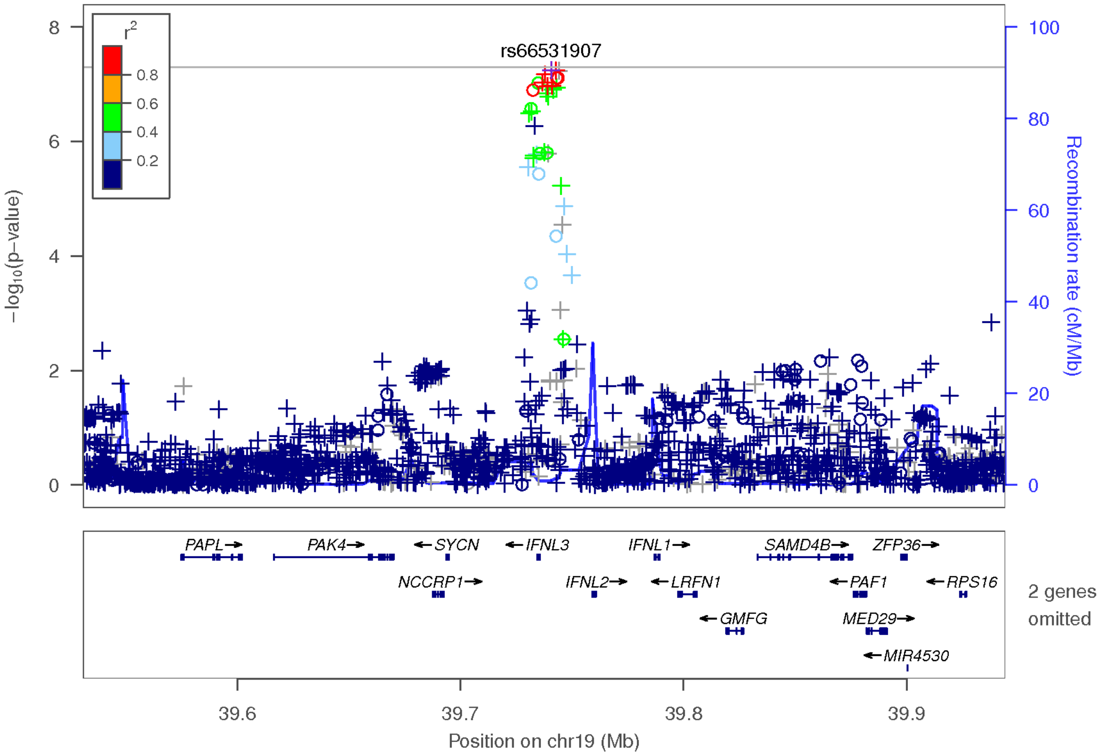
rs66531907
r2
0.8
0.6
0.4
0.2
−log10(p−value)
Recombination rate (cM/Mb)
PAPL
PAK4
SYCN
IFNL3
IFNL1
SAMD4B
ZFP36
NCCRP1
IFNL2
LRFN1
PAF1
RPS16
GMFG
MED29
MIR4530
2 genes omitted
39.6
39.7
39.8
39.9
Position on chr19 (Mb)

Hepatitis B:

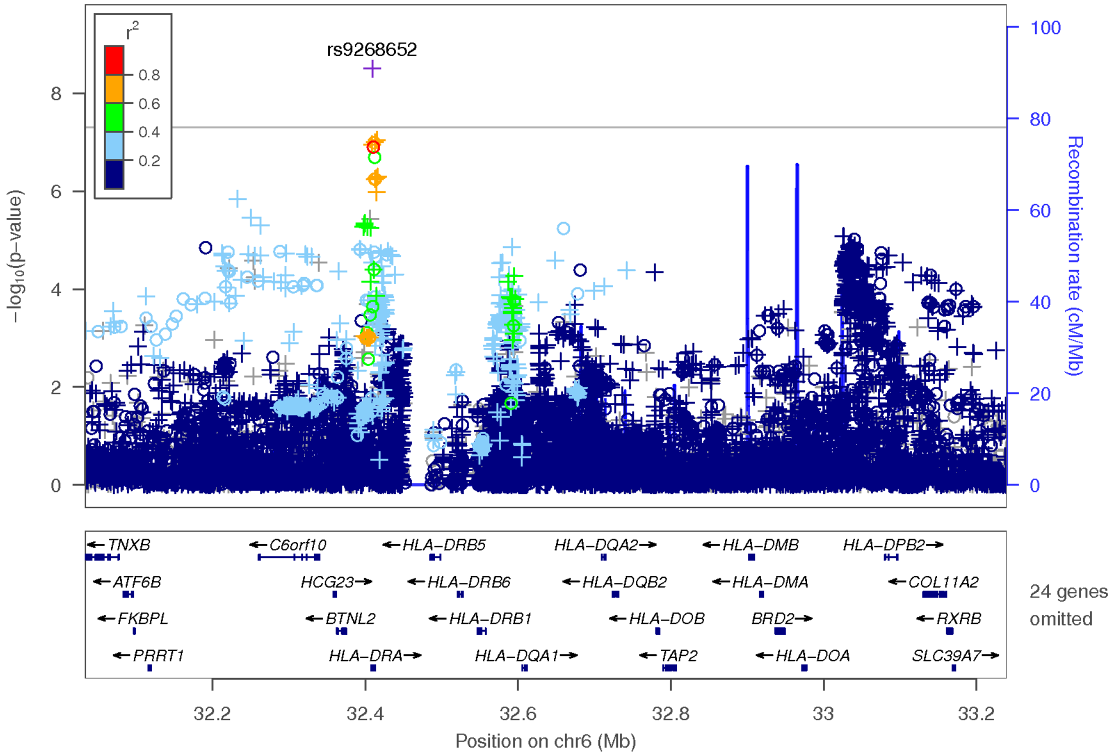
rs9268652
r2
0.8
0.6
0.4
0.2
−log10(p−value)
Recombination rate (cM/Mb)
TNXB
ATF6B
FKBPL
PRRT1
C6orf10
HCG23
BTNL2
HLA−DRA
HLA−DRB5
HLA−DRB6
HLA−DRB1
HLA−DQA1
HLA−DQA2
HLA−DQB2
HLA−DOB
TAP2
HLA−DMB
HLA−DMA
BRD2
HLA−DOA
HLA−DPB2
COL11A2
RXRB
SLC39A7
24 genes omitted
Position on chr6 (Mb)

Plantar warts:

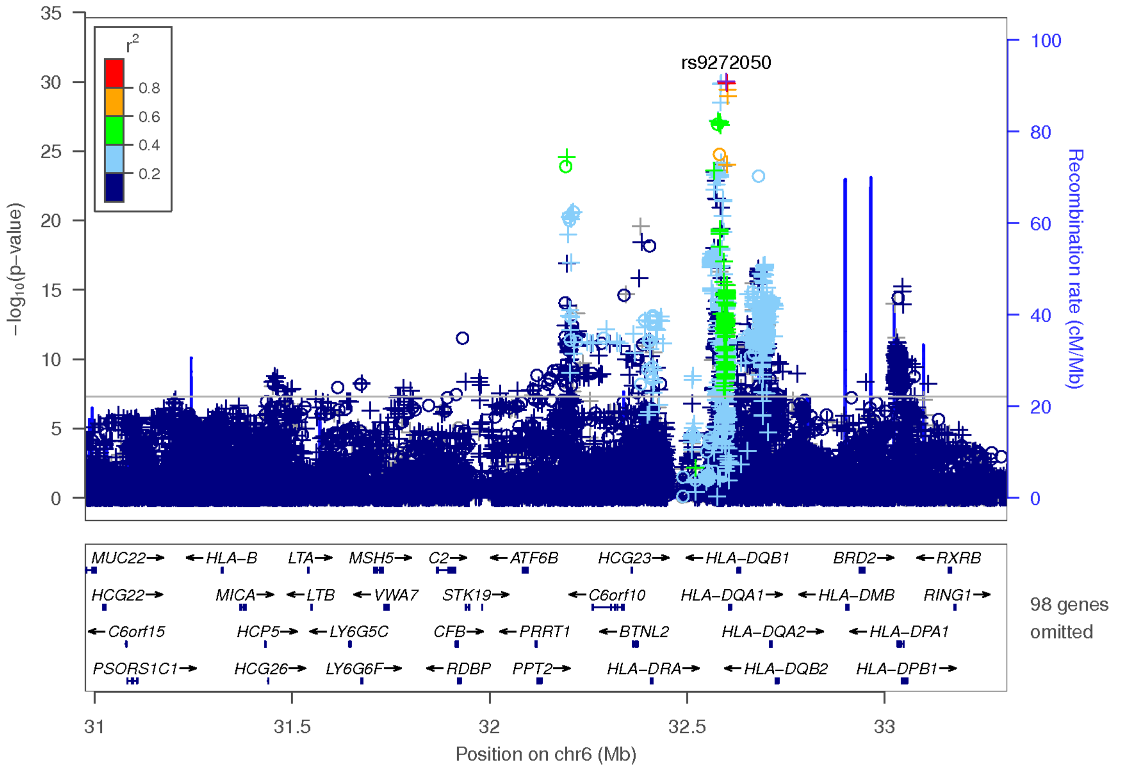

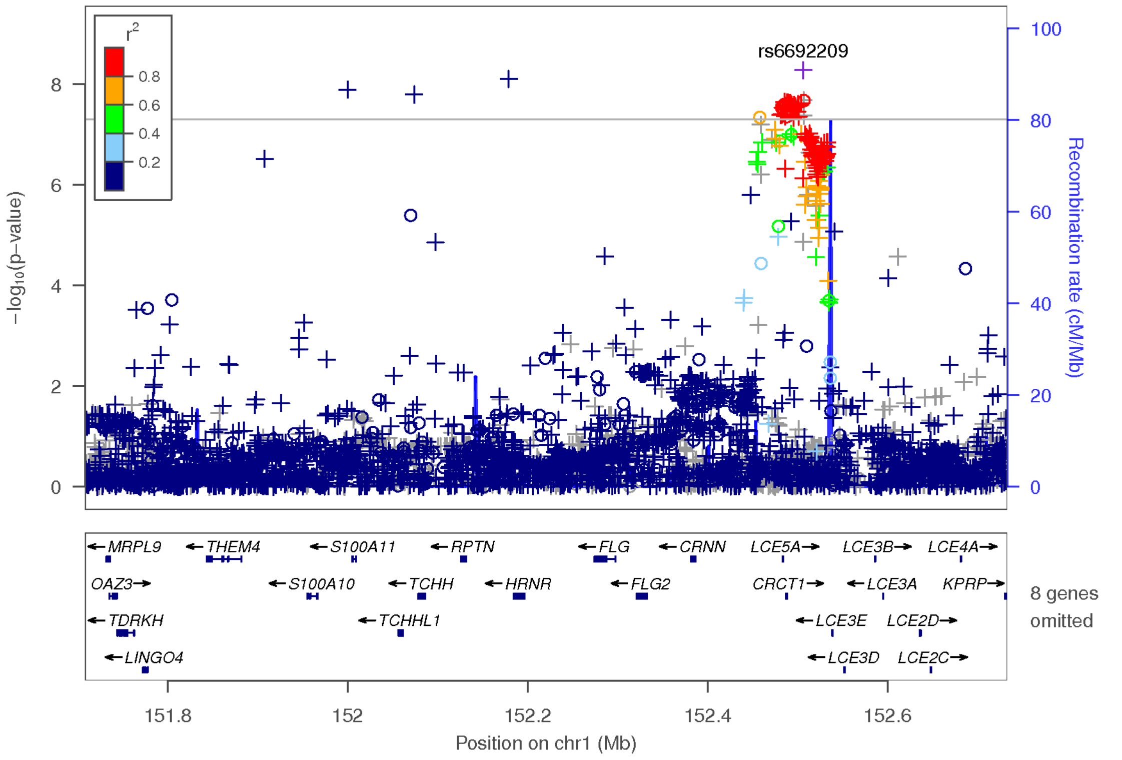

Positive TB test:

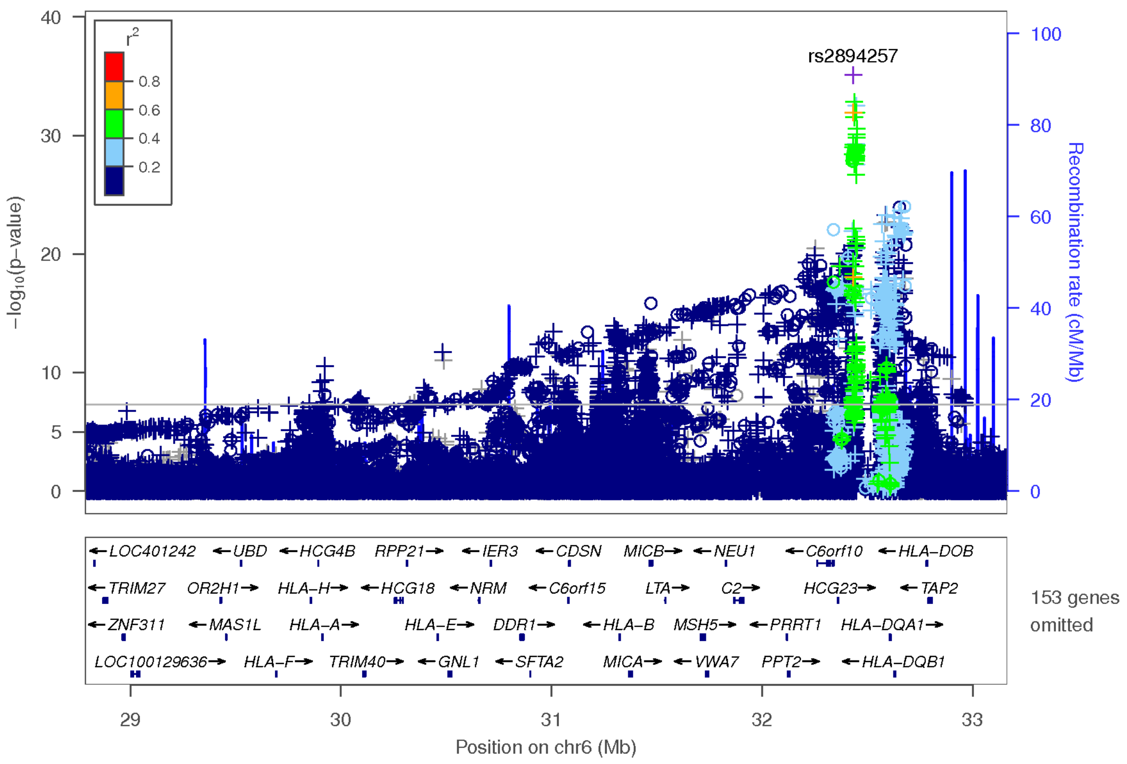

Strep throat:

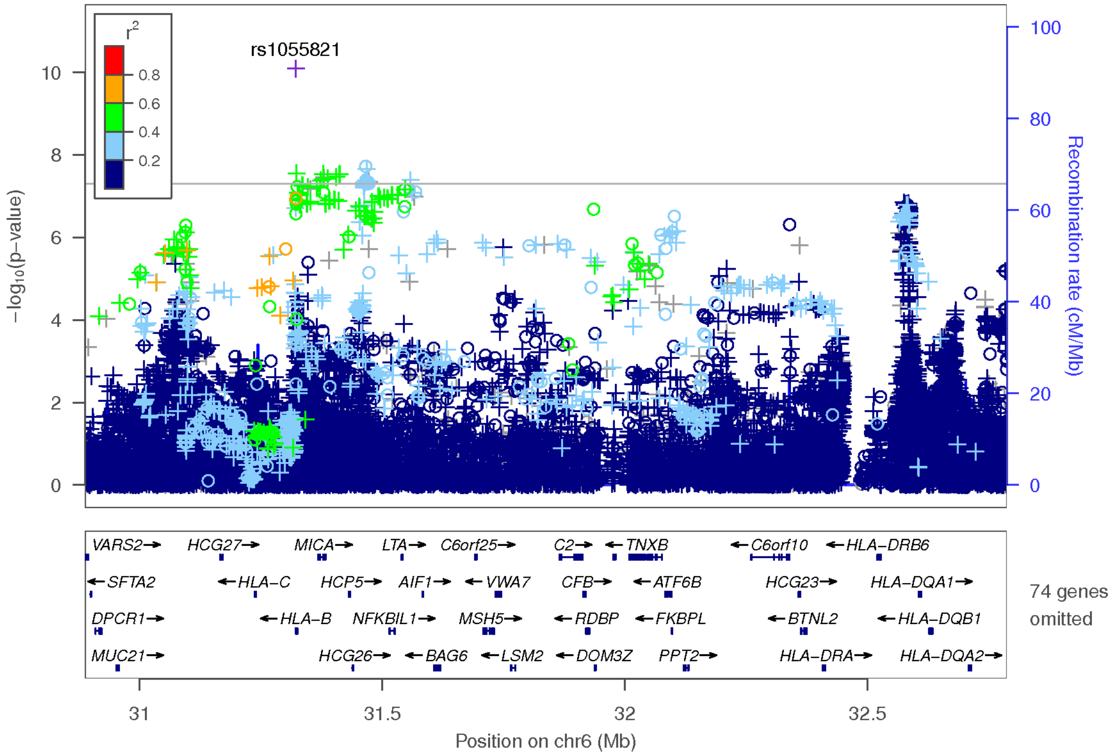

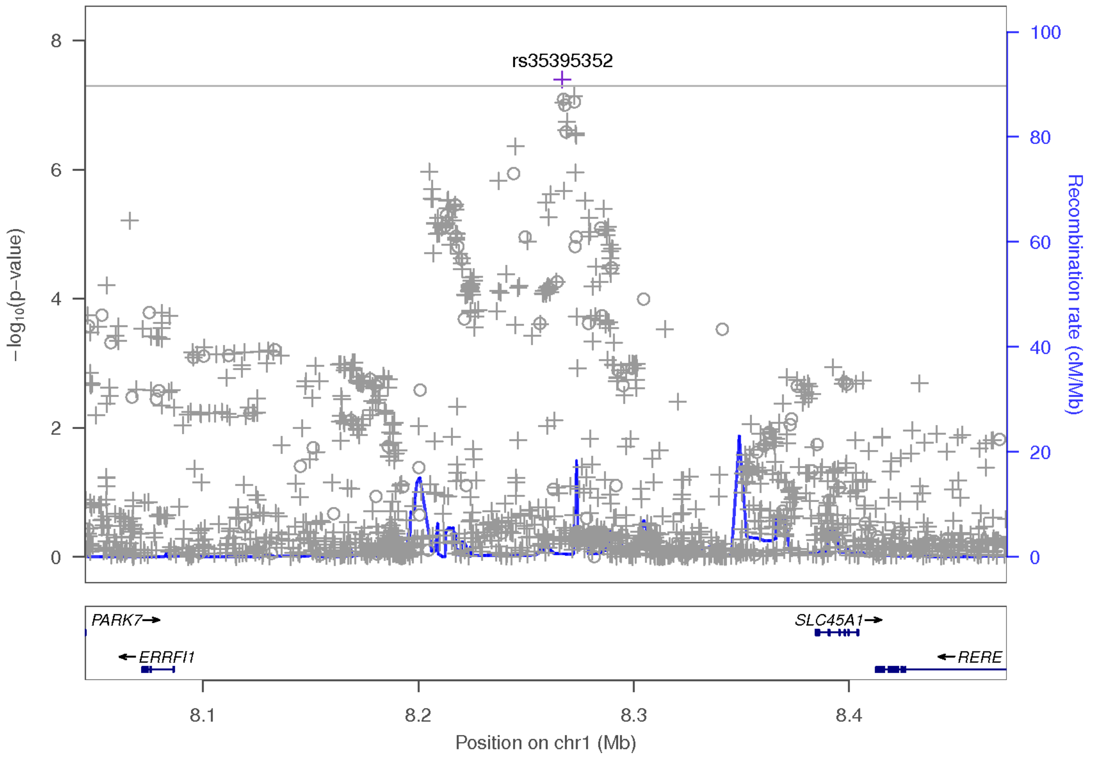

Scarlet fever:

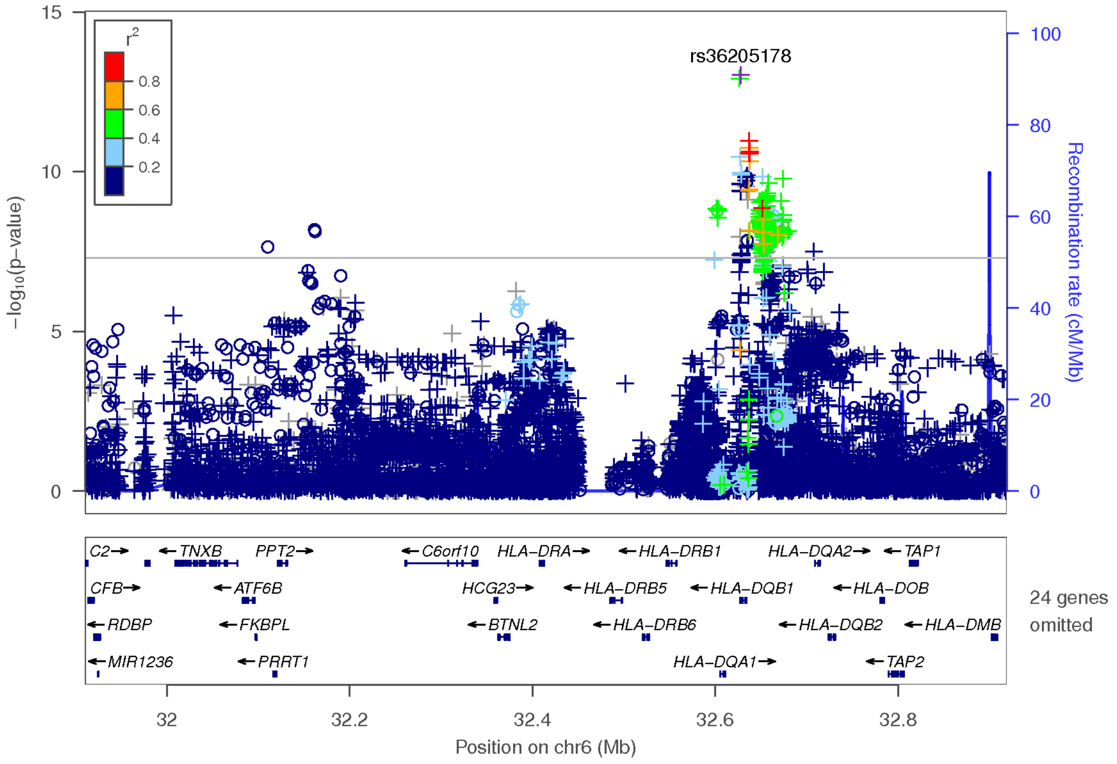

Pneumonia:

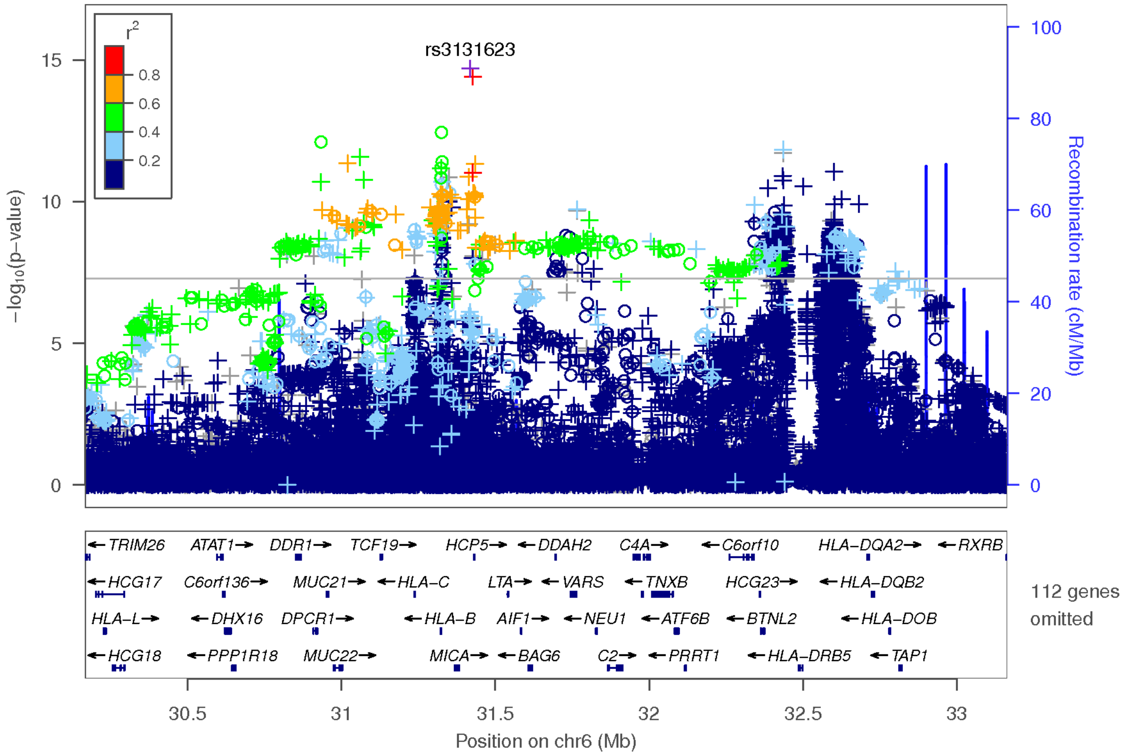

r2
0.8
0.6
0.4
0.2
rs3131623
15
10
5
0
−log10(p−value)
100
80
60
40
20
0
Recombination rate (cM/Mb)
TRIM26
ATAT1
DDR1
TCF19
HCP5
DDAH2
C4A
C6orf10
HLA−DQA2
RXRB
HCG17
C6orf136
MUC21
HLA−C
LTA
VARS
TNXB
HCG23
HLA−DQB2
HLA−L
DHX16
DPCR1
HLA−B
AIF1
NEU1
ATF6B
BTNL2
HLA−DOB
HCG18
PPP1R18
MUC22
MICA
BAG6
C2
PRRT1
HLA−DRB5
TAP1
112 genes omitted
30.5
31
31.5
32
32.5
33
Position on chr6 (Mb)

Bacterial meningitis:

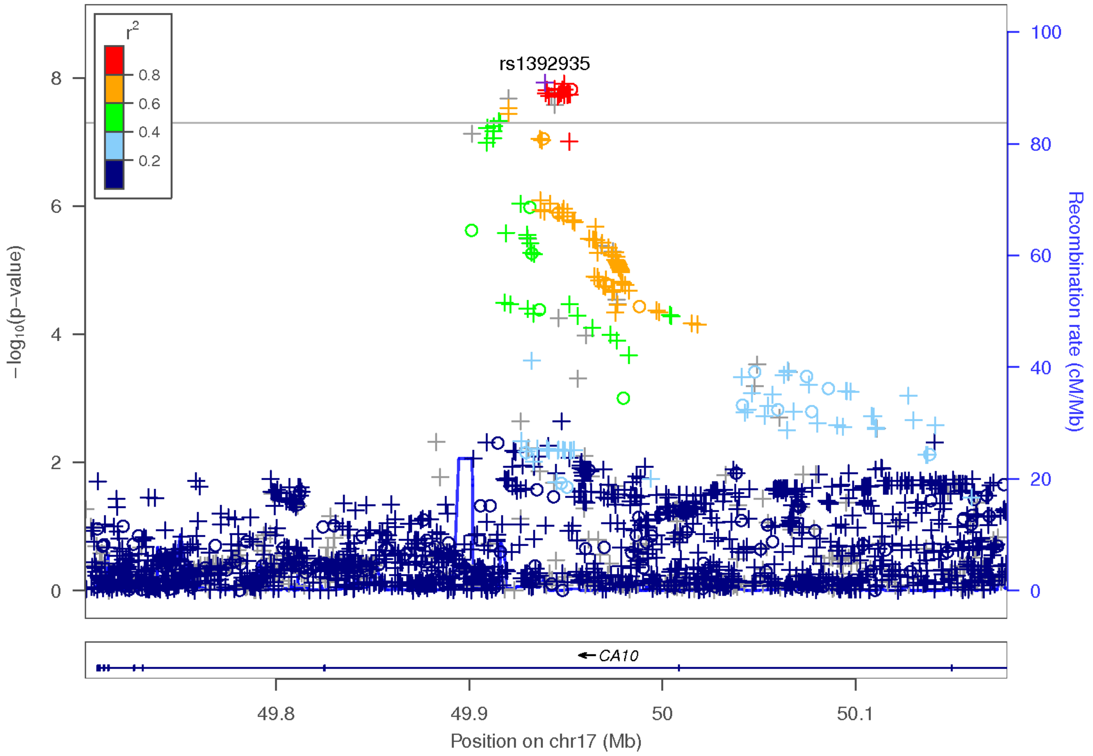

Yeast infection:

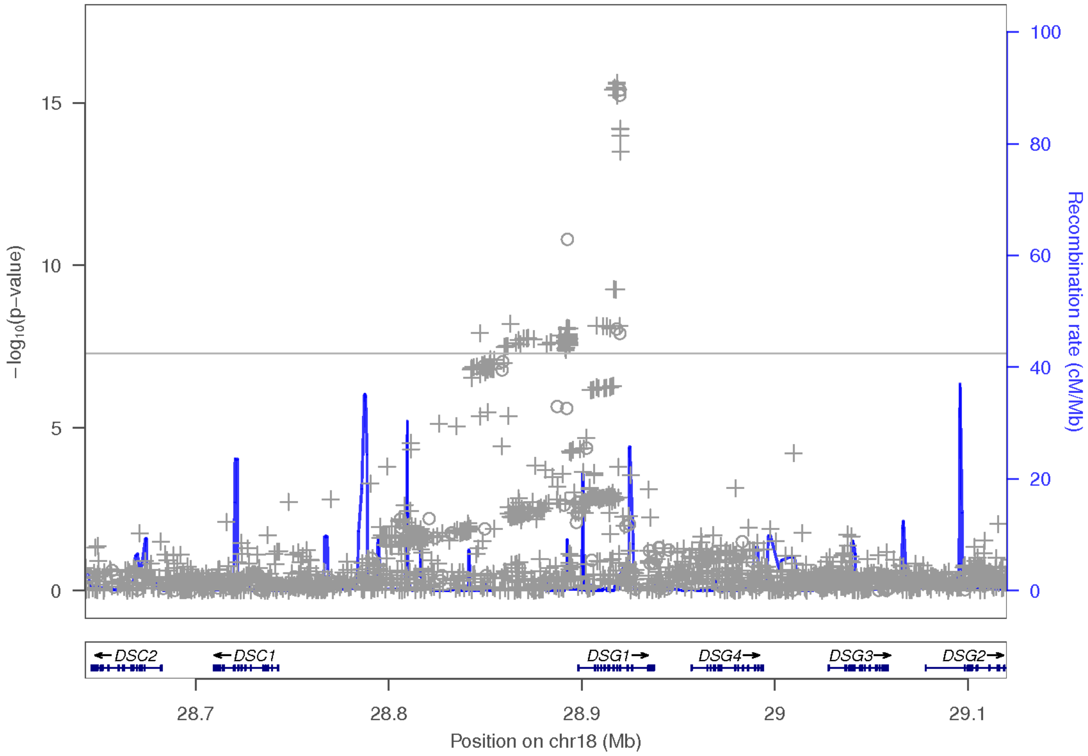

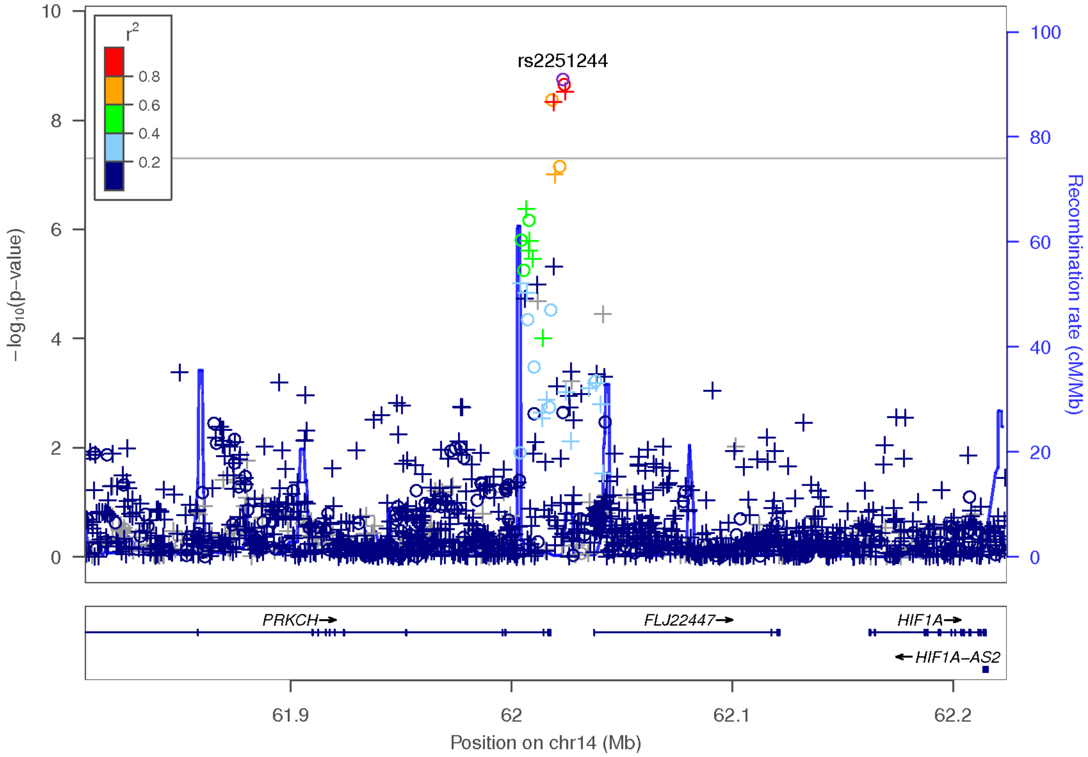

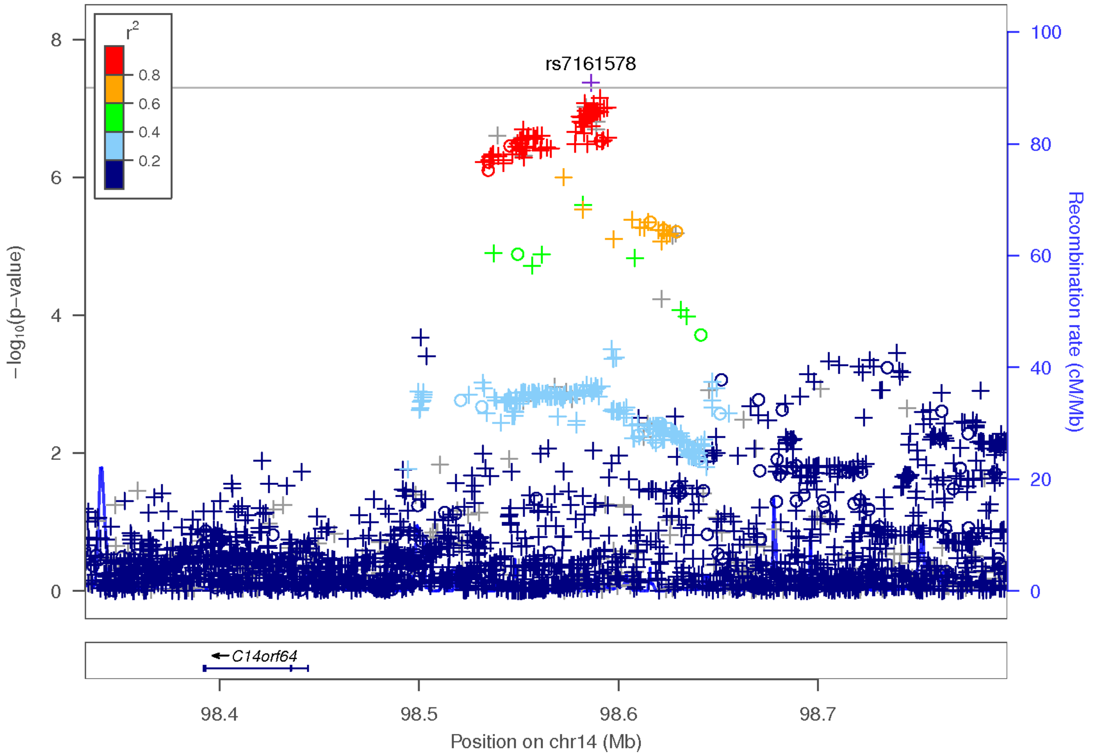

Urinary tract infection frequency:

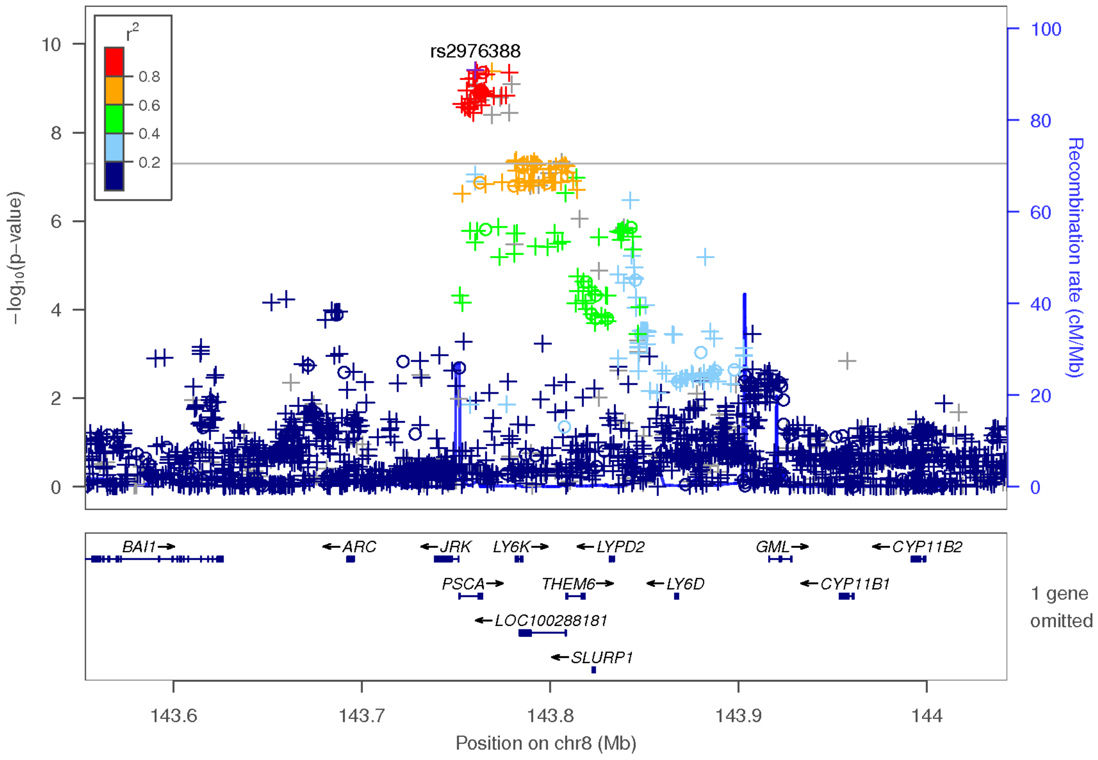

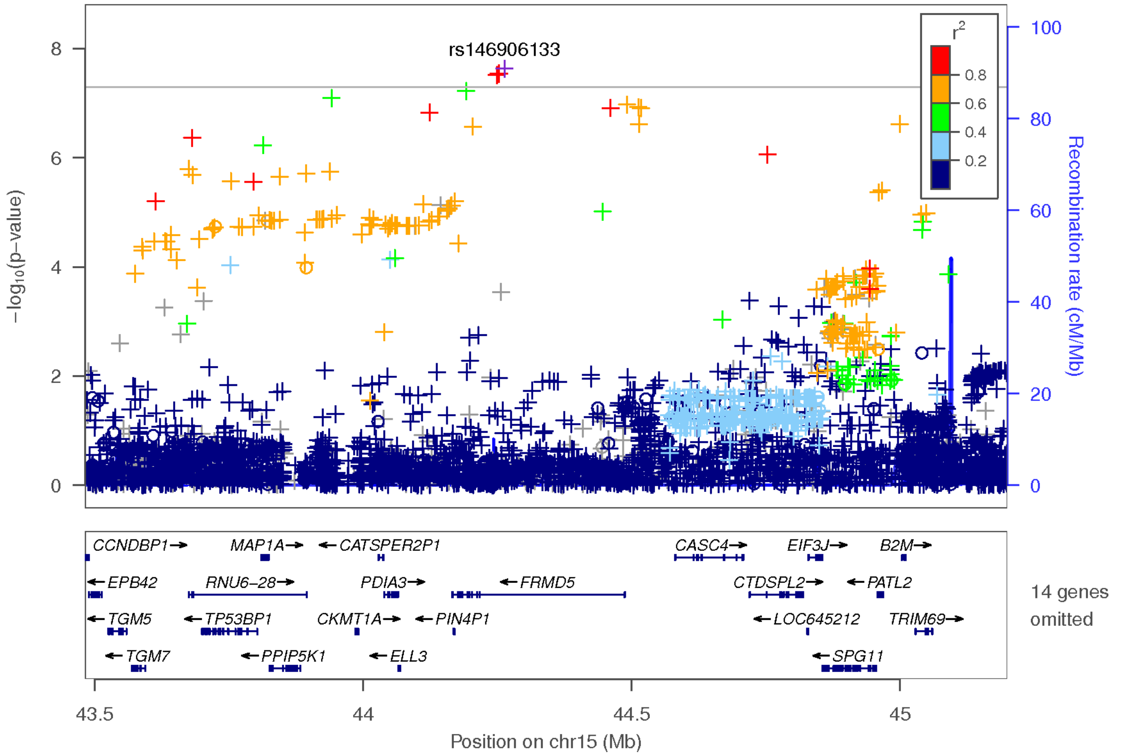

Tonsillectomy:

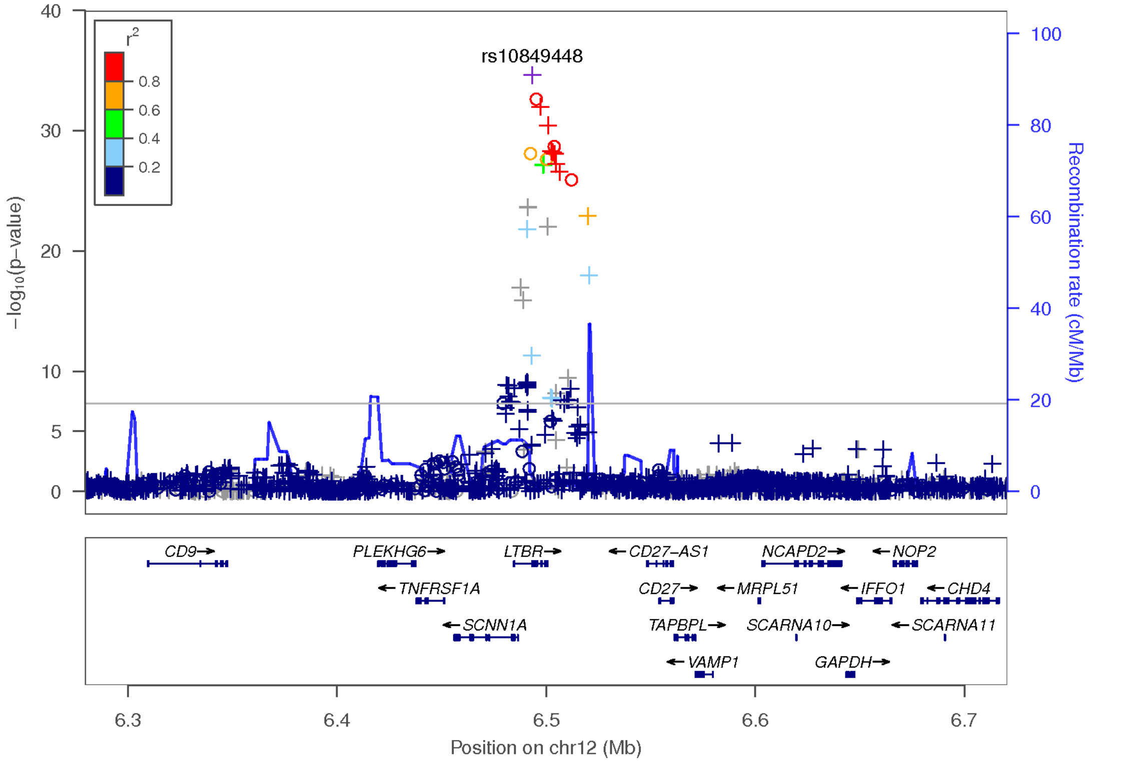

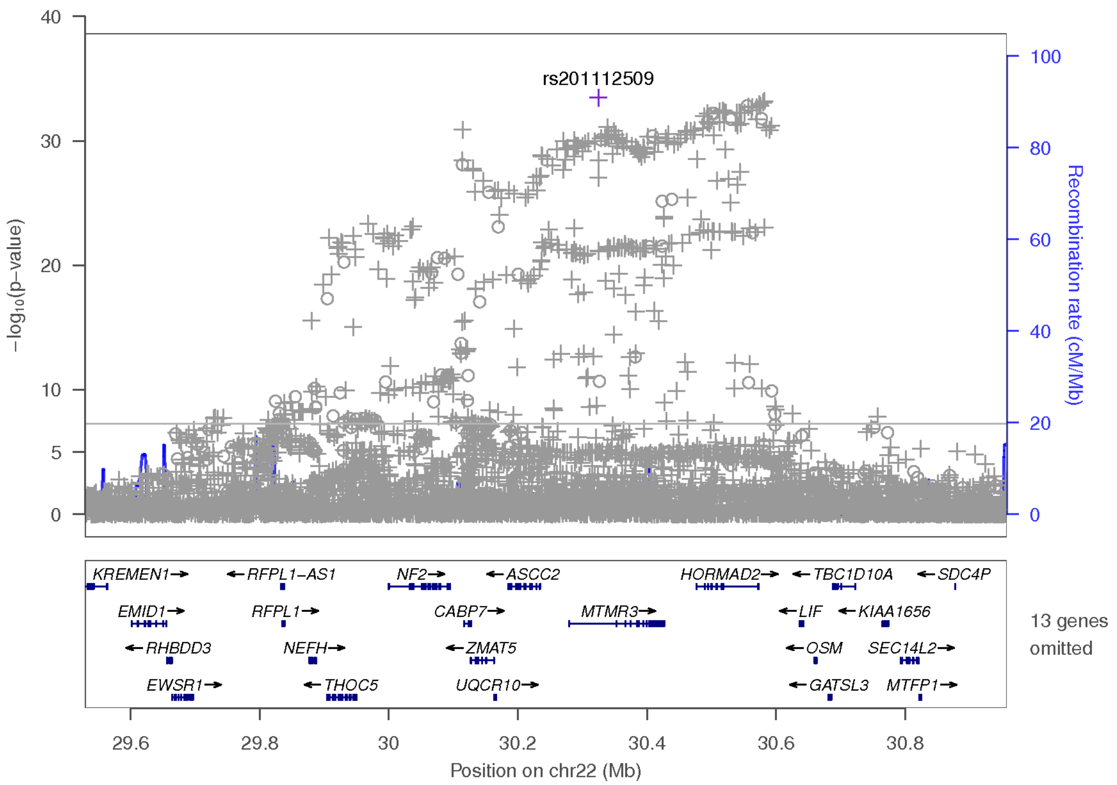
rs201112509
−log10(p−value)
Recombination rate (cM/Mb)
KREMEN1
RFPL1−AS1
NF2
ASCC2
HORMAD2
TBC1D10A
SDC4P
EMID1
RFPL1
CABP7
MTMR3
LIF
KIAA1656
RHBDD3
NEFH
ZMAT5
OSM
SEC14L2
EWSR1
THOC5
UQCR10
GATSL3
MTFP1
13 genes omitted
Position on chr22 (Mb)

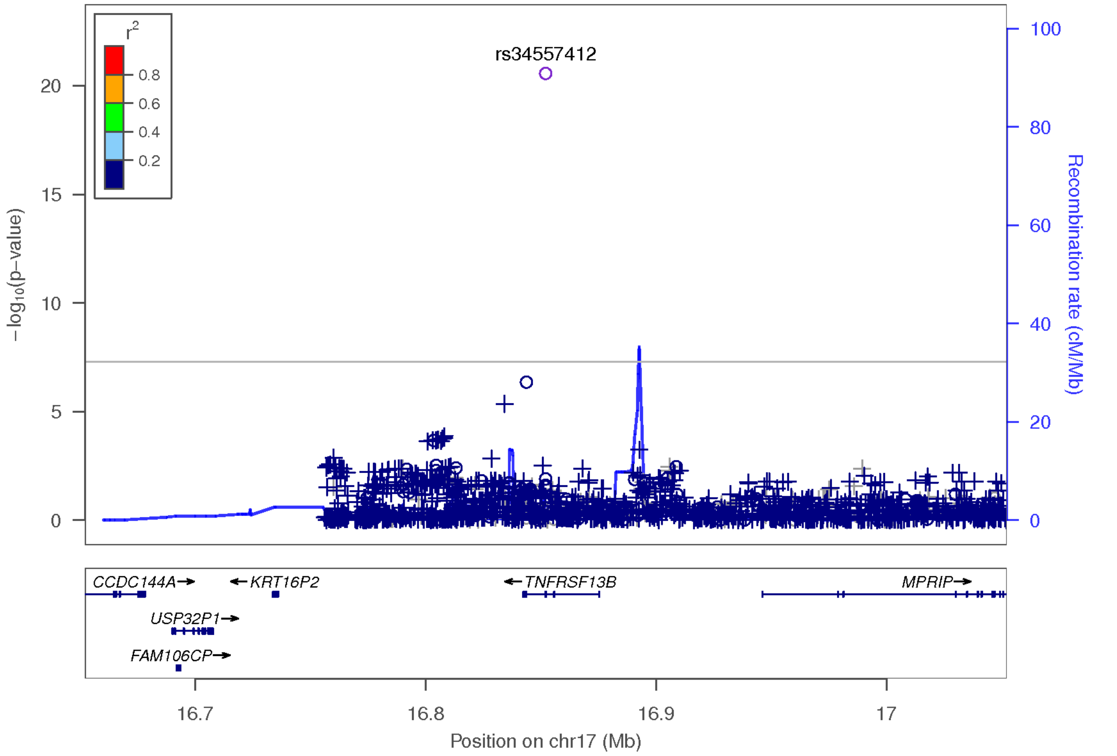
r2
rs34557412
−log10(p−value)
Recombination rate (cM/Mb)
CCDC144A
KRT16P2
TNFRSF13B
MPRIP
USP32P1
FAM106CP
Position on chr17 (Mb)

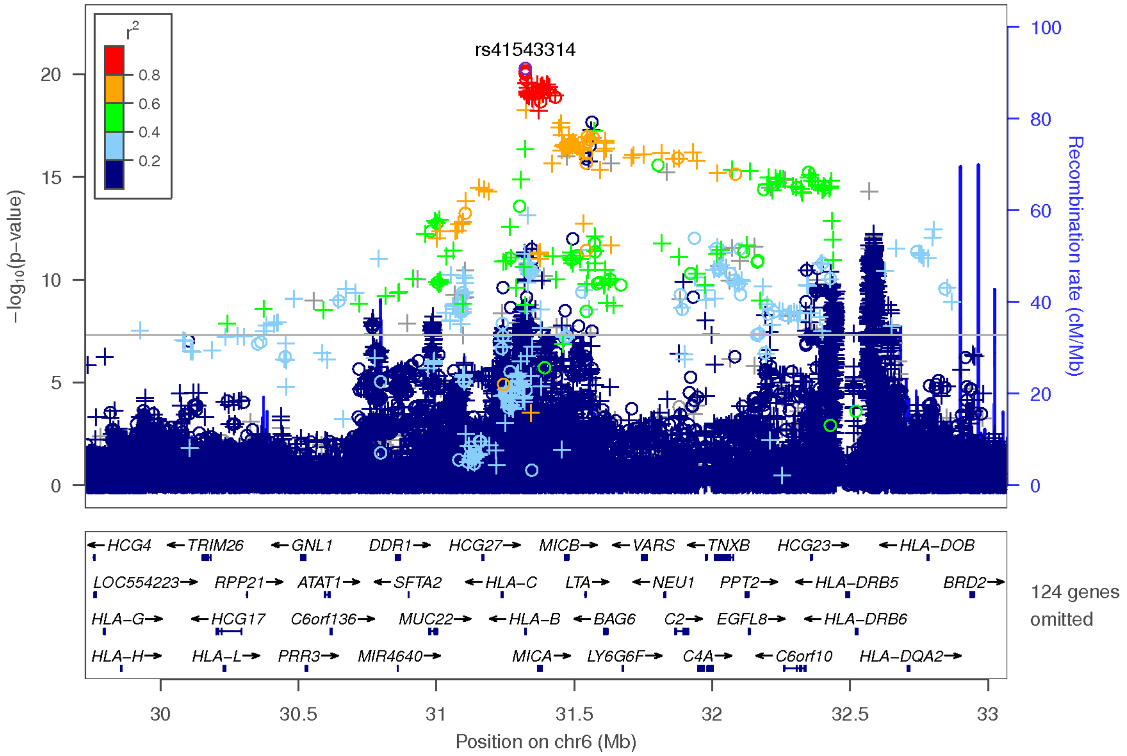
r²
0.8
0.6
0.4
0.2
rs41543314
−log₁₀(p−value)
Recombination rate (cM/Mb)
HCG4
TRIM26
GNL1
DDR1
HCG27
MICB
VARS
TNXB
HCG23
HLA−DOB
LOC554223
RPP21
ATAT1
SFTA2
HLA−C
LTA
NEU1
PPT2
HLA−DRB5
BRD2
HLA−G
HCG17
C6orf136
MUC22
HLA−B
BAG6
C2
EGFL8
HLA−DRB6
HLA−H
HLA−L
PRR3
MIR4640
MICA
LY6G6F
C4A
C6orf10
HLA−DQA2
124 genes omitted
Position on chr6 (Mb)

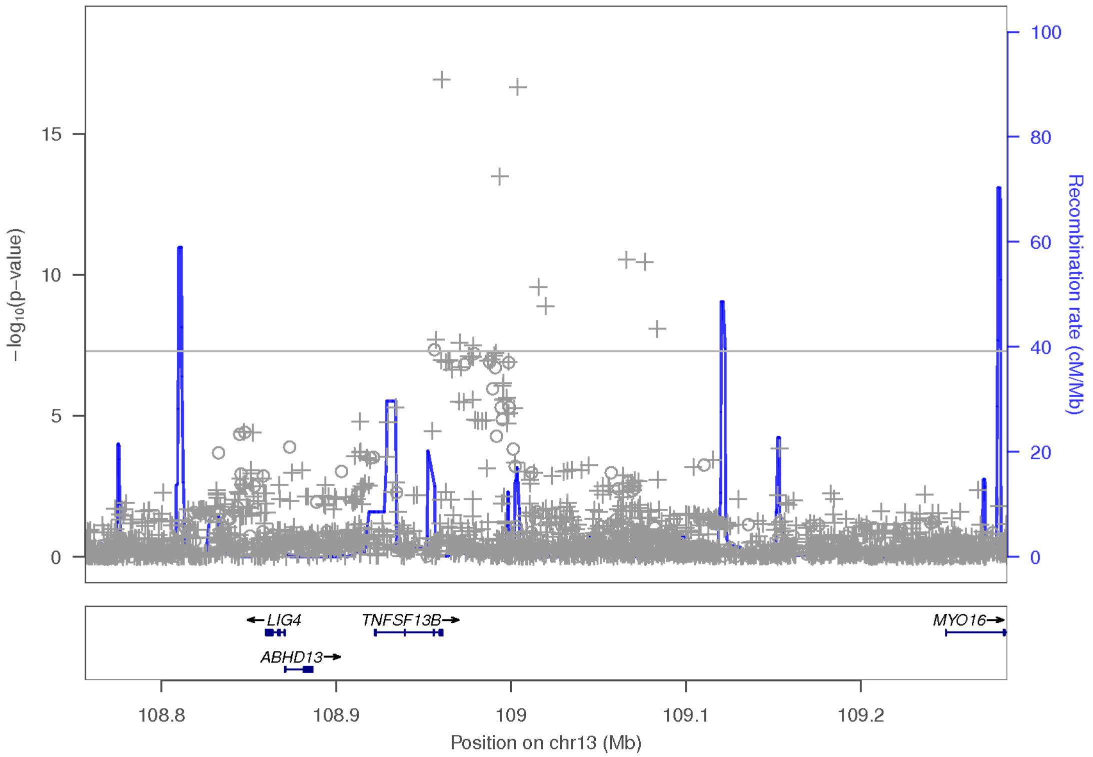
−log₁₀(p−value)
Recombination rate (cM/Mb)
LIG4
TNFSF13B
MYO16
ABHD13
Position on chr13 (Mb)

Childhood ear infection:

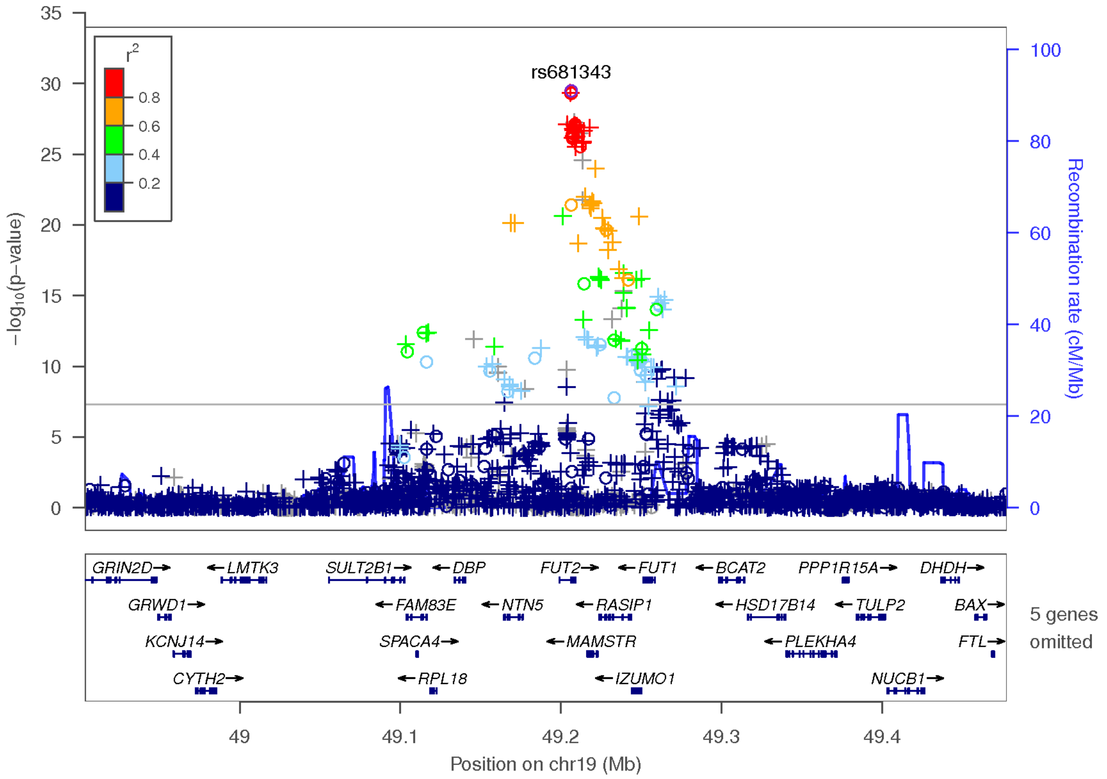

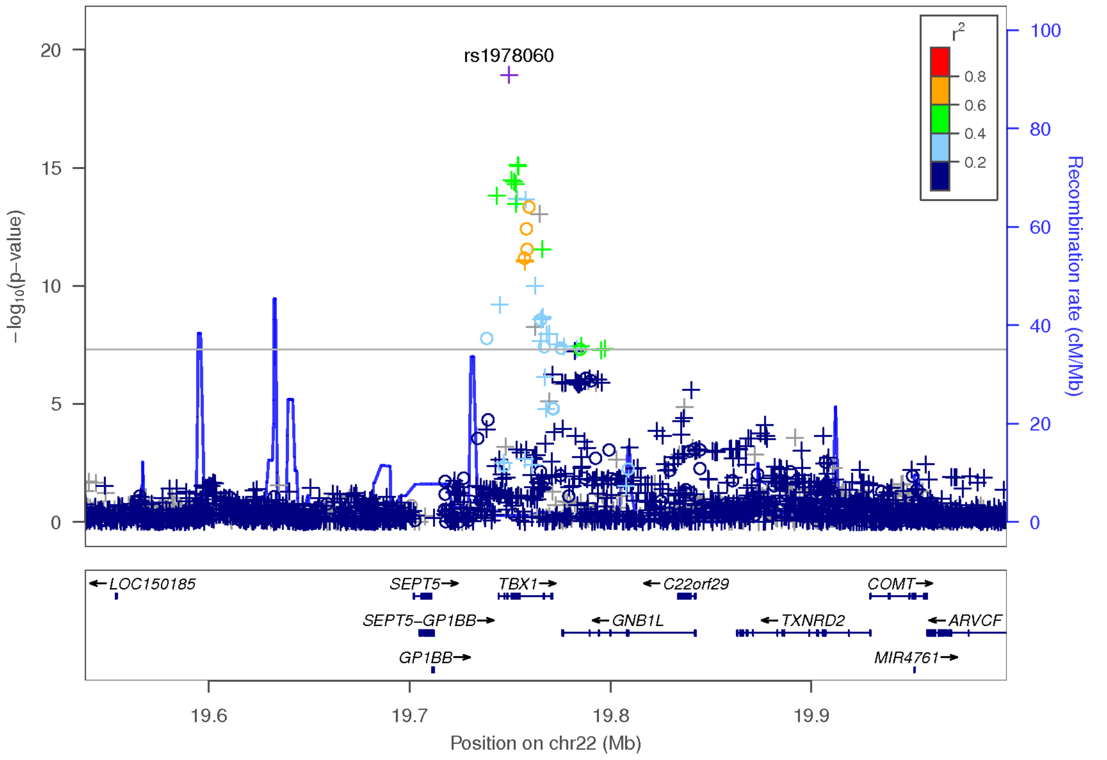

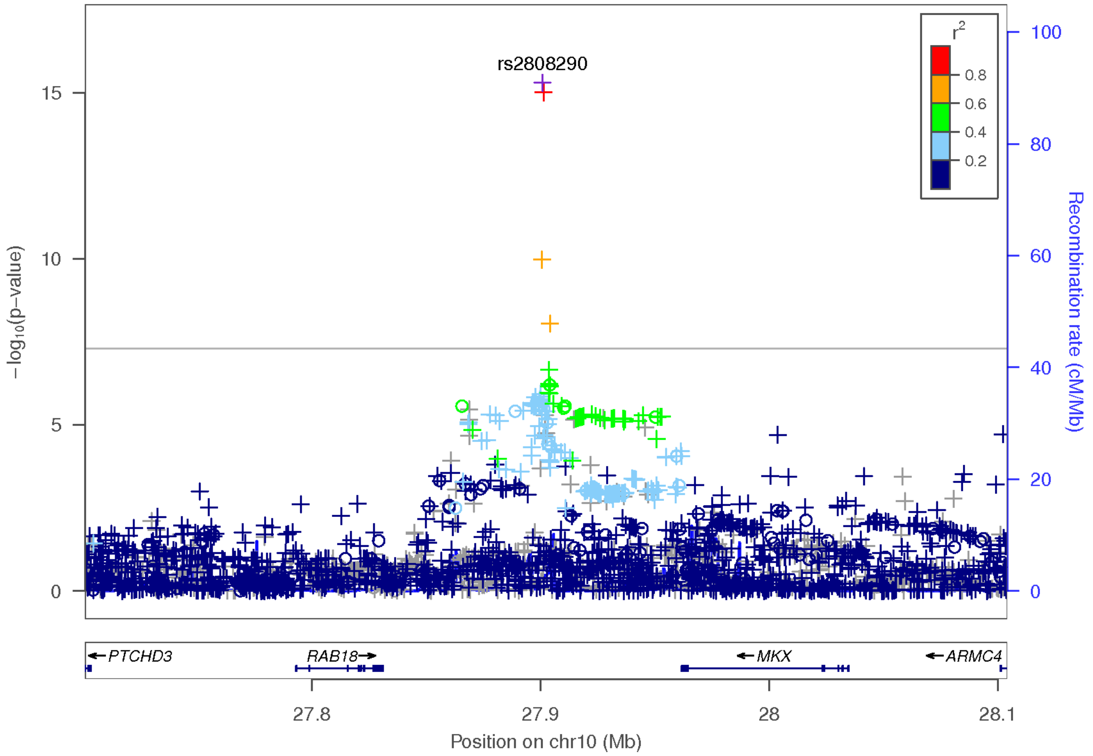
rs2808290
r2
0.8
0.6
0.4
0.2
−log10(p−value)
Recombination rate (cM/Mb)
PTCHD3
RAB18
MKX
ARMC4
27.8
27.9
28
28.1
Position on chr10 (Mb)

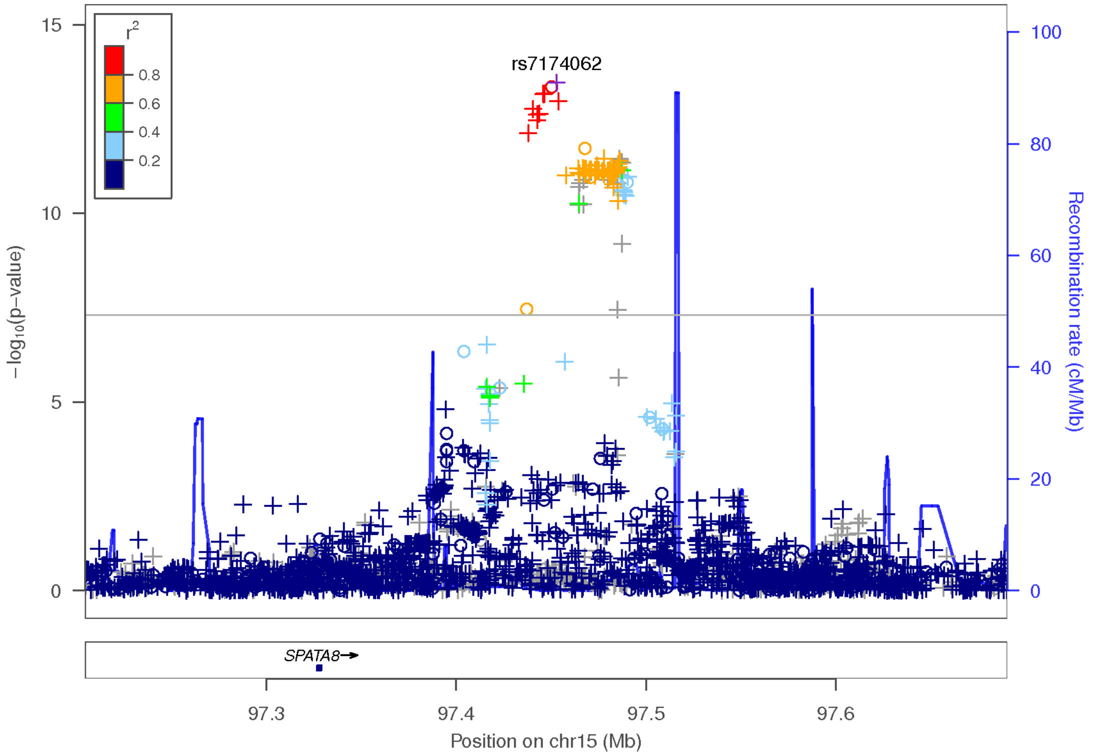
rs7174062
r2
0.8
0.6
0.4
0.2
−log10(p−value)
Recombination rate (cM/Mb)
SPATA8
97.3
97.4
97.5
97.6
Position on chr15 (Mb)

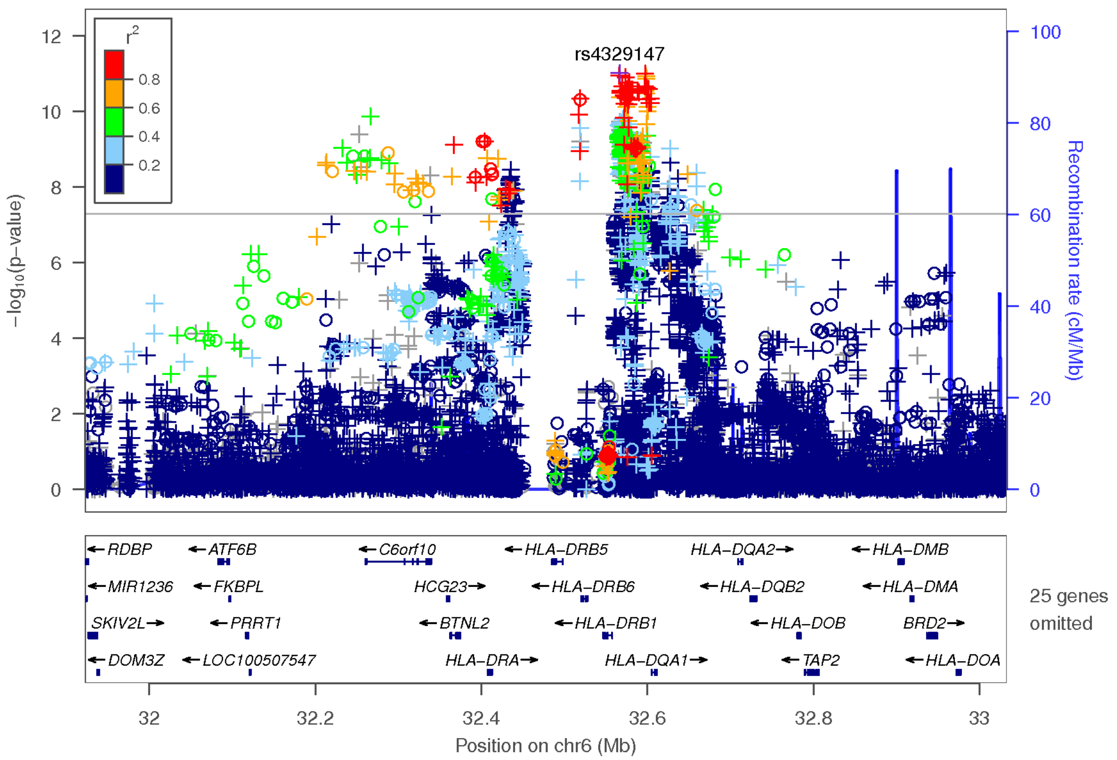
r2
0.8
0.6
0.4
0.2
rs4329147
−log10(p−value)
Recombination rate (cM/Mb)
RDBP
ATF6B
C6orf10
HLA−DRB5
HLA−DQA2
HLA−DMB
MIR1236
FKBPL
HCG23
HLA−DRB6
HLA−DQB2
HLA−DMA
SKIV2L
PRRT1
BTNL2
HLA−DRB1
HLA−DOB
BRD2
DOM3Z
LOC100507547
HLA−DRA
HLA−DQA1
TAP2
HLA−DOA
25 genes omitted
Position on chr6 (Mb)

Supplementary Figure 3: Regional plots of fine-mapping HLA signals

–log10 transformed *p* values are shown in green circles for SNPs, pink diamonds for amino acids and blue squares for HLA alleles. The purple dotted line corresponds to $p=5x10^{-8}$, which are the significance thresholds for fine mapping. Results above this threshold are shown with black borders. The plotted *p* values are conditional *p* values after iteratively conditioning on the top significant associations.

Chickenpox:

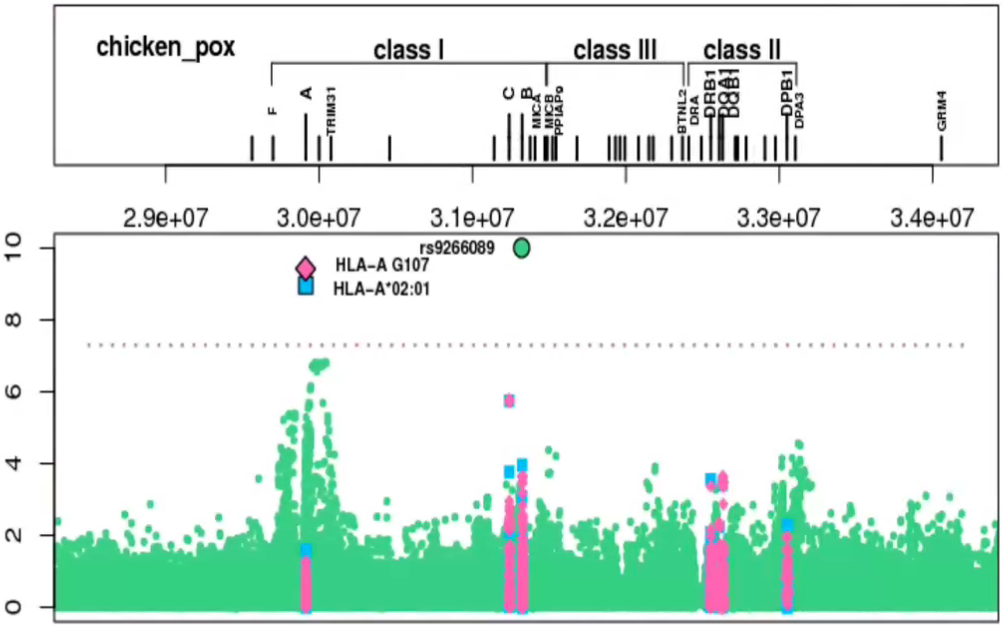

Shingles:

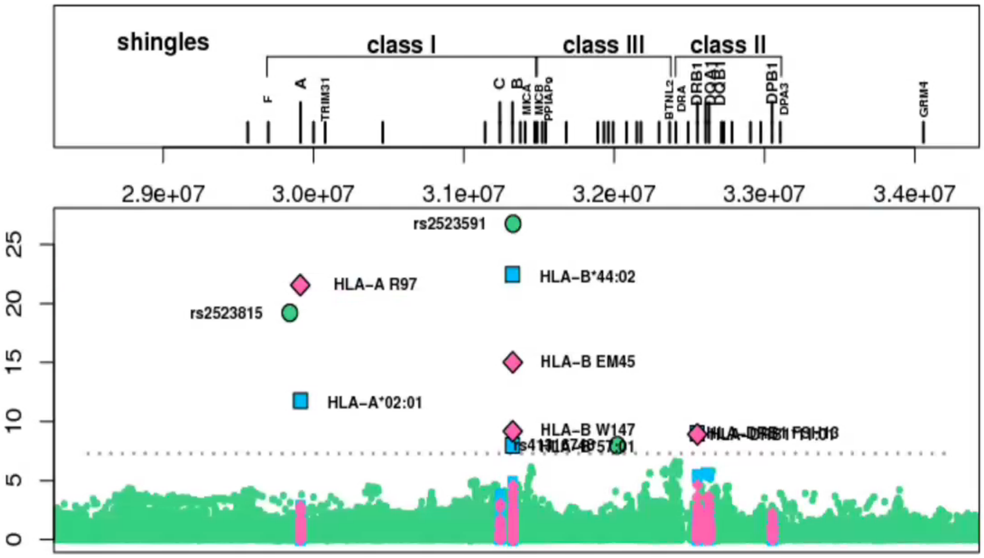

Cold sores:

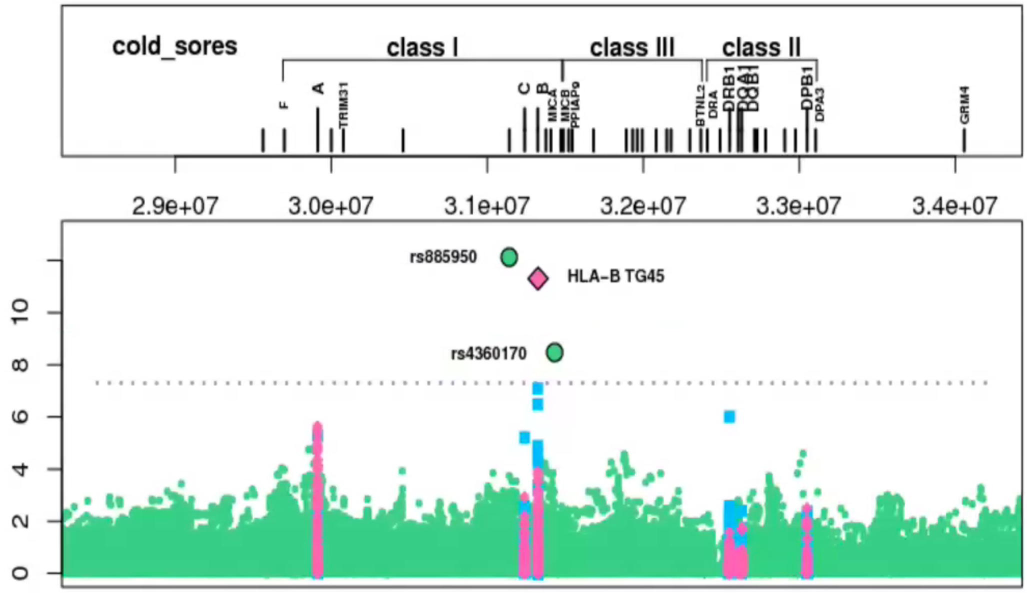

Mononucleosis:

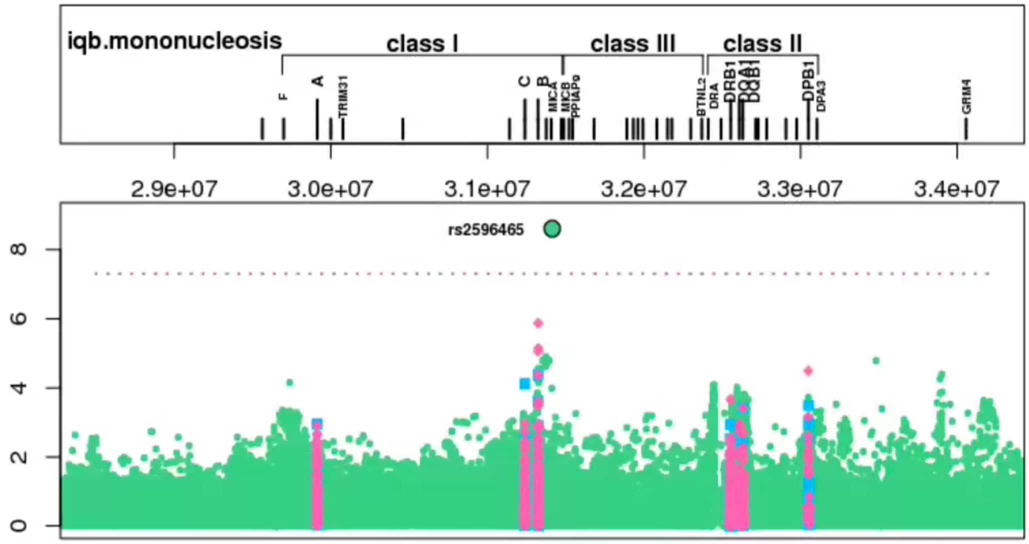

Mumps:

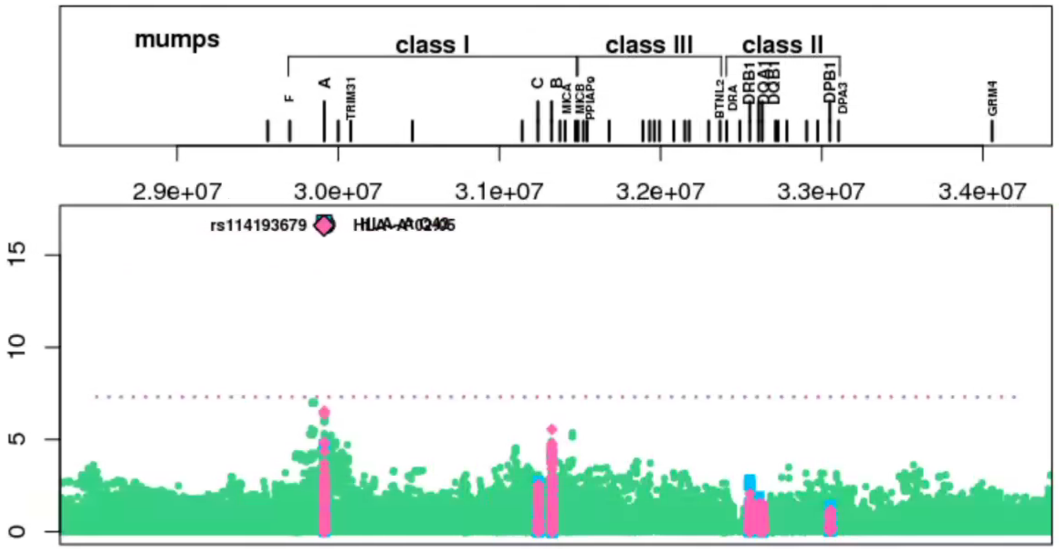

Hepatitis B:

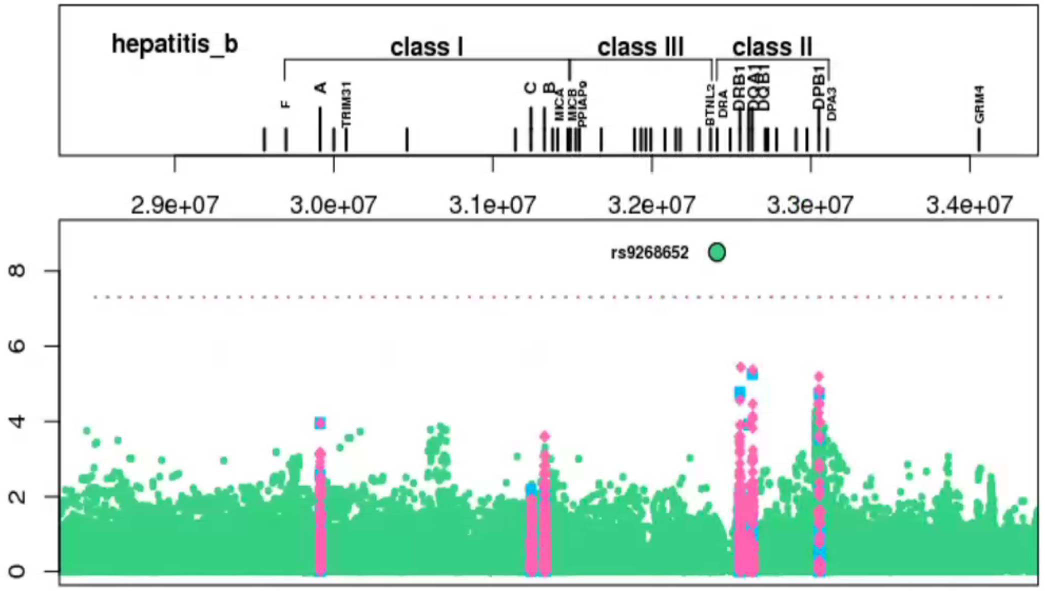

Plantar warts:

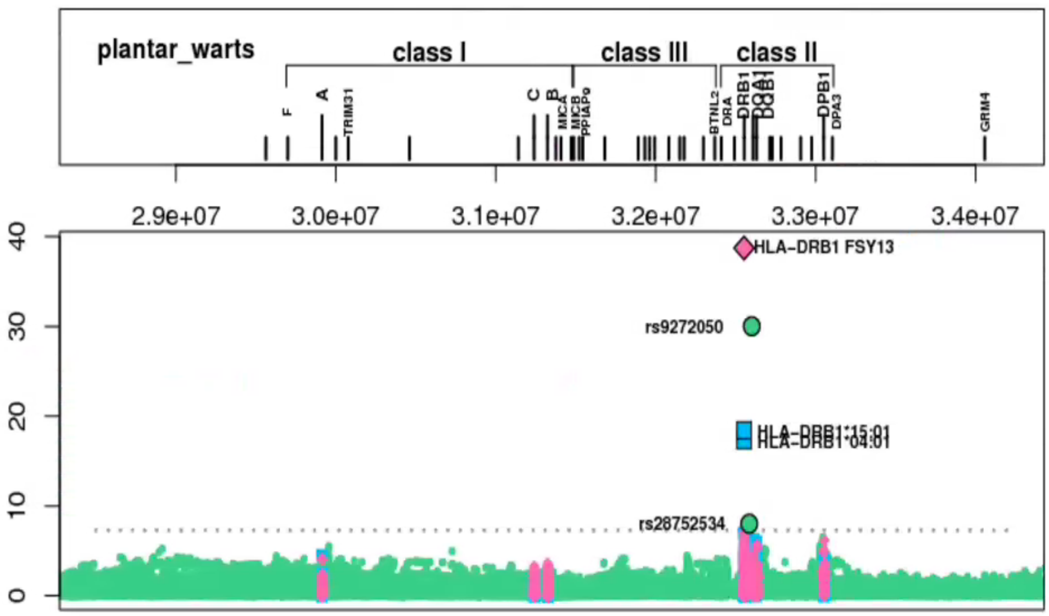

Positive TB test:

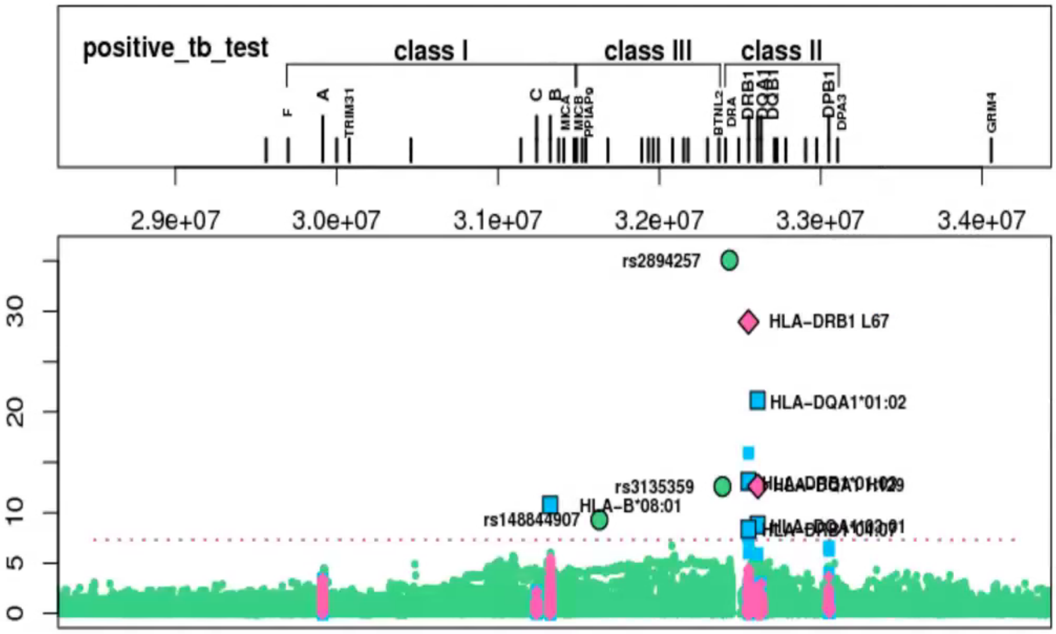

Strep throat:

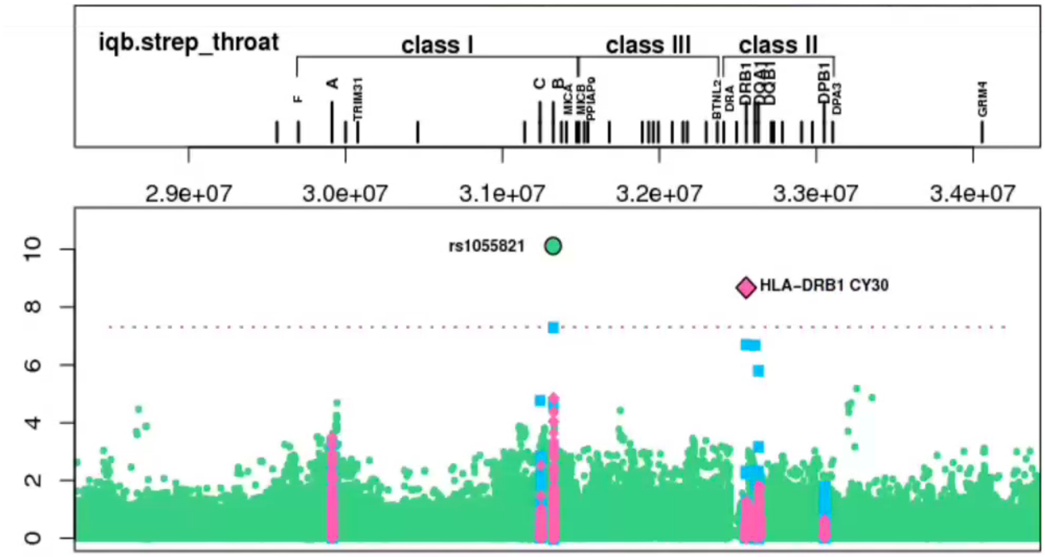

Scarlet fever:

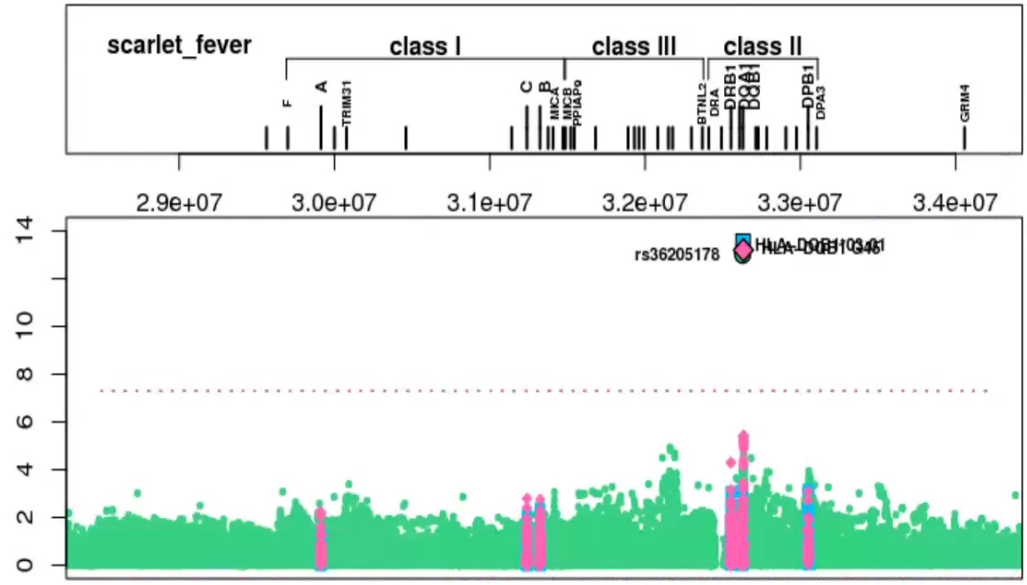

Pneumonia:

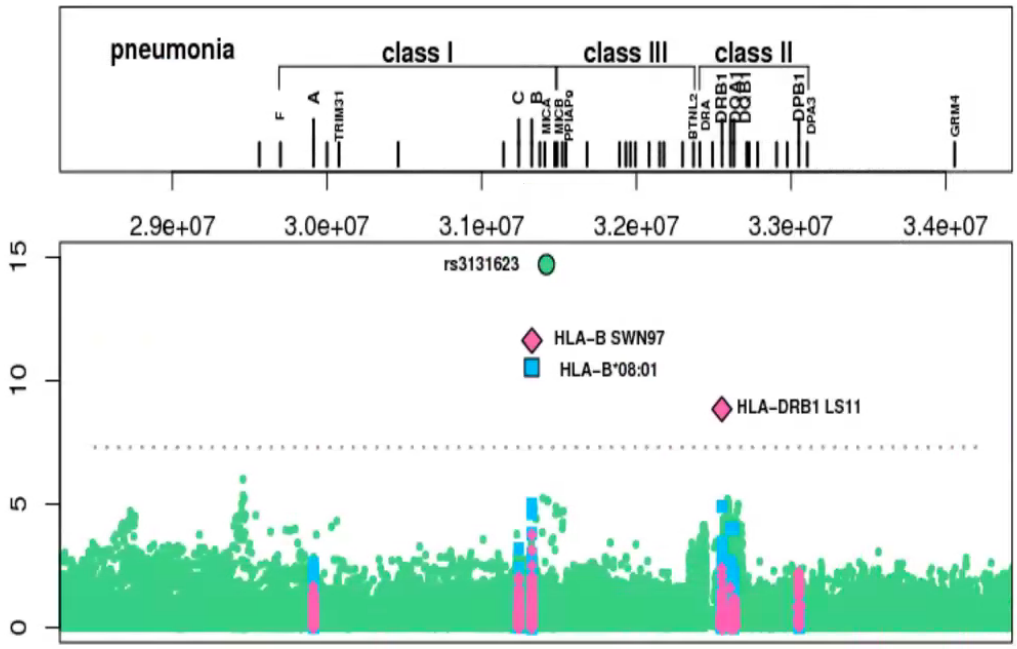

Tonsillectomy:

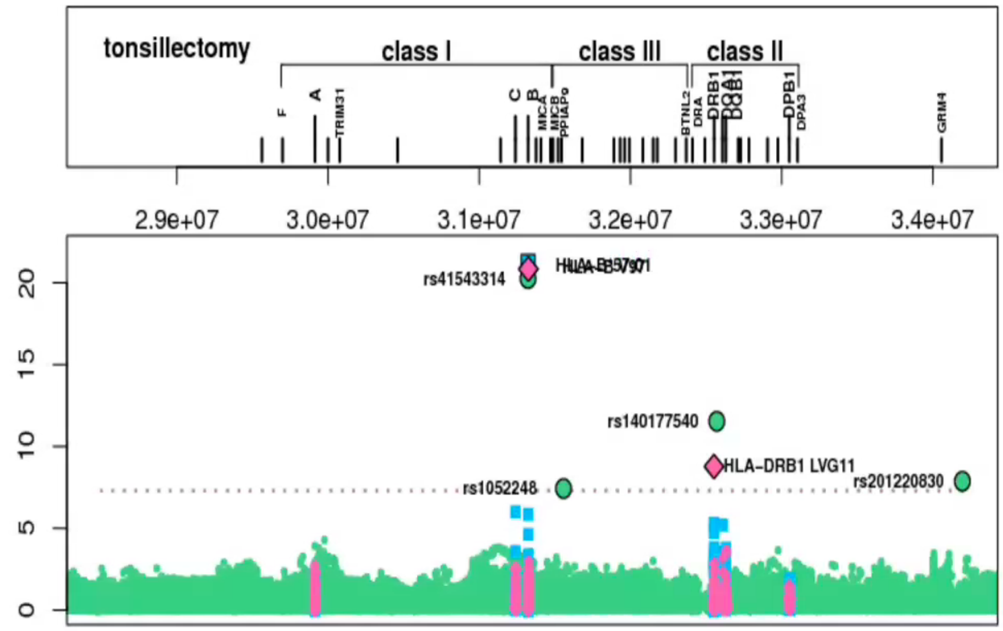

Childhood ear infection:

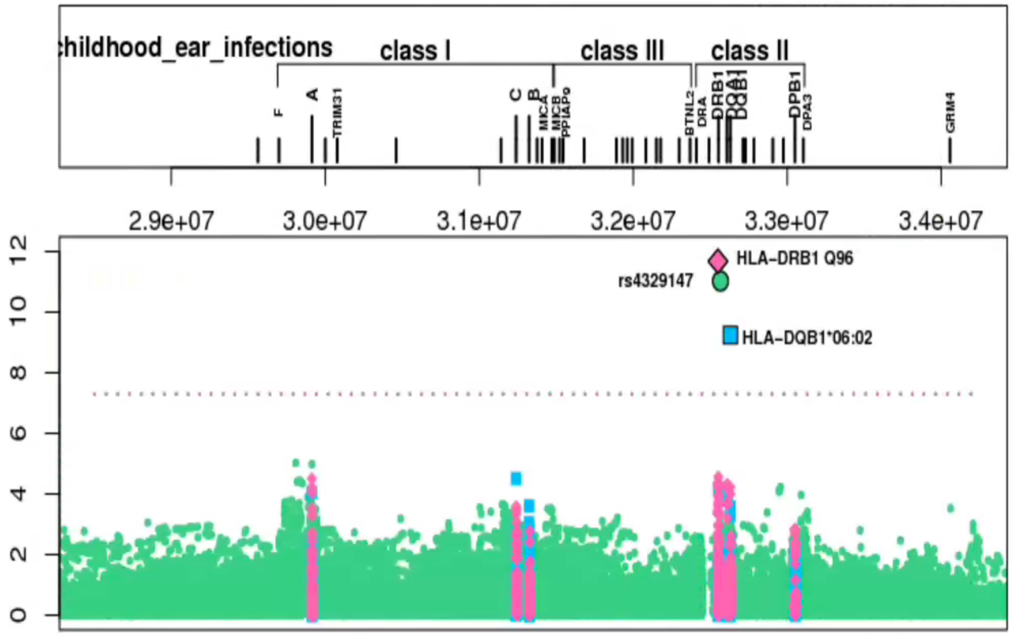

Supplementary Figure 4: Heat map showing the genetic correlations

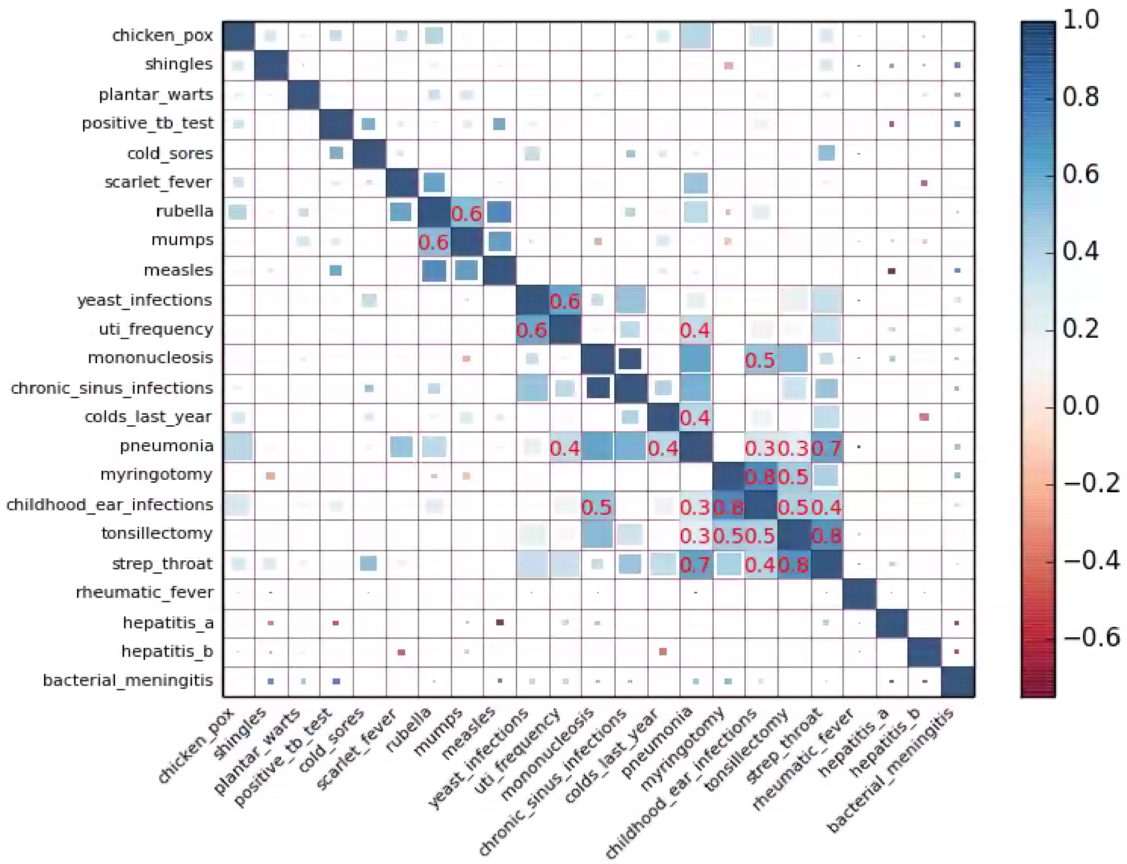

Each square [i, j] shows the estimated genetic correlation (rg) between trait i and trait j, where i indexes row and j indexes columns. Blue: positive genetic correlation; red, negative genetic correlation. Darker colors represent larger genetic correlations. Larger squares correspond to more significant P values; genetic correlations that are significantly ($P<2x10^{-4}$) different from zero after Bonferroni correction for the ~250 tests in this correlation analysis are shown as full-sized squares.

Supplementary Figure 5: Forest plots of rs601338-G (*FUT2* secretor (se) allele) and rs72646967-C (missense mutation N397H in *TBX1*)

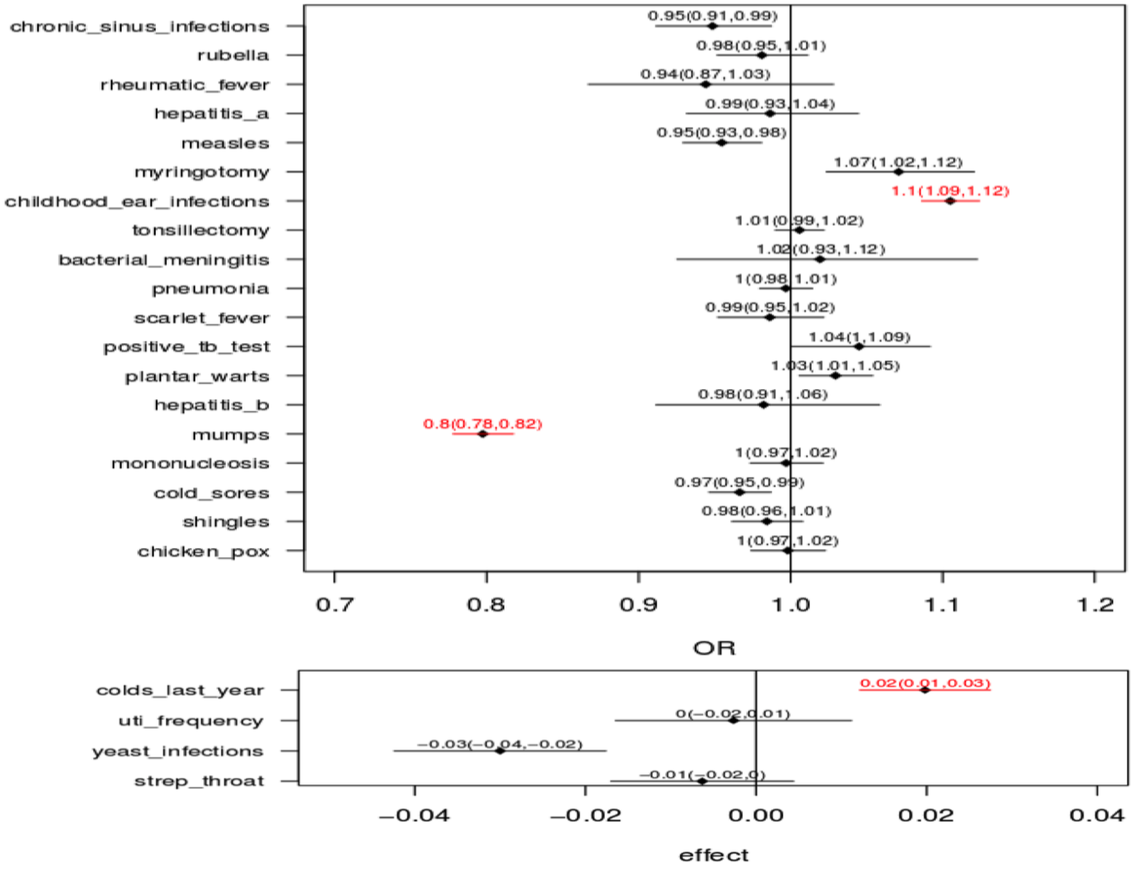

A. OR/effect were from the GWASes and shown for rs601338-G: *FUT2* secretor (se) allele

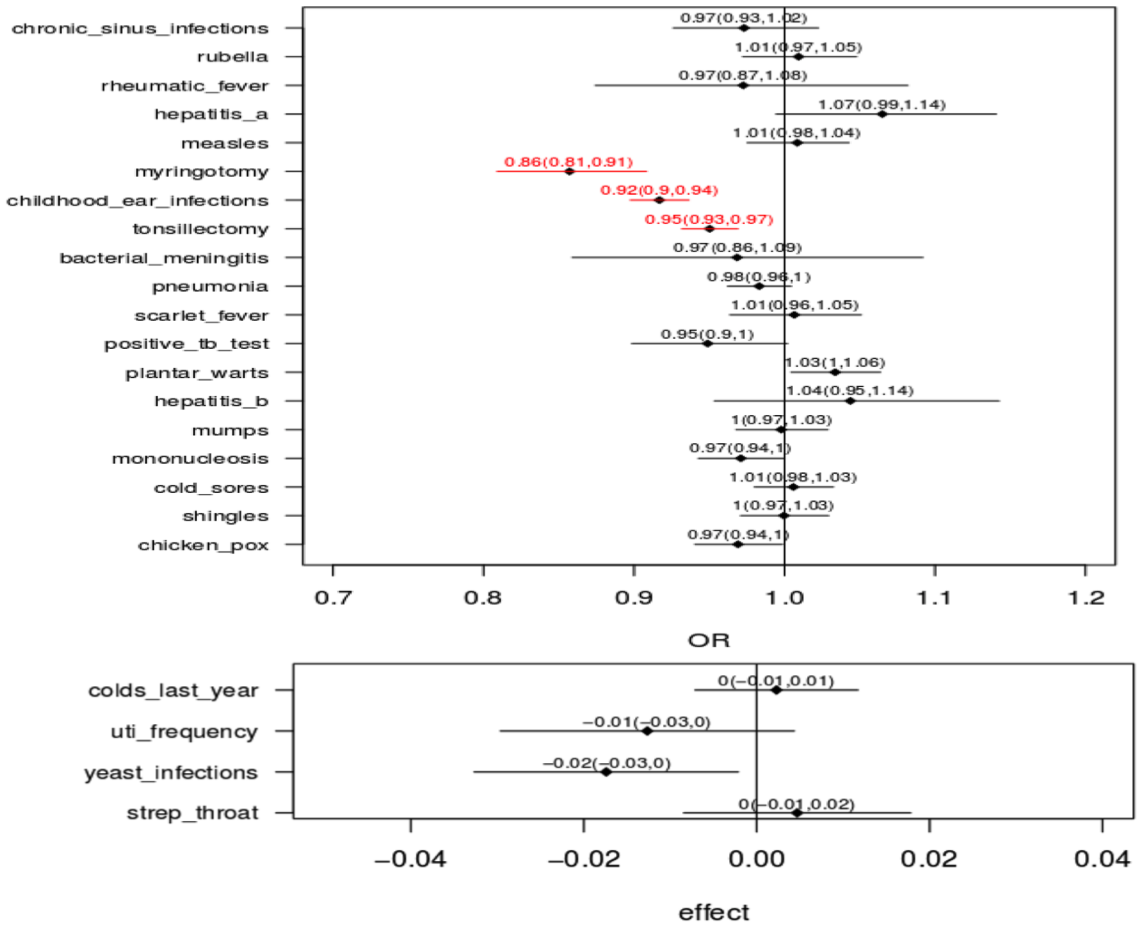

B. OR/effect were from the GWASes and shown for rs72646967-C: missense mutation (N397H) in TBX1.

Supplementary Table 1: Summary of previously reported GWAS signals

Phenotype	PMID	Description	Ancestry	Index SNP	Region	Pvalue	OR/effect	Our Pvalue	Our OR/effect
**Shingles**	25297839	GWAS from electronic medical record, 2280 shingles cases and 20k controls	European and combined ancestry	rs114864815-T/merge to rs77349273	6p21.33	1.00E-06	0.74	9.18e-22	0.81
**EBNA-1 antibody (mononucleosis)**	23326239	1367 extended family, study antibodies titer against Epstein-Barr virus nuclear antigen 1(EBNA-1), Identify two independent associations in HLA-DRB1 and HLA-DQB1 region	Mexican American	rs477515-T	6p21.32	3.00E-13	0.28 unit	3.83e-02	1.03
				rs2854275-T	6p21.32	2.00E-10	0.45 unit	4.31e-03	0.94
**Chronic hepatitis B**	23760081	Chronic hepatitis B, CHB carrier vs. normal control	Asian	rs1419881-?	6p21.33	1.00E-18	1.37	0.242	0.96
	25802187			rs3130542-A	6p21.33	9.00E-07	1.17	0.077	1.09
	21750111			rs2853953-A	6p21.33	5.00E-20	1.47	0.943	1.00
	24940741			rs652888-?	6p21.33	7.00E-13	1.38	0.986	1.00
	19349983			rs12614-T	6p21.33	1.00E-34	1.89	0.091	1.11
	22737229 / 24162738	Chronic hepatitis B, CHB carrier vs. naturally cleared HBV infection control		rs422951-G	6p21.32	5.00E-16	1.27	0.860	0.99
				rs2856718-?	6p21.32	2.00E-24	1.60	0.362	1.04
				rs9276370-T	6p21.32	2.00E-12	1.95	0.534	1.02
				rs7756516-?	6p21.32	1.00E-12	1.49	0.913	1.00
				rs7453920-?	6p21.32	1.00E-12	1.49	0.357	1.04
				rs378352-T	6p21.32	1.00E-23	1.26	0.434	1.04
				rs3077-?	6p21.32	5.00E-39	1.89	2.58e-05	1.22
				rs9277535-?	6p21.32	4.00E-40	1.89	3.66e-02	1.10
				rs9366816-?	6p21.32	1.00E-12	1.49	9.55e-01	1.00
				rs11866328-G	16p13.2	2.00E-08	1.68	4.71e-01	0.97

				rs1883832-T	20q13.12	3.00E-15	1.19	8.78e-01	1.01
				rs4821116-G	22q11.21	2.00E-12	1.22	3.20e-01	0.95
**Tuberculosis**	20694014	2237 TB cases vs.3122 controls	African	rs4331426-G	18q11.2	6.80E-09	1.19	0.952	1.00
	22306650	1329 TB cases vs. 1847 controls	African	rs2057178-A	11p13	2.63E-09	0.77	0.609	1.00
	25774636	5530 pulmonary TB cases + 5607 healthy controls	Russian	rs4733781-C	8q24.21	2.60E-11	0.84	0.973	1.00
**Bacterial meningitis**	20694013	475 +968 caess + 4703 +1376controls	European	rs1065489-T	1q31.3 (CFH)	2.20E-11	0.64	0.799	0.98
				rs426736-G	1q31.3 (CFHR3)	4.60E-13	0.63	0.815	1.02
**Chronic otitis media**	23974705	602 subjects from 143 families + 1584 subjects from 441 family, family based association test	European	rs10497394-G	2q31.1	1.52E-08	1.51	0.381	0.99
**Tonsillectomy**	27941131	1464 cases + 12019 controls	European	rs2412971	22q12.2 (HORMAD2)	1.48E-09	1.22	3.01e-32	1.10

Pvalue: the reported p-value in previously published GWAS; **OR/effect**: The effect of the list variant in previously published GWAS; **Our Pvalue**: the p-value of the list variant in our GWAS; **Our OR/effect**: the effect of the list variant in our GWAS.

Supplementary Table 2: Demographic characteristics of cohorts

		Total	Male	Female	Age (0,30]	Age (30,45]	Age (45,60]	Age (60,inf)
**Chicken pox**	control	15982	10062(62.96%)	5920(37.04%)	2920(18.27%)	4896(30.63%)	4089(25.59%)	4077(25.51%)
	case	107769	51799(48.06%)	55970(51.94%)	11758(10.91%)	28738(26.67%)	29467(27.34%)	37806(35.08%)
**Shingles**	control	118152	60967(51.60%)	57185(48.40%)	15742(13.32%)	34041(28.81%)	32043(27.12%)	36326(30.75%)
	case	16711	7456(44.62%)	9255(55.38%)	827(4.95%)	2625(15.71%)	4618(27.63%)	8641(51.71%)
**Cold sores**	control	63332	34679(54.76%)	28653(45.24%)	8807(13.91%)	19246(30.39%)	16489(26.04%)	18790(29.67%)
	case	25108	12063(48.04%)	13045(51.96%)	2095(8.34%)	6050(24.10%)	7124(28.37%)	9839(39.19%)
**Mononucleosis**	control	68446	33989(49.66%)	34457(50.34%)	7971(11.65%)	16975(24.80%)	18790(27.45%)	24710(36.10%)

	case	17457	6898(39.51%)	10559(60.49%)	1902(10.90%)	4808(27.54%)	5585(31.99%)	5162(29.57%)
**Mumps**	control	54153	29229(53.97%)	24924(46.03%)	10558(19.50%)	22452(41.46%)	12727(23.50%)	8416(15.54%)
	case	31227	15672(50.19%)	15555(49.81%)	189(0.61%)	1854(5.94%)	9762(31.26%)	19422(62.20%)
**Hepatitis B**	control	218180	114913(52.67%)	103267(47.33%)	30730(14.08%)	61898(28.37%)	60413(27.69%)	65139(29.86%)
	case	1425	893(62.67%)	532(37.33%)	18(1.26%)	162(11.37%)	531(37.26%)	714(50.11%)
**Plantar warts**	control	37451	18378(49.07%)	19073(50.93%)	4301(11.48%)	9487(25.33%)	10257(27.39%)	13406(35.80%)
	case	24994	10908(43.64%)	14086(56.36%)	2901(11.61%)	7009(28.04%)	7438(29.76%)	7646(30.59%)
**Positive TB test**	control	84290	44932(53.31%)	39358(46.69%)	10810(12.82%)	24521(29.09%)	22460(26.65%)	26499(31.44%)
	case	4426	2080(47.00%)	2346(53.00%)	275(6.21%)	890(20.11%)	1044(23.59%)	2217(50.09%)
**Strep throat**	never	22017	12124(55.07%)	9893(44.93%)	2971(13.49%)	5859(26.61%)	5807(26.38%)	7380(33.52%)
	1-2	26238	12951(49.36%)	13287(50.64%)	3267(12.45%)	6753(25.74%)	7569(28.85%)	8649(32.96%)
	3-5	14435	6362(44.07%)	8073(55.93%)	2059(14.26%)	4427(30.67%)	4087(28.31%)	3862(26.75%)
	5+	11814	4262(36.08%)	7552(63.92%)	1575(13.33%)	4208(35.62%)	3448(29.19%)	2583(21.86%)
**Scarlet fever**	control	113837	59250(52.05%)	54587(47.95%)	14716(12.93%)	32849(28.86%)	30560(26.85%)	35712(31.37%)
	case	6812	2771(40.68%)	4041(59.32%)	481(7.06%)	1449(21.27%)	1758(25.81%)	3124(45.86%)
**Pneumonia**	control	90039	48301(53.64%)	41738(46.36%)	12358(13.73%)	26193(29.09%)	23951(26.60%)	27537(30.58%)
	case	40600	17801(43.84%)	22799(56.16%)	3368(8.30%)	9042(22.27%)	11736(28.91%)	16454(40.53%)
**Bacterial meningitis**	control	82778	38953(47.06%)	43825(52.94%)	8861(10.70%)	19917(24.06%)	23810(28.76%)	30190(36.47%)
	case	842	403(47.86%)	439(52.14%)	88(10.45%)	218(25.89%)	268(31.83%)	268(31.83%)
**Yeast infections**	0	10235	0(0.00%)	10235(100.00%)	2753(26.90%)	2707(26.45%)	2037(19.90%)	2738(26.75%)
	1-2	16636	0(0.00%)	16636(100.00%)	2395(14.40%)	4490(26.99%)	4605(27.68%)	5146(30.93%)
	3-4	14057	0(0.00%)	14057(100.00%)	1318(9.38%)	3529(25.10%)	4573(32.53%)	4637(32.99%)
	5+	21525	0(0.00%)	21525(100.00%)	1300(6.04%)	5189(24.11%)	7872(36.57%)	7164(33.28%)
**UTI frequency**	never	33478	25563(76.36%)	7915(23.64%)	5651(16.88%)	11189(33.42%)	8251(24.65%)	8387(25.05%)
	once	10168	4351(42.79%)	5817(57.21%)	1173(11.54%)	2822(27.75%)	2910(28.62%)	3263(32.09%)
	twice	7036	1738(24.70%)	5298(75.30%)	653(9.28%)	1672(23.76%)	2145(30.49%)	2566(36.47%)
	thrice	5090	795(15.62%)	4295(84.38%)	433(8.51%)	1187(23.32%)	1596(31.36%)	1874(36.82%)

	four_or_more	12706	827(6.51%)	11879(93.49%)	967(7.61%)	2903(22.85%)	3939(31.00%)	4897(38.54%)
**Tonsillectomy**	control	113323	60724(53.58%)	52599(46.42%)	19679(17.37%)	40698(35.91%)	29860(26.35%)	23086(20.37%)
	case	60098	29487(49.06%)	30611(50.94%)	3212(5.34%)	8715(14.50%)	17103(28.46%)	31068(51.70%)
**Childhood ear infections**	control	74874	38359(51.23%)	36515(48.77%)	9778(13.06%)	20720(27.67%)	21043(28.10%)	23333(31.16%)
	case	46936	22914(48.82%)	24022(51.18%)	7829(16.68%)	14299(30.46%)	12580(26.80%)	12228(26.05%)
**Myringotomy**	control	85089	45081(52.98%)	40008(47.02%)	10266(12.07%)	23831(28.01%)	22809(26.81%)	28183(33.12%)
	case	4138	2261(54.64%)	1877(45.36%)	965(23.32%)	1874(45.29%)	788(19.04%)	511(12.35%)
**Measles**	control	47279	25708(54.38%)	21571(45.62%)	10377(21.95%)	21652(45.80%)	11592(24.52%)	3658(7.74%)
	case	38219	19203(50.24%)	19016(49.76%)	253(0.66%)	2310(6.04%)	10777(28.20%)	24879(65.10%)
**Hepatitis A**	control	217137	114475(52.72%)	102662(47.28%)	30683(14.13%)	61685(28.41%)	60153(27.70%)	64616(29.76%)
	case	2442	1308(53.56%)	1134(46.44%)	58(2.38%)	368(15.07%)	777(31.82%)	1239(50.74%)
**Rheumatic fever**	control	88076	46861(53.21%)	41215(46.79%)	11157(12.67%)	25562(29.02%)	23374(26.54%)	27983(31.77%)
	case	1115	472(42.33%)	643(57.67%)	12(1.08%)	71(6.37%)	258(23.14%)	774(69.42%)
**# of colds last year**	none	15720	6930(44.08%)	8790(55.92%)	938(5.97%)	2467(15.69%)	4473(28.45%)	7842(49.89%)
	1-2	35668	17050(47.80%)	18618(52.20%)	3823(10.72%)	9266(25.98%)	10515(29.48%)	12064(33.82%)
	3-4	6709	3009(44.85%)	3700(55.15%)	1185(17.66%)	2487(37.07%)	1706(25.43%)	1331(19.84%)
	egt5	1449	582(40.17%)	867(59.83%)	290(20.01%)	616(42.51%)	345(23.81%)	198(13.66%)
**Rubella**	control	71597	39924(55.76%)	31673(44.24%)	10867(15.18%)	23927(33.42%)	19239(26.87%)	17564(24.53%)
	case	12000	4350(36.25%)	7650(63.75%)	130(1.08%)	925(7.71%)	2999(24.99%)	7946(66.22%)
**Chronic sinus infections**	control	79622	37742(47.40%)	41880(52.60%)	9026(11.34%)	19687(24.73%)	22605(28.39%)	28304(35.55%)
	case	5291	2405(45.45%)	2886(54.55%)	305(5.76%)	1083(20.47%)	1753(33.13%)	2150(40.64%)

Supplementary Table 3: The annotation for genes identified in mumps GWAS

Genes	Canonical pathways	NCBI Gene function summary	Index SNP annotation

19q13.3<br>FUT2	Glycosphingolipid biosynthesis	Fucosyltransferase 2, this gene is a Golgi stack membrane protein that is involved in the creation of a precursor of the H antigen, which is required for the final step in soluble A and B antigen synthesis pathway. This gene encodes the galactoside 2-L-fucosyltransferase enzyme.	The risk allele rs516316-C is in high LD with rs601338-A (r^2=1, P=9.94e-72 in our GWAS) and rs602662-A (r2=0.88,p=3.10e-67 in our GWAS). rs601338-A is a nonsense variant in FUT2 that encodes the "non-secretor" (se) allele. rs602662-A is a missense variant in FUT2.
14q32.2<br>BDKRB2	Calcium signaling pathway, Neuroactive ligand-receptor interaction, Complement and coagulation cascades, Regulation of actin cytoskeleton	Bradykinin receptor B2, the encoded bradykinin receptor associates with G proteins that stimulate a phosphatidylinositol-calcium second messenger system.	None of the variants within 500kb and in moderate LD (r2>0.6) with the index SNP rs11160318-G were coding, nor were they reported as eQTLs. However, rs11160318 falls within some promoter and enhancer histone marks, and may also cause changes in regulatory motifs.
11q24.2<br>ST3GAL4	Glycosphingolipid biosynthesis	ST3 beta-galactoside alpha-2,3-sialyltransferase 4, this gene encodes a member of the glycosyltransferase 29 family, a group of enzymes involved in protein glycosylation. The encoded protein is targeted to Golgi membranes but may be proteolytically processed and secreted. The gene product may also be involved in the increased expression of sialyl Lewis X antigen seen in inflammatory responses.	None of the variants within 500kb and in moderate LD (r2>0.6) with the index SNP rs3862630 were coding, nor were they reported as eQTLs. However, rs3862630 falls within strong enhancers defined in epidermal cells. It located in the region bound by AP2GAMMA, which is activating enhancer-binding protein that interact with viral or cellular elements to regulate transcription of selected genes.
9q34.2<br>ABO	ABO blood group	This gene encodes proteins related to the first discovered blood group system, ABO. Which allele is present in an individual determines the blood group.	The index SNP rs8176643 were reported as eQTLs for ABO (eQTL p-value=4.16e-8). It also falls into active promoters and enhancers defined in various types of cells.

Supplementary Table 4: The annotation for genes identified in tonsillectomy GWAS

Genes	Canonical pathways	NCBI Gene function summary	Index SNP annotation

12p13.31<br>LTBR	Intestinal immune network for IgA production	Lymphotoxin beta receptor, it encodes a member of the tumor necrosis factor receptor superfamily. The encoded protein plays a role in signaling during the development of lymphoid and other organs, lipid metabolism, immune response and programmed cell death	The index SNP rs10849448 is an eQTL for LTBR (eQTL p-value=3.5e-34). It falls in some active promoters and enhancers defined in lymphoblastoid and epidermal cells.
22q12.2<br>MTMR3	Phospholipid Metabolism, Synthesis of PIPs at the plasma membrane, PI Metabolism	Myotubularin related protein 3, the encoded protein binds to phosphoinositide lipids through the PH-GRAM domain, and can hydrolyze phosphatidylinositol(3)-phosphate and phosphatidylinositol(3,5)-biphosphate in vitro.	The index risk allele rs201112509-TA is in LD with multiple eQTL SNPs. rs131291 (r2=0.6 with rs201112509, eQTL p-value=4.4e-4) is an eQTL for XBP1. rs718772 (r2=0.74 with rs201112509, eQTL p-value=3.8e-4) is an eQTL for UCRC. rs9625933 (r2=0.74 with rs201112509, eQTL p-value=4.5e-5) is an eQTL for OSM.
17p11.2<br>*TNFRSF13B*	Intestinal immune network for IgA production, TACI and BCMA stimulation of B cell immune responses	Tumor necrosis factor receptor superfamily, member 13B. The gene is within the Smith-Magenis syndrome region on chr17. Its encoded protein induces activation of the transcription factors NFAT, AP1, and NF-KAPPA-B and plays important role in humoral immunity by interacting with a TNF ligand.	rs34557412 is a missense variant in TNFRSF13B.
13q33.3<br>TNFSF13B	Intestinal immune network for IgA production, TACI and BCMA stimulation of B cell immune responses	Tumor necrosis factor (ligand) superfamily, member 13b. The encoded protein is a cytokine, which is a ligand for receptors TNFRSF13B/TACI, TNFRSF17/BCMA and TNFRSF13C/BAFFR. It plays roles in B cell activation, proliferation and differentiation.	rs200748895 is in 3'UTR and falls within enhancers in CD4+ cells.
7p12.3<br>IGFBP3	p53 signaling pathway, Hypoxia and p53 in the Cardiovascular system, Direct p53 effectors	Insulin-like growth factor binding protein 3. It encoded protein forms complex with insulin-like growth factor acid-labile subunit (IGFALS) and insulin-like growth factor (IGF). In the complex form, it circulates in the plasma, prolonging the half-life of IGFs and altering their interaction with cell surface receptors.	Index SNP rs80077929 is intergenic and upstream of IGFBP3.

4q24<br>NFKB1	TACI and BCMA stimulation of B cell immune responses, Ceramide signaling pathway	Nuclear factor of kappa light polypeptide gene enhancer in B-cells 1. It is a transcription regulator that is activated by various intra- and extra-cellular stimuli such as cytokines, oxidant-free radicals, ultraviolet irradiation, and bacterial or viral products. Inappropriate activation of NFKB has been associated with a number of inflammatory diseases while persistent inhibition of NFKB leads to inappropriate immune cell development or delayed cell growth.	The index risk allele rs230523-T is in high LD with SNPs in 5'UTR of NFKB and it falls within enhancers defined in CD4+ cells.
7p15.2<br>*HOTAIRM1, HOXA2*	Transcription factors	Homeobox A gene cluster. The gene expression in this region is spatially and temporally regulated during embryonic development. HOXA encodes a DNA-binding transcription factor that regulates gene expression, morphogenesis, and differentiation.	The index risk allele rs6668-T falls within enhancers and promoters that are defined in epidermal cells. CTCF protein binds to the SNP region.
14q21.1<br>FOXA1	FOXA1,<br>FOXA2 and FOXA3 transcription factor networks, Direct p53 effectors	Forkhead box A1, encodes DNA-binding proteins, which are transcriptional activators for liver-specific transcripts, and also interact with chromatin. Similar family members also play roles in the regulation of metabolism and in the differentiation of the pancreas and liver.	The index risk allele rs148131694-T is in LD with missense variant rs7144658-C (r2=0.55, A83T in FOXA1, P=1.58e-12 in our GWAS)
4q24<br>TET2		tet methylcytosine dioxygenase 2, this gene product catalyzes the conversion of methylcytosine to 5-hydroxymethycytosine.The encoded protein is involved in myelopoiesis, and defects in this gene have been associated with several myeloprofiferative disorders.	The index risk allele rs1391439-G is an eQTL for NPNT, nephronectin (eQTL p-value=7.2e-14) in lymphoblastoid.
2p14<br>SPRED2	Jak-STAT signaling pathway, Signaling events mediated by Stem cell factor receptor (c-Kit)	sprouty-related, EVH1 domain containing 2. This gene is a member of the Sprouty/SPRED family of proteins that regulate growth factor-induced activation of the MAP kinase cascade.	The index risk allele rs201473667-A is in high LD (r2>0.8) with SNP in enhancers that are defined in lymphoctype and epidermal keratinocyte.

20q13.12 CD40	Intestinal immune network for IgA production	This gene is a member of the TNF-receptor superfamily. The gene product is a receptor on antigen-presenting cells of the immune system and is essential for mediating a broad variety of immune and inflammatory responses including T cell-dependent immunoglobulin class switching, memory B cell development, and germinal center formation.	The index risk allele rs6032664-A is in high LD (r2>0.98) with SNP in 5'UTR of CD40. They fall in enhancers and promoters.
7p12.2 IKZF1	Calcineurin-regulated NFAT-dependent transcription in lymphocytes	IKAROS family zinc finger 1, is a transcription factor that belongs to the family of zinc-finger DNA-binding proteins associated with chromatin remodeling. This gene expression is restricted to the fetal and adult hemolymphopoietic system, and it functions as a regulator of lymphocyte differentiation. Overexpression of some dominant-negative isoforms has been associated with B-cell malignancies, such as acute lymphoblastic leukemia (ALL).	The index risk allele rs876037-T falls in strong enhancers defined in lymphoblastoid. And also overlap with some promoter histone marks.
13q21.33 KLHL1		Kelch-like family member 1, this gene product belongs to a family of actin-organizing proteins related to Drosophila Kelch.	Not known
7p22.2 GNA12	Vascular smooth muscle contraction, Sphingosine 1-phosphate (S1P) pathway, Genes involved in Hemostasis, Genes involved in Platelet activation, signaling and aggregation	Guanine nucleotide binding protein (G protein) alpha 12, it is involved as modulators or transducers in various transmembrane signaling systems. GO annotations related to this gene include GTP binding and GTPase activity.	The index risk allele rs2644312-G is in high LD with rs1182188 (r2=0.9), which is an eQTL for GNA12 (eQTL p-value=2.8e-23). It is also in LD with rs2644296 (r2=0.57), which is an eQTL for FTSJ2 (eQTL p-value=9.3e-6).
3q21.2 SLC12A8		Solute carrier family 12, member 8, this gene is thought to be a candidate for psoriasis susceptibility.	The index risk allele rs1980080-C falls in strong enhancers defined in lymphoblastoid.
16p11. 2 SBK1		SH3 domain binding kinase 1. Some study suggests a role for SBK in signal-transduction pathways related to the control of brain development.	The index risk allele rs141876325-CACCT is a small insertion. It is in LD with rs2650492 (r2=0.88) in 3'UTR of SBK1. This 3'UTR region is bound by transcription factor CTCF. Both SNPs falls in some

			enhancers.
1q41<br>DUSP10---[]---HHIPL2		DUSP10: dual specificity phosphatase 10, this gene product negatively regulates members of the MAP kinase superfamily, which is associated with cellular proliferation and differentiation. HHIPL2: HHIP-like 2. Unknown role.	The index risk allele rs12126292-G falls in strong enhancers found in epithelial cells and lymphoblastoid. This region is also bound by NFKB, EBF1 and BCL11A.
4p15.2<br>SEPSECS	Aminoacyl-tRNA biosynthesis	The amino acid selenocysteine is the only amino acid that does not have its own tRNA synthetase. Instead, it is synthesized on its cognate tRNA in a three-step process.	Index risk allele rs10939037-A is in LD with rs13139513 (r2=0.63), which is an eQTL for ANAPC4.
12q24.12<br>SH2B3	Signaling events mediated by Stem cell factor receptor, Genes involved in Regulation of KIT signaling, Genes involved in Factors involved in megakaryocyte development and platelet production, Genes involved in Hemostasis	SH2B adaptor protein 3, this gene product is a member of the SH2B adaptor family of proteins, which are involved in a range of signaling activities by growth factor and cytokine receptors. It is a key negative regulator of cytokine signaling and plays a critical role in hematopoiesis.	Index risk allele rs3184504-T is a missense variant in SH2B3 (W262R)
4q21.1<br>CXCL13	Cytokine-cytokine receptor interaction, Chemokine signaling pathway, CXCR3-mediated signaling events	Chemokine (C-X-C motif) ligand 13, encodes B lymphocyte chemattractant, which is an antimicrobial peptide and CXC chemokine strongly expressed in the follicles of the spleen, lymph nodes and Peyer's patches. It preferentially promotes the migration of B lymphocytes.	No informative annotation. Index SNP is intronic to CXCL13.
7q31.2<br>MDFIC,<br>TFEC	MDFIC : Regulation of nuclear beta catenin signaling and target gene transcription.<br>TFEC: C-MYB transcription factor network	MDFIC: MyoD family inhibitor domain containing, which is involved in transcription regulation of viral genome expression.<br>TFEC: transcription factor EC, it play roles in multiple cellular processes including survival, growth and differentiation.	No informative annotation. Index rs2023703 is intergenic

19p13.2 ADAMTS10, ACTL9		ADAM metallopeptidase with thrombospondin type 1 motif, 10, it belongs to the ADAMTS (a disintegrin and metalloproteinase domain with thrombospondin type-1 motifs) family. This family has been demonstrated to have important roles in growth and in skin, lens, and heart development. Actin-like 9, actin proteins have cytoskeletal functions, are important for the maintenance of epithelial morphology and cell migration.	No informative annotation for index SNP rs2918308-A
17p13.3 RAP1GAP2	Genes involved in Rap1 signaling, Genes involved in Immune System, Genes involved in Adaptive Immune System	RAP1 GTPase activating protein 2, it encodes a GTPase-activating protein that activates the small guanine-nucleotide-binding protein Rap1 in platelets. The protein interacts with synaptotagmin-like protein 1 and Rab27 and regulates secretion of dense granules from platelets at sites of endothelial damage.	Index SNP rs67968065-AT, no informative annotation, but is falls in several enhancers defined in CD34 cells.
21q22.11 ITSN1	Many signaling pathways (such as EPHB forward signaling, Genes involved in Signaling by Rho GTPases, Genes involved in Signaling by NGF etc.)	Intersectin 1, this gene product is a cytoplasmic membrane-associated protein that indirectly coordinates endocytic membrane traffic with the actin assembly machinery. The encode protein may regulate the formation of clathrin-coated vesicles and could be involved in synaptic vesicle recycling.	The index SNP rs200746495 is in moderate LD with rs9978525 (r2=0.59), which is an eQTL for IFNGR2 (eQTL p=3.4e-9) and TMEM50B (eQTL p=9.6e-8).
2q33.3 ADAM23		ADAM metallopeptidase domain 23, it encodes a member of the ADAM (a disintegrin and metalloprotease domain) family. This family has been implicated in a variety of biological processes involving fertilization, muscle development, and neurogenesis.	Index SNP rs1448903 falls within promoters and enhancers. CTCF bound to this SNP region.
1p36.23 RERE		Arginine-glutamic acid dipeptide (RE) repeats, its encoded protein co-localizes with a transcription factor and its overexpression triggers apoptosis. It is thought to function as a transcriptional co-repressor during embryonic development.	Index SNP rs12068123-G, no informative annotation, but it is in moderate LD (r2=0.59) with rs1318218, which is an eQTL for RERE in monocyte. There is another independent bue less significant signal at RERE (indexed by rs56025131, p=8e-7)

11p15.4<br>ST5		Suppression of tumorigenicity 5, it encode a protein preferentially binds to the SH3 domain of c-Abl kinase, and acts as a regulator of MAPK1/ERK2 kinase.	Index SNP rs11042055 is in high LD (r2=0.96) with missense variant rs3794153 in ST5. It is also in high LD (r2=0.90) with rs11042067, which is an eQTL for ST5 in lymphblastoid (eqtl pvalue=1.2e-7).
20q13.33<br>SAMD10		Sterile alpha motif domain containing 10, exact role is not clear	Index SNP rs41278232 is in 3'UTR of SAMD10 and falls in the region that bound by FOXA1
9q34.2<br>ABO	Glycosphingolipid biosynthesis	ABO blood group, this gene encodes proteins related to the first discovered blood group system, ABO.	Index SNP rs635634-T falls in strong enhancers in leukemia.
17q11.2<br>FOXN1	Genes related to Wnt-mediated signal transduction	Forkhead box N1 or "winged-helix" transcription factors, this gene is involved in epidermis development, keratinocyte differentiation, and thymus development etc. mutations in this gene in mice and rats lead to hairlessness and athymia. Mutations in this gene have been correlated with T-cell immunodeficiency, skin disorder congenital alopecia and nail dystrophy.	Index SNP rs62066768-A is in high LD (r2=0.97) with missense variant rs2071587-T in FOXN1.
6q23.3<br>TNFAIP3	NOD-like receptor signaling pathway, CD40L Signaling Pathway, NF-kB Signaling Pathway, TNFR2 Signaling Pathway, Tumor Necrosis Factor Pathway, Canonical NF-kappaB pathway, Genes involved in Innate Immune System	Tumor necrosis factor, alpha-induced protein 3, the expression of this gene is rapidly induced by the tumor necrosis factor (TNF). This gene product has been shown to inhibit NF-kappa B activation and inhibit TNF-mediated apoptosis. The protein, which has both ubiquitin ligase and deubiquintinase activities, is involved in the cytokine-mediated immune and inflammatory responses.	Index SNP rs11757201-C is intergenic, no informative annotation, but it is in complete LD with rs6927172, which falls in many enhancers and promoter sequences. And the SNP position may be bound by many proteins such as STAT1, MAFK and STAT3 etc.
16p11.2<br>MAPK3	Ceramide signaling pathway, Glucocorticoid receptor regulatory network	Mitogen-activated protein kinase 3, also known as extracellular signal-regulated kinases (ERKs). The encoded protein acts in a signaling cascade that regulates various cellular processes such as proliferation, differentiation and cell cycle progression in response to extracellular signals.	Index SNP rs12931792 is intergenic, upstream of MAPK3. It is in LD with multiple SNPs are reported as eQTLs. rs4787495 (r2=0.693, eQTL p-value=1.1e-10, GWAS p=2.26e-5) is an eQTL for GDPD3. rs4471699 (r2=0.565, eQTL p=7.9e-19, GWAS p=5.95e-6) is an eQTL for MAPK3.
7q31.2<br>WNT2	Wnt signaling pathway, Hedgehog signaling pathway, Melanogenesis, cancer pathway,	Wingless-type MMTV integration site family member 2. WNT gene family consists of structurally related genes, which encode secreted	Index SNP rs200608253-TC is in 3'UTR of WNT2 and falls in the sequence that bound by MAFK. MAFK encode transcription factor MafK that is a member of

	Basal cell carcinoma	signaling proteins. The encoded protein has been implicated in ontogenesis and developmental processes, such as cell fate patterning during embryogenesis.	the activator protein-1 superfamily of basic leucine zipper proteins.
22q11.21 TBX1		T-box 1, encode transcription factors involved in the regulation of developmental processes. Deletions around this gene region of 22q11.2 have been associated with DiGeorge syndrome.	Index SNP rs41298830-A is in high LD (r2=0.9) with rs72646967 (p=3.40e-7, risk allele), a missense variant N397H in TBX1.

Supplementary Table 5: The annotation for genes identified in childhood ear infection GWAS

Genes	Canonical pathways	NCBI Gene function summary	Index SNP annotation
19q13.33 FUT2	Glycosphingolipid biosynthesis	Fucosyltransferase 2, this gene is a Golgi stack membrane protein that is involved in the creation of a precursor of the H antigen, which is required for the final step in soluble A and B antigen synthesis pathway. This gene encodes the galactoside 2-L-fucosyltransferase enzyme.	The index risk allele rs681343-C is in high LD with rs601338-G (r2=1), nonsense variant, and with rs602662-G (r2=0.88), a missense variant in FUT2.
22q11.21 TBX1		T-box 1, encode transcription factors involved in the regulation of developmental processes. Deletions around this gene region of 22q11.2 have been associated with DiGeorge syndrome.	The index risk allele rs1978060-G is in LD with missense mutation rs72646967-A (r2=0.44). It falls in some strong enhancers and promoters.

10p12.1<br>RAB18, MKX		RAB18, member RAS oncogene family, this gene product is a member of a family of Ras-related small GTPases that regulate membraine trafficking in organelles and transport vesicles. Knockdown studies in zebrafish suggest that it may have role in eye and brain develeopment.<br>MKX, mohawk homeobox, this gene product is an IRX family-related homeobox transcription factor that may play a role in cell adhesion. Studies in mice suggest that it may be a regulator of tendon development.	The index SNP rs2808290-C is interginic, and may falls in some enhancer sequences.
15q26.2<br>SPATA8,<br>LINC00923		SPATA8, spermatogenesis associated 8	rs7174062-G, not clear
9q34.2 ABO	Glycosphingolipid biosynthesis - lacto and neolacto series,	ABO blood group, this gene encodes proteins related to the first discovered blood group system, ABO.	index variant 9:136149095:CA_C falls in strong enhancer defined in leukemia.
2p16.1<br>EFEMP1		EGF containing fibulin-like extracellular matrix protein 1, this gene product if a member of the fibulin family of extracellular matrix glycoproteins. It contains tandemly repeated epidermal growth factor-like repeats followed by a C-terminus fibulin-type domain. The gene is upregulated in malignant gliomas and may play roles in the aggressive nature of tumors.	The risk allele rs1802575-G is in 3'UTR of EFEMP1, it falls in strong enhancer regions defined in epithelial cells and muscle myoblasts.
2p24.1<br>NT5C1B-<br>RDH14,OSR1		Exact role is not clear	The risk allele rs5829676-G is in high LD (r2>0.9) with some enhancer kistone marks defined in epithelial cells.

11q13.3 FGF3	MAPK signaling pathway,Regulation of actin cytoskeleton,Melanoma,Genes involved in Negative regulation of FGFR signaling,Genes involved in Insulin receptor signalling cascade,Genes involved in FRS2-mediated cascade,Genes involved in PI-3K cascade	FGF3: fibroblast growth factor 3,it is a member of fibroblast growth factor (FGF) family. FGF family members involved in a varitety of biological processes including embryonic development, cell growth, morphogenesis, tissure repair, tumor growth and invasion. Studies of the similar genes in mouse and chicken suggested the role in inner ear formation.	rs72931768-G is intergenic, but falls in some enhancer and promoter sequences. It is also in LD with rs12418731 (r2=0.84) which falls in enhancers as well and is also found to be bound by MAFK.
7q11.22 AUTS2		Autism susceptibility candidate 2, this gene has been implicated in neurodevelopment and as a candidate gene for numerous neurological disorders, including autism spectrum disorders, intellectual disability, and developmental delay.	rs35213789-C is in LD (r2>0.6) with a lot of regions overlapped with enhancer histone marks and is bound by proteins such as HOXA1, HOXA2, GATA3,
7q22.3 CDHR3		Cadherin-related family member 3. The biological function of CDHR3 is unknown. It may involved in homologous cell adhesion, epithelial polarity and cell-cell interaction and differencitation.	The risk alele rs114947103-C falls in strong enhancer defined in leukemia. It also in almost complete LD with rs6967330-A(r2=0.97), which is a missense variant in CDHR3 (C529Y).
8q22.2 NIPAL2--[]--KCNS2	Genes involved in Neuronal System,Genes involved in Voltage gated Potassium channels	NIPAL2: NIPA-like domain containing 2. KCNS2: potassium voltage-gated channel, delayed-rectifier, subfamily S, memeber2.The biological function of them is unknown.	rs13281988-C it falls in regions overlapped with some enhancers defined in leukemia and lung fibroblasts.
3p21.31 BSN		Bassoon presynaptic cytomatrix protein, this gene product is thought to be a scaffolding protein involved in organizing the presynaptic cytoskeleton. The gene is expressed primarily in neurons in the brain.	rs67035515-C in high LD with rs11130217 (r2=0.98), which is eQTL for AMT (eqtl pvalue=2.5e-4), in high LD with rs1996663 (r2=0.97) which is eQTL for GMPPB (eqtl Pvalue is 2.7e-6). Is in LD with rs11130208 (r2=0.84) which is an eqtl for RHOA (eqtl pvalue=2e-5)

6q26 PLG	Neuroactive ligand-receptor interaction,Complement and coagulation cascades,Fibrinolysis Pathway,Platelet Amyloid Precursor Protein Pathway,Syndecan-4-mediated signaling events,Genes involved in Degradation of the extracellular matrix etc.	Plasminogen, this gene product is a secreted blood zymogen that is activated by proteolysis and converted to plasmin and angiostatin.Plasmin dissolves fibrin in blood clots and is an important protease in many other cellular processes while angiostatin inhibits angiogenesis. Mutations in the PLG gene can cause congenital plasminogen deficiency, which results in inflamed growths on the mucous membranes.	rs73015965-G is a missense variant in PLG.

Supplementary Table 6: The genetic correlation from LD score regression

Phenotype 1	Phenotype 2	rg	rg_se	rg_p
chicken_pox	shingles	0.3478	0.1695	4.02E-02
chicken_pox	cold_sores	0.0831	0.2147	6.99E-01
chicken_pox	mononucleosis	0.0998	0.2199	6.50E-01
chicken_pox	mumps	0.1938	0.1407	1.69E-01
chicken_pox	hepatitis_b	-0.2219	0.2994	4.59E-01
chicken_pox	plantar_warts	0.2874	0.2053	1.62E-01
chicken_pox	positive_tb_test	0.3869	0.1986	5.14E-02
chicken_pox	strep_throat	0.323	0.1412	2.22E-02
chicken_pox	scarlet_fever	0.3632	0.1866	5.16E-02
chicken_pox	pneumonia	0.4306	0.1227	5.00E-04
chicken_pox	bacterial_meningitis	0.1257	0.3423	7.14E-01
chicken_pox	yeast_infections	-0.0134	0.109	9.02E-01
chicken_pox	uti_frequency	0.1408	0.113	2.13E-01
chicken_pox	tonsillectomy	0.0791	0.0867	3.62E-01
chicken_pox	childhood_ear_infections	0.3129	0.0994	1.70E-03
chicken_pox	myringotomy	0.074	0.1585	6.41E-01
chicken_pox	measles	0.2285	0.221	3.01E-01
chicken_pox	hepatitis_a	-0.1138	0.3412	7.39E-01
chicken_pox	rheumatic_fever	0.4836	1.0632	6.49E-01
chicken_pox	colds_last_year	0.318	0.1438	2.70E-02
chicken_pox	rubella	0.4354	0.1648	8.20E-03
chicken_pox	chronic_sinus_infections	0.2496	0.178	1.61E-01
shingles	yeast_infections	0.102	0.1213	4.01E-01
shingles	tonsillectomy	0.0901	0.1056	3.94E-01
cold_sores	shingles	0.0793	0.2588	7.59E-01

**cold_sores**	**mononucleosis**	-0.0519	0.2951	8.61E-01
**cold_sores**	**mumps**	0.1327	0.1801	4.61E-01
**cold_sores**	**hepatitis_b**	0.1416	0.3705	7.02E-01
**cold_sores**	**plantar_warts**	-0.0918	0.2542	7.18E-01
**cold_sores**	**positive_tb_test**	0.6129	0.2828	3.02E-02
**cold_sores**	**strep_throat**	0.5522	0.2199	1.20E-02
**cold_sores**	**scarlet_fever**	0.3895	0.3048	2.01E-01
**cold_sores**	**pneumonia**	0.292	0.2142	1.73E-01
**cold_sores**	**bacterial_meningitis**	-0.1635	0.4693	7.28E-01
**cold_sores**	**yeast_infections**	0.394	0.171	2.12E-02
**cold_sores**	**uti_frequency**	0.0391	0.1892	8.36E-01
**cold_sores**	**tonsillectomy**	0.0542	0.1229	6.59E-01
**cold_sores**	**childhood_ear_infections**	0.0914	0.1306	4.84E-01
**cold_sores**	**myringotomy**	0.2099	0.2283	3.58E-01
**cold_sores**	**measles**	0.2092	0.3131	5.04E-01
**cold_sores**	**hepatitis_a**	0.0143	0.505	9.77E-01
**cold_sores**	**rheumatic_fever**	0.7053	1.3457	6.00E-01
**cold_sores**	**colds_last_year**	0.3259	0.2015	1.06E-01
**cold_sores**	**rubella**	-0.0464	0.2111	8.26E-01
**cold_sores**	**chronic_sinus_infections**	0.5296	0.3229	1.01E-01
**mononucleosis**	**shingles**	0.0483	0.2326	8.36E-01
**mononucleosis**	**mumps**	-0.2597	0.1794	1.48E-01
**mononucleosis**	**plantar_warts**	0.2935	0.2367	2.15E-01
**mononucleosis**	**positive_tb_test**	-0.0177	0.2662	9.47E-01
**mononucleosis**	**strep_throat**	0.3908	0.185	3.47E-02
**mononucleosis**	**scarlet_fever**	0.3267	0.2656	2.19E-01
**mononucleosis**	**pneumonia**	0.6705	0.1853	3.00E-04

**mononucleosis**	**yeast_infections**	0.3808	0.1783	3.27E-02
**mononucleosis**	**uti_frequency**	0.227	0.1457	1.19E-01
**mononucleosis**	**tonsillectomy**	0.5699	0.1644	5.00E-04
**mononucleosis**	**myringotomy**	0.1038	0.1918	5.88E-01
**mononucleosis**	**measles**	-0.1403	0.3236	6.65E-01
**mononucleosis**	**rheumatic_fever**	0.7387	1.5079	6.24E-01
**mononucleosis**	**rubella**	0.0288	0.2231	8.97E-01
**mumps**	**shingles**	-0.0671	0.147	6.48E-01
**mumps**	**plantar_warts**	0.3344	0.1589	3.53E-02
**mumps**	**positive_tb_test**	0.321	0.1868	8.57E-02
**mumps**	**scarlet_fever**	0.0374	0.181	8.36E-01
**mumps**	**pneumonia**	0.1227	0.11	2.65E-01
**mumps**	**yeast_infections**	-0.097	0.0985	3.25E-01
**mumps**	**tonsillectomy**	0.0283	0.0701	6.87E-01
**mumps**	**myringotomy**	-0.1919	0.1324	1.47E-01
**mumps**	**rheumatic_fever**	0.4714	0.8984	6.00E-01
**mumps**	**rubella**	**0.5539**	**0.1375**	**5.61E-05**
**hepatitis_b**	**shingles**	-0.2413	0.2967	4.16E-01
**hepatitis_b**	**mononucleosis**	0.3269	0.3524	3.54E-01
**hepatitis_b**	**mumps**	-0.2281	0.2273	3.16E-01
**hepatitis_b**	**plantar_warts**	0.3165	0.3074	3.03E-01
**hepatitis_b**	**positive_tb_test**	0.2556	0.3395	4.52E-01
**hepatitis_b**	**strep_throat**	0.1699	0.2392	4.78E-01
**hepatitis_b**	**scarlet_fever**	-0.5076	0.3762	1.77E-01
**hepatitis_b**	**pneumonia**	0.0499	0.2045	8.07E-01
**hepatitis_b**	**bacterial_meningitis**	-0.612	0.5844	2.95E-01
**hepatitis_b**	**yeast_infections**	0.1114	0.1876	5.52E-01

**hepatitis_b**	**uti_frequency**	0.1359	0.204	5.05E-01
**hepatitis_b**	**tonsillectomy**	0.115	0.1311	3.80E-01
**hepatitis_b**	**childhood_ear_infections**	0.0482	0.1478	7.44E-01
**hepatitis_b**	**myringotomy**	-0.1156	0.2895	6.90E-01
**hepatitis_b**	**measles**	0.0698	0.368	8.50E-01
**hepatitis_b**	**rheumatic_fever**	0.7568	1.6701	6.51E-01
**hepatitis_b**	**rubella**	0.1271	0.2926	6.64E-01
**hepatitis_b**	**chronic_sinus_infections**	0.1871	0.3493	5.92E-01
**plantar_warts**	**shingles**	-0.1514	0.2206	4.93E-01
**plantar_warts**	**positive_tb_test**	0.2548	0.2269	2.61E-01
**plantar_warts**	**scarlet_fever**	-0.0814	0.2104	6.99E-01
**plantar_warts**	**pneumonia**	-0.0191	0.141	8.92E-01
**plantar_warts**	**yeast_infections**	0.1724	0.1257	1.70E-01
**plantar_warts**	**tonsillectomy**	0.141	0.1126	2.11E-01
**plantar_warts**	**rheumatic_fever**	0.0601	0.721	9.34E-01
**plantar_warts**	**rubella**	0.3737	0.1977	5.87E-02
**positive_tb_test**	**shingles**	0.047	0.2045	8.18E-01
**positive_tb_test**	**scarlet_fever**	0.3219	0.2266	1.56E-01
**positive_tb_test**	**yeast_infections**	0.2325	0.1509	1.23E-01
**positive_tb_test**	**tonsillectomy**	0.0851	0.1061	4.23E-01
**positive_tb_test**	**rheumatic_fever**	0.258	0.8357	7.58E-01
**positive_tb_test**	**rubella**	0.2304	0.2052	2.61E-01
**strep_throat**	**shingles**	0.3219	0.1478	2.94E-02
**strep_throat**	**mumps**	0.1577	0.1234	2.01E-01
**strep_throat**	**plantar_warts**	0.2454	0.1763	1.64E-01
**strep_throat**	**positive_tb_test**	0.1447	0.1706	3.96E-01
**strep_throat**	**scarlet_fever**	0.2591	0.1652	1.17E-01

**strep_throat**	**pneumonia**	**0.6592**	**0.0954**	**4.88E-12**
**strep_throat**	**yeast_infections**	0.3618	0.1006	3.00E-04
**strep_throat**	**uti_frequency**	0.3594	0.1048	6.00E-04
**strep_throat**	**tonsillectomy**	**0.7536**	**0.0719**	**1.07E-25**
**strep_throat**	**myringotomy**	0.469	0.1513	1.90E-03
**strep_throat**	**measles**	0.0981	0.1935	6.12E-01
**strep_throat**	**rheumatic_fever**	0.7205	1.5091	6.33E-01
**strep_throat**	**rubella**	0.1839	0.1366	1.78E-01
**scarlet_fever**	**shingles**	-0.0482	0.2294	8.33E-01
**scarlet_fever**	**yeast_infections**	-0.0507	0.1409	7.19E-01
**scarlet_fever**	**tonsillectomy**	0.1381	0.11	2.09E-01
**pneumonia**	**shingles**	0.2124	0.1404	1.30E-01
**pneumonia**	**positive_tb_test**	0.079	0.1822	6.65E-01
**pneumonia**	**scarlet_fever**	0.5255	0.1705	2.10E-03
**pneumonia**	**yeast_infections**	0.2388	0.0893	7.50E-03
**pneumonia**	**tonsillectomy**	**0.2629**	**0.0674**	**9.64E-05**
**pneumonia**	**rheumatic_fever**	1	1.7975	5.71E-01
**pneumonia**	**rubella**	0.4115	0.1313	1.70E-03
**bacterial_meningitis**	**shingles**	0.7877	0.5731	1.69E-01
**bacterial_meningitis**	**mononucleosis**	0.5371	0.5484	3.27E-01
**bacterial_meningitis**	**mumps**	0.1303	0.3414	7.03E-01
**bacterial_meningitis**	**plantar_warts**	0.5492	0.474	2.47E-01
**bacterial_meningitis**	**positive_tb_test**	0.834	0.5849	1.54E-01
**bacterial_meningitis**	**strep_throat**	-0.1523	0.2737	5.78E-01
**bacterial_meningitis**	**scarlet_fever**	-0.1715	0.4364	6.94E-01
**bacterial_meningitis**	**pneumonia**	0.5124	0.365	1.60E-01
**bacterial_meningitis**	**yeast_infections**	0.3857	0.2908	1.85E-01

**bacterial_meningitis**	**uti_frequency**	0.3972	0.3159	2.09E-01
**bacterial_meningitis**	**tonsillectomy**	0.0715	0.1951	7.14E-01
**bacterial_meningitis**	**childhood_ear_infections**	0.3429	0.2848	2.29E-01
**bacterial_meningitis**	**myringotomy**	0.5817	0.4392	1.85E-01
**bacterial_meningitis**	**measles**	0.7711	0.6997	2.70E-01
**bacterial_meningitis**	**rheumatic_fever**	0.0469	1.2397	9.70E-01
**bacterial_meningitis**	**rubella**	-0.2077	0.3536	5.57E-01
**bacterial_meningitis**	**chronic_sinus_infections**	0.5833	0.5466	2.86E-01
**uti_frequency**	**shingles**	0.0145	0.1452	9.21E-01
**uti_frequency**	**mumps**	-0.0198	0.1062	8.52E-01
**uti_frequency**	**plantar_warts**	-0.003	0.1236	9.81E-01
**uti_frequency**	**positive_tb_test**	-0.115	0.1543	4.56E-01
**uti_frequency**	**scarlet_fever**	0.0543	0.1745	7.56E-01
**uti_frequency**	**pneumonia**	**0.3882**	**0.0946**	**4.05E-05**
**uti_frequency**	**yeast_infections**	**0.6471**	**0.0793**	**3.36E-16**
**uti_frequency**	**tonsillectomy**	0.1546	0.0633	1.46E-02
**uti_frequency**	**myringotomy**	0.0589	0.126	6.40E-01
**uti_frequency**	**measles**	-0.1271	0.1885	5.00E-01
**uti_frequency**	**rheumatic_fever**	0.0196	0.4227	9.63E-01
**uti_frequency**	**rubella**	0.0746	0.1248	5.50E-01
**tonsillectomy**	**yeast_infections**	0.2166	0.0653	9.00E-04
**childhood_ear_infections**	**shingles**	0.0646	0.0934	4.89E-01
**childhood_ear_infections**	**mononucleosis**	**0.5251**	**0.1414**	**2.00E-04**
**childhood_ear_infections**	**mumps**	0.0258	0.0994	7.95E-01
**childhood_ear_infections**	**plantar_warts**	0.2163	0.1146	5.93E-02
**childhood_ear_infections**	**positive_tb_test**	0.2377	0.1117	3.33E-02
**childhood_ear_infections**	**strep_throat**	**0.4425**	**0.0711**	**4.91E-10**

**childhood_ear_infections**	**scarlet_fever**	0.1431	0.1059	1.76E-01
**childhood_ear_infections**	**pneumonia**	**0.3214**	**0.0684**	**2.65E-06**
**childhood_ear_infections**	**yeast_infections**	0.1334	0.069	5.32E-02
**childhood_ear_infections**	**uti_frequency**	0.1919	0.0637	2.60E-03
**childhood_ear_infections**	**tonsillectomy**	**0.4522**	**0.046**	**8.85E-23**
**childhood_ear_infections**	**myringotomy**	**0.7866**	**0.1025**	**1.61E-14**
**childhood_ear_infections**	**measles**	0.1706	0.1603	2.87E-01
**childhood_ear_infections**	**rheumatic_fever**	0.3147	0.737	6.69E-01
**childhood_ear_infections**	**rubella**	0.2648	0.1038	1.08E-02
**childhood_ear_infections**	**chronic_sinus_infections**	0.0892	0.1054	3.97E-01
**myringotomy**	**shingles**	-0.2828	0.1713	9.89E-02
**myringotomy**	**plantar_warts**	0.1773	0.1882	3.46E-01
**myringotomy**	**positive_tb_test**	0.2097	0.2011	2.97E-01
**myringotomy**	**scarlet_fever**	0.1532	0.1899	4.20E-01
**myringotomy**	**pneumonia**	0.0873	0.1157	4.51E-01
**myringotomy**	**yeast_infections**	0.1726	0.106	1.03E-01
**myringotomy**	**tonsillectomy**	**0.4944**	**0.0883**	**2.15E-08**
**myringotomy**	**rheumatic_fever**	0.2819	0.9128	7.57E-01
**myringotomy**	**rubella**	-0.213	0.1821	2.42E-01
**measles**	**shingles**	0.342	0.2977	2.51E-01
**measles**	**mumps**	0.699	0.2331	2.70E-03
**measles**	**plantar_warts**	0.231	0.2797	4.09E-01
**measles**	**positive_tb_test**	0.6561	0.3159	3.78E-02
**measles**	**scarlet_fever**	0.2812	0.2849	3.24E-01
**measles**	**pneumonia**	0.2511	0.1839	1.72E-01
**measles**	**yeast_infections**	-0.0947	0.1697	5.77E-01
**measles**	**tonsillectomy**	0.0667	0.123	5.88E-01

**measles**	**myringotomy**	0.0021	0.2245	9.92E-01
**measles**	**rheumatic_fever**	-0.1166	1.4376	9.35E-01
**measles**	**rubella**	0.7932	0.2536	1.80E-03
**hepatitis_a**	**shingles**	-0.4216	0.3932	2.84E-01
**hepatitis_a**	**mononucleosis**	0.5197	0.4286	2.25E-01
**hepatitis_a**	**mumps**	-0.2513	0.2998	4.02E-01
**hepatitis_a**	**hepatitis_b**	-0.167	0.5448	7.59E-01
**hepatitis_a**	**plantar_warts**	0.1158	0.4032	7.74E-01
**hepatitis_a**	**positive_tb_test**	-0.5549	0.4696	2.37E-01
**hepatitis_a**	**strep_throat**	0.4636	0.3744	2.16E-01
**hepatitis_a**	**scarlet_fever**	0.0328	0.4811	9.46E-01
**hepatitis_a**	**pneumonia**	0.176	0.2299	4.44E-01
**hepatitis_a**	**bacterial_meningitis**	-0.6712	0.6722	3.18E-01
**hepatitis_a**	**yeast_infections**	0.0618	0.2247	7.83E-01
**hepatitis_a**	**uti_frequency**	0.3997	0.2972	1.79E-01
**hepatitis_a**	**tonsillectomy**	0.1742	0.1783	3.29E-01
**hepatitis_a**	**childhood_ear_infections**	0.0256	0.1989	8.98E-01
**hepatitis_a**	**myringotomy**	0.0114	0.3256	9.72E-01
**hepatitis_a**	**measles**	-0.747	0.5189	1.50E-01
**hepatitis_a**	**rheumatic_fever**	0.7013	1.7233	6.84E-01
**hepatitis_a**	**rubella**	-0.131	0.3427	7.02E-01
**hepatitis_a**	**chronic_sinus_infections**	0.1485	0.4009	7.11E-01
**rheumatic_fever**	**shingles**	0.8182	1.6497	6.20E-01
**rheumatic_fever**	**scarlet_fever**	0.1156	0.7766	8.82E-01
**rheumatic_fever**	**yeast_infections**	-0.4859	1.0166	6.33E-01
**rheumatic_fever**	**tonsillectomy**	0.4736	0.8765	5.89E-01
**rheumatic_fever**	**rubella**	0.225	0.7623	7.68E-01

**colds_last_year**	**shingles**	-0.0588	0.1516	6.98E-01
**colds_last_year**	**mononucleosis**	0.1656	0.1819	3.63E-01
**colds_last_year**	**mumps**	0.3052	0.1395	2.87E-02
**colds_last_year**	**hepatitis_b**	-0.417	0.26	1.09E-01
**colds_last_year**	**plantar_warts**	0.1839	0.1513	2.24E-01
**colds_last_year**	**positive_tb_test**	0.1881	0.1785	2.92E-01
**colds_last_year**	**strep_throat**	0.3904	0.1189	1.00E-03
**colds_last_year**	**scarlet_fever**	0.144	0.1863	4.39E-01
**colds_last_year**	**pneumonia**	**0.419**	**0.1134**	**2.00E-04**
**colds_last_year**	**bacterial_meningitis**	-0.1324	0.2949	6.53E-01
**colds_last_year**	**yeast_infections**	0.1211	0.0983	2.18E-01
**colds_last_year**	**uti_frequency**	0.1931	0.1089	7.61E-02
**colds_last_year**	**tonsillectomy**	0.1007	0.0714	1.58E-01
**colds_last_year**	**childhood_ear_infections**	0.2011	0.0725	5.50E-03
**colds_last_year**	**myringotomy**	0.099	0.1313	4.51E-01
**colds_last_year**	**measles**	0.3282	0.2253	1.45E-01
**colds_last_year**	**hepatitis_a**	0.0477	0.2956	8.72E-01
**colds_last_year**	**rheumatic_fever**	-0.0483	0.528	9.27E-01
**colds_last_year**	**rubella**	0.2346	0.1548	1.30E-01
**colds_last_year**	**chronic_sinus_infections**	0.4504	0.1854	1.51E-02
**rubella**	**shingles**	0.2808	0.2034	1.67E-01
**rubella**	**scarlet_fever**	0.6706	0.2325	3.90E-03
**rubella**	**yeast_infections**	0.0528	0.1154	6.47E-01
**rubella**	**tonsillectomy**	0.0623	0.082	4.48E-01
**chronic_sinus_infections**	**shingles**	0.1133	0.1968	5.65E-01
**chronic_sinus_infections**	**mononucleosis**	1	0.3526	2.60E-03
**chronic_sinus_infections**	**mumps**	0.0635	0.1813	7.26E-01

**chronic_sinus_infections**	**plantar_warts**	-0.0716	0.2213	7.46E-01
**chronic_sinus_infections**	**positive_tb_test**	0.2574	0.2682	3.37E-01
**chronic_sinus_infections**	**strep_throat**	0.5207	0.1748	2.90E-03
**chronic_sinus_infections**	**scarlet_fever**	-0.0271	0.2721	9.21E-01
**chronic_sinus_infections**	**pneumonia**	0.6062	0.1673	3.00E-04
**chronic_sinus_infections**	**yeast_infections**	0.5249	0.1495	4.00E-04
**chronic_sinus_infections**	**uti_frequency**	0.4081	0.1505	6.70E-03
**chronic_sinus_infections**	**tonsillectomy**	0.347	0.1095	1.50E-03
**chronic_sinus_infections**	**myringotomy**	0.1449	0.1983	4.65E-01
**chronic_sinus_infections**	**measles**	0.0653	0.3053	8.31E-01
**chronic_sinus_infections**	**rheumatic_fever**	0.2092	0.7107	7.69E-01
**chronic_sinus_infections**	**rubella**	0.4312	0.2239	5.42E-02

rg: estimated genetic correlation from LD score regression; **rg_se**: standard error of rg; **rg_p**: p-value for rg.

Supplementary Table 7: The heritability estimation

	**LD score regression**				**GCTA-GREML**					**Heritability explained by GWAS significant SNP**
**Phenotype**	**h**	**se**	**h_L**	**se_L**	**V(G)/Vp**	**se**	**V(G)/Vp_L**	**se_L**	**p**	
chicken_pox	0.0193	0.0041	0.0512	0.0109	0.0800	0.0370	0.0890	0.0412	1.28E-02	0.00097
shingles	0.0143	0.0043	0.039	0.0118	0.0127	0.0353	0.0139	0.0387	3.54E-01	0.00305
cold_sores	0.0136	0.006	0.0247	0.0109	0.0385	0.0373	0.0547	0.0529	1.50E-01	0.00106
mononucleosis	0.0149	0.0062	0.031	0.013	0.0829	0.0377	0.1639	0.0744	1.22E-02	0.00085
mumps	0.0334	0.0073	0.0555	0.0122	0.0716	0.0381	0.1078	0.0575	3.06E-02	0.01460
hepatitis_b	0.0035	0.0022	0.0761	0.048	0.1333	0.0377	0.1322	0.0373	1.29E-04	0.00349
plantar_warts	0.0272	0.0084	0.0441	0.0135	0.0923	0.0368	0.1418	0.0565	4.20E-03	0.00650
positive_tb_test	0.0179	0.0065	0.0843	0.0306	0.1682	0.0364	0.1411	0.0306	9.80E-08	0.00959

strep_throat	0.0472	0.0075			0.0098	0.0367			3.94E-01	0.00099
scarlet_fever	0.0129	0.0041	0.0563	0.0179	0.1311	0.0372	0.1124	0.0319	8.62E-05	0.00310
pneumonia	0.033	0.0046	0.0579	0.008	0.0761	0.0381	0.1103	0.0552	2.34E-02	0.00090
bacterial_meningitis	0.0056	0.0058	0.083	0.0846	0.0509	0.0372	0.0896	0.0654	8.31E-02	0.00695
yeast_infections	0.0785	0.0094			0.1689	0.0387			5.28E-06	0.00221
uti_frequency	0.0697	0.0103			0.0593	0.0367			4.66E-02	0.00071
tonsillectomy	0.0662	0.0071	0.1123	0.012	0.1396	0.0378	0.2072	0.0561	7.09E-05	0.02323
childhood_ear_infections	0.0718	0.0065	0.1176	0.0106	0.0471	0.0378	0.0761	0.0611	1.06E-01	0.01098
myringotomy	0.0278	0.0066	0.1372	0.0327	0.1710	0.0381	0.1428	0.0318	1.53E-06	0.00239
measles	0.0118	0.0066	0.0188	0.0105	0.0000	0.0366	0.0000	0.0570	5.00E-01	
hepatitis_a	0.0025	0.0023	0.0352	0.0332	0.0641	0.0360	0.0475	0.0267	2.83E-02	
rheumatic_fever	0.0018	0.0051	0.0231	0.0641	0.0000	0.0369	0.0000	0.0531	5.00E-01	
colds_last_year	0.0464	0.0084			0.0462	0.0367			9.95E-02	
rubella	0.0237	0.0056	0.0589	0.0138	0.0033	0.0367	0.0038	0.0425	4.65E-01	
chronic_sinus_infections	0.0176	0.0062	0.0288	0.0102	0.0000	0.0364	0.0000	0.1359	5.00E-01	

LD score regression: h: observed scale heritability from LD score regression; **se**: standard error for h; **h_L**: liability scale heritability from LD score regression (the prevalence was set to be the same as the disease probability in our cohort); **se_L**: standard error for h_L; **GCTA-GREML**: **V(G)/Vp:** Observed scale heritability explained from GCTA; **V(G)/Vp _se:** standard error of V(G)/Vp; **V(G)/Vp_L:** liability scale heritability explained (the prevalence was set to be the same as the disease probability in our cohort); **V(G)/Vp_L:** standard error of V(G)/Vp_L; **p:** P-value of the estimated heritability explained by GCTA. **Variance explained by GWAS significant SNP:** The estimated liability scale heritability explained by the GWAS significant SNPs, see the Supplementary Note 4 “Heritability estimation of infectious diseases”.

Supplementary Table 8: Imputation quality (R^2) for detected HLA associations

Variants	Cytoband	Position	Alleles	Frequency	r^2
HLA-A Arg97	6p22.1	29911063	IRM	0.36	0.99
HLA-A Gln43	6p22.1	29910660	QR	0.99	0.98
HLA-A Gly107	6p22.1	29911093	GW	0.71	0.99
HLA-A*02:01	6p22.1	29911953	NA	0.27	0.98
HLA-A*02:05	6p22.1	29911953	NA	0.01	0.94
HLA-B GluMet45	6p21.33	31324602	EMTKG	0.52	0.98
HLA-B SerTrpAsn97	6p21.33	31324201	STWRNV	0.34	0.98
HLA-B ThrGly45	6p21.33	31324602	EMTKG	0.23	0.99
HLA-B Trp147	6p21.33	31324051	WL	0.95	0.97
HLA-B Val97	6p21.33	31324201	STWRNV	0.04	0.99
HLA-B*08:01	6p21.33	31323318	NA	0.11	1.00
HLA-B*44:02	6p21.33	31323318	NA	0.09	0.98
HLA-B*57:01	6p21.33	31323318	NA	0.04	0.99
HLA-DPB1 Arg194	6p21.32	33053577	QRx	0.86	0.72
HLA-DQA1 His129	6p21.32	32609872	QHx	0.66	0.98
HLA-DQA1*01:02	6p21.32	32608660	NA	0.20	0.97
HLA-DQA1*03:01	6p21.32	32608660	NA	0.10	0.86
HLA-DQB1 Gly45	6p21.32	32632724	GE	0.80	0.98
HLA-DQB1*03:01	6p21.32	32630853	NA	0.20	0.97
HLA-DQB1*06:02	6p21.32	32630853	NA	0.13	0.98
HLA-DRB1 Asp70	6p21.32	32551960	QDR	0.44	0.96
HLA-DRB1 CysTyr30	6p21.32	32552080	CYLGRH	0.83	0.96
HLA-DRB1 Gln96	6p21.32	32549612	EHYxQ	0.17	0.99
HLA-DRB1 Leu67	6p21.32	32551969	LIF	0.41	0.95
HLA-DRB1 LeuSer11	6p21.32	32552137	LSVGDP	0.52	0.99
HLA-DRB1 LeuValGly11	6p21.32	32552137	LSVGDP	0.42	1.00

HLA-DRB1 PheSerHis13	6p21.32	32552131	FSHYGR	0.66	0.97
HLA-DRB1 PheSerTyr13	6p21.32	32552131	FSHYGR	0.63	0.97
HLA-DRB1*04:01	6p21.32	32552079	NA	0.08	0.81
HLA-DRB1*11:01	6p21.32	32552079	NA	0.06	0.63
HLA-DRB1*15:01	6p21.32	32552079	NA	0.13	1.00

The allele "Frequency" and imputation quality "r^2" of the associated HLA alleles and amino acids

Supplementary Note 1: Surveys and phenotype scoring logic

The following section shows the surveys used to define each infectious disease phenotype in the 23andMe cohort.

Survey 1: Your Medical History

	**[Questions] Have you ever been diagnosed by a doctor with any of the following infectious conditions?**			
**Q1.1**	Chickenpox	Yes	No	I'm not sure
**Q1.2**	Shingles	Yes	No	I'm not sure
**Q1.3**	Cold sores	Yes	No	I'm not sure
**Q1.4**	Mumps	Yes	No	I'm not sure
**Q1.5**	Positive response to tuberculosis (TB) skin test, patch test, PPD (having positive TB test)	Yes	No	I'm not sure
**Q1.6**	Scarlet fever	Yes	No	I'm not sure
**Q1.7**	Pneumonia	Yes	No	I'm not sure
**Q1.8**	Tonsillectomy	Yes	No	I'm not sure
**Q1.9**	Myringotomy	Yes	No	I'm not sure
**Q1.10**	Measles	Yes	No	I'm not sure
**Q1.11**	Rheumatic fever	Yes	No	I'm not sure
**Q1.12**	Rubella	Yes	No	I'm not sure

Survey 2: Research Snippet:

	**[Questions]**			
**Q2.1**	Have you ever had Chickenpox?	Yes	No	I'm not sure
**Q2.2**	Have you ever received the chicken pox vaccine?	Yes	No	I'm not sure
**Q2.3**	Have you ever had shingles?	Yes	No	I'm not sure
**Q2.4**	Has a doctor ever diagnosed you with mono (Mononucleosis)?	Yes	No	I'm not sure
**Q2.5**	How many times have you had plantar warts on the soles of your feet?	Never, 1-2 times, 3-5 times, more than 5 times, I'm not sure		
**Q2.6**	How many times have you been told by a doctor that you had strep throat?	Never, 1-2 times, 3-5 times, More than 5 times, I'm not sure		
**Q2.7**	Have you ever been diagnosed with scarlet fever?	Yes	No	I'm not sure

**Q2.8**	Have you ever had bacterial meningitis?	Yes	No	I'm not sure
**Q2.9**	How many vaginal yeast infections have you had in your life	None, 1-2, 3-4, 5 or more		
**Q2.10**	How many times have you had a urinary tract infection (UTI)? (Please only count UTIs that were diagnosed by a urine test and treated with antibiotics)	Never, once, twice, three times, four or more times, I'm not sure		
**Q2.11**	Have you had your tonsils removed? (Tonsillectomy)	Yes	No	I'm not sure
**Q2.12**	Did you have several ear infections as a child (under the age of 18)?	Yes	No	I'm not sure
**Q2.13**	How many colds did you get over the last year? Do NOT include bouts of the flu.	None, 1-2, 3-4, 5 or more, I'm not sure		
**Q2.14**	Have you ever had surgery for chronic sinus infections?	Yes	No	I'm not sure

Survey 3: Health Intake

	**[Questions] Have you ever been diagnosed with or treated for any of the following conditions?**			
**Q3.1**	Hepatitis A	Yes	No	I'm not sure
**Q3.2**	Hepatitis B	Yes	No	I'm not sure

Logic for creating 23andMe phenotypes:

Chickenpox:

- Cases were customers who answered 'Yes' to either Q1.1 or Q2.1.
- Controls were customers who answered 'No' to either Q1.1 or Q2.1, and exclude customers who answered 'Yes' to Q2.2.
- Respondents with inconsistent responses for Q1.1 and Q2.1 were excluded

Shingles:

- Cases were customers who answered 'Yes' to either Q1.2 or Q2.3.
- Controls were customers who answered 'No' to either Q1.2 or Q2.3.
- Respondents with inconsistent responses for Q1.2 and Q2.3 were excluded

Cold sores:

- Cases were customers who answered 'Yes' to Q1.3.
- Controls were customers who answered 'No' to Q1.3

Mononucleosis:

- Cases were customers who answered 'Yes' to Q2.4
- Controls were customers who answered 'No' to Q2.4

Mumps:

- Cases were customers who answered 'Yes' to Q1.4.
- Controls were customers who answered 'No' to Q1.4

Hepatitis B:

- Cases were customers who answered 'Yes' to Q3.1
- Controls were customers who answered 'No' to Q3.1

Plantar warts:

- Quantitative trait from the responses to Q2.5. The first four responses are assigned scores from 0 to 3.

Positive TB test:

- Cases were customers who answered 'Yes' to Q1.5
- Controls were customers who answered 'No' to Q1.5

Strep throat:

- Quantitative trait from the responses to Q2.6. The first four responses are assigned scores from 0 to 3.

Scarlet fever:

- Cases were customers who answered 'Yes' to either Q1.6 or Q2.7.
- Controls were customers who answered 'No' to either Q1.6 or Q2.7.
- Respondents with inconsistent responses for Q1.6 and Q2.7 were excluded

Pneumonia:

- Cases were customers who answered 'Yes' to Q1.7
- Controls were customers who answered 'No' to Q1.7

Bacterial meningitis:

- Cases were customers who answered 'Yes' to Q2.8
- Controls were customers who answered 'No' to Q2.8

Yeast infections:

- Quantitative trait from the responses to Q2.9. The first four responses are assigned scores from 0 to 3.

UTI frequency:

- Quantitative trait from the responses to Q2.10. The first five responses are assigned scores from 0 to 4.

Tonsillectomy:

- Cases were customers who answered ‘Yes’ to either Q1.8 or Q2.11.
- Controls were customers who answered ‘No’ to either Q1.8 or Q2.11.
- Respondents with inconsistent responses for Q1.8 and Q2.11 were excluded

Childhood ear infection:

- Cases were customers who answered ‘Yes’ to Q2.12
- Controls were customers who answered ‘No’ to Q2.12

Myringotomy:

- Cases were customers who answered ‘Yes’ to Q1.9
- Controls were customers who answered ‘No’ to Q1.9

Measles:

- Cases were customers who answered ‘Yes’ to Q1.10
- Controls were customers who answered ‘No’ to Q1.10

Hepatitis A:

- Cases were customers who answered ‘Yes’ to Q3.2
- Controls were customers who answered ‘No’ to Q3.2

Rheumatic fever:

- Cases were customers who answered ‘Yes’ to Q1.11
- Controls were customers who answered ‘No’ to Q1.11

Colds last year:

- Quantitative trait from the responses to Q2.13. The first four responses are assigned scores from 0 to 3.

Rubella:

- Cases were customers who answered ‘Yes’ to Q1.12
- Controls were customers who answered ‘No’ to Q1.12

Chronic sinus infection:

- Cases were customers who answered ‘Yes’ to Q2.14
- Controls were customers who answered ‘No’ to Q2.14

Supplementary Note 2: Review of infectious diseases

Chickenpox and shingles (varicella-zoster virus)

Chickenpox and shingles are distinct clinical syndromes caused by the same member of the human herpesvirus family, varicella-zoster virus (VZV)[2]. Chickenpox

(varicella) results from primary VZV infection, which typically occurs in childhood. Like other human herpesviruses, VZV is not eliminated from the body following recovery from the clinical syndrome, but rather remains in latent form within the cell bodies of sensory neurons. In later life, when immunity wanes, approximately 20% of adults with a history of chickenpox develop shingles (herpes zoster) in the setting of viral reactivation within a dorsal root ganglion. Attenuated viral vaccines are now available for chickenpox and shingles and have decreased the incidence of both clinical syndromes[3,4].

Cold sores (type 1 herpes simplex virus, herpesvirus family)

Cold sores (herpes simplex labialis) are vesicular lesions of the lips and mouth caused by another member of the human herpesvirus family, herpes simplex virus type 1 (HSV-1). HSV-1 is extremely common, affecting over half of the US population[5]. Primary HSV-1 infection presents differently in different people, ranging from completely asymptomatic infections to outbreaks of painful vesicles, along with fever and malaise. Like VZV, HSV-1 persists in latent form within the cell bodies of sensory nerves following resolution of the primary infection. Recurrence is possible, particularly in the setting of stress or immunosuppression, and individuals differ tremendously in frequency and intensity of recurrent outbreaks[6].

Mononucleosis (Epstein-Barr virus, herpesvirus family)

Over 90% of the world's adult population is chronically infected with Epstein-Barr virus (EBV)[7]. Primary infection in children is usually asymptomatic or associated with only mild symptoms. When primary infection occurs later in life,

however, it often results in infectious mononucleosis (IM), a syndrome of fever, tonsillitis, swollen lymph nodes, and persistent fatigue. A longitudinal study of IM in a cohort of 2,823,583 Danish children found evidence of familial aggregation of IM[8].

Mumps (mumps virus)

Mumps is a syndrome of low-grade fever, malaise, headache, and characteristic swelling of the parotid (salivary) glands that is caused by the mumps virus[9]. Mumps usually resolves spontaneously, but is sometimes complicated by highly morbid inflammation of the testes (orchitis), meninges (meningitis), and brain (encephalitis), as well as deafness. This disease has been rare in developed countries since the introduction of routine vaccination in the 1960s; however, occasional outbreaks still occur in these countries, and mumps remains a significant health threat in developing countries[9].

Hepatitis A (hepatitis A virus)

Hepatitis A virus (HAV) causes acute, flu-like symptoms and abdominal pain, followed by jaundice in approximately 70% of infected adults, while being completely asymptomatic in the remainder[10]. HAV infection is self-limited in most cases, and fewer than 1% of progress to fulminant hepatitis. A hepatitis A vaccine is effective for prevention. No large cohort study has previously been performed for HAV infection.

Hepatitis B (hepatitis B virus)

Hepatitis B virus (HBV) is a major cause of acute and chronic hepatitis worldwide. The acute infection is asymptomatic in roughly 70% of cases; the

remaining 30% of infected individuals experience a variety of symptoms that can range from mild loss of appetite, nausea, right upper quadrant abdominal discomfort, and/or yellowish skin tone (jaundice) to fulminant liver failure and death[11]. Following recovery from the initial infection, the risk of progression to chronic hepatitis B (CHB) is strongly influenced by age. 90% of patients infected perinatally, as is common in sub-Saharan Africa and East Asia, progress to the chronic phase of infection, whereas fewer than 5% of patients who are infected as adults[12]. In CHB, the virus continues to reproduce in and cause damage to the liver at different rates in different patients. A vaccine against HBV is available, but the vaccine is not completely effective and coverage is not universal even in developed countries[13].

Plantar warts (human papillomavirus)

Plantar warts (verruca plana) are areas of tough, thickened skin on the soles of the feet caused by certain subtypes of human papillomavirus (HPV)[14]. Different HPV subtypes are responsible for common skin warts, anogenital warts, and certain forms of cancer[15].

Positive tuberculosis test (*Mycobacterium tuberculosis*)

Mycobacterium tuberculosis (TB) is the leading cause of infectious mortality worldwide, responsible for ~9 million new infections and ~1.5 million deaths each year[16]. Infections are spread through the inhalation of microaerosolized droplets, resulting in either pneumonia or asymptomatic latent infection. If untreated, the latent disease can reactivate in subsequent years to produce pneumonias and a

variety of other infections. The classic screening test for latent TB is the Tuberculin Skin Test (TST or PPD), which tests for a delayed hypersensitivity reaction to intradermally placed TB antigens[17]. The phenotype used in our GWAS was defined by asking customers whether they had ever had a positive response (skin induration) to a TST. A positive TST reaction indicates that a patient is infected with TB bacteria, whether in the form of active or latent disease; occasional 'false positives' occur in patients who have recently received the Bacillus Calmette-Guérin vaccine. A negative TB test indicates that latent TB infection or TB disease is unlikely.

Strep throat and scarlet Fever (group A streptococcus)

'Strep throat', caused by *Streptococcus pyogenes* (Group A Streptococcus), classically presents with the abrupt onset of throat pain, fever, headache, and tender cervical lymph nodes[18]. *S. pyogenes* strains that express pyrogenic exotoxins can cause scarlet fever, a syndrome of strep throat plus a generalized sandpaper-like rash. In a minority of patients, these infections lead to maladaptive host immune responses that result in serious additional complications, including rheumatic fever, post-streptococcal glomerulonephritis, and toxic shock syndrome. The risk for immune-mediated complications can be reduced by prompt diagnosis of strep throat and treatment with effective antibiotics, but it remains unclear why *S. pyogenes* infections produce such different outcomes in different hosts.

Pneumonia (multiple pathogens)

Pneumonia refers to infection of lung tissue by bacteria, viruses or, less commonly, fungi. The most frequent route of infection is typically microaspiration of pathogens that colonize the upper airways, with less common routes including dissemination from the blood, gross aspiration of stomach contents, and invasion from adjacent structures. The most common cause of bacterial pneumonia in adults, isolated in nearly 50% of community-acquired cases, is *Streptococcus pneumoniae*[19].

Bacterial meningitis (various pathogenic bacteria)

Bacterial meningitis is a severe infection of tissues surrounding the brain and spinal cord that results in substantial neurological morbidity and mortality worldwide. *Streptococcus pneumoniae* is the leading cause of bacterial meningitis in adults. The overall incidence of meningitis has declined since the introduction of vaccines against *Streptococcus pneumonia* and *Haemophilus influenza* in developed countries, although the number of cases due to *Streptococcus pneumonia* serotypes not included in the vaccines has increased[20].

Vaginal yeast infection (*Candida albicans, Candida glabrata*)

Vaginal yeast infections (Candida vulvovaginitis, or thrush) are localized fungal infections of the vagina and/or vulva caused by overgrowth of *Candida albicans* or, less commonly, *Candida glabrata* and other *Candida spp.*. These self-limited, superficial infections are extremely common in women, such that 70-75% of women experience at least one episode during their lives[21]. A subset of women suffer from recurrent yeast infections, a syndrome that has previously been linked to polymorphisms in mannose binding lectin[22].

Urinary Tract Infection (various pathogenic bacteria)

Urinary tract infections (UTIs) involving the bladder (cystitis) are extremely common in otherwise healthy women and typically produce symptoms of frequency, urgency, and pain with urination. In the minority of UTIs in which the kidneys are also infected (pyelonephritis), symptoms may progress to include fevers, chills, flank pain, nausea, vomiting, and weakness. About 80-85% of UTIs are caused by *Escherichia coli*, 5-10% are caused by *Staphylococcus saprophyticus,* and other fecal flora account for the majority of the remaining infections[23]. Very rarely, viruses or fungi may cause UTIs. UTIs are far more common in women than men[24] and in our dataset, significantly more female than male customers reported having had two or more UTIs.

Tonsillectomy (multiple origins)

The palatine tonsils are masses of lymphoid tissue positioned on the right and left sides of the posterior throat. Together with the adenoids, they are considered the first line of defense against inhaled and ingested pathogens. Infection of the tonsils with bacteria (typically *S. pyogenes*) or viruses causes tonsillitis, a painful inflammation that is particularly common in childhood. In cases of intractable, recurrent tonsillitis or tonsillar hypertrophy, tonsillectomy (surgical removal of the tonsils) may be offered to decrease the severity and frequency of recurrences[25].

Childhood ear infection and myringotomy (multiple origins)

Middle ear infections (otitis media) are very common in young children[26]. The initial insult is often a viral infection that produces inflammation and obstruction of the Eustachian tube that normally drains the middle ear. Bacteria (such as *S. pneumoniae, Haemophilus influenza, Moraxella catarrhalis)* and/or viruses (such as

rhinovirus, respiratory syncytial virus, influenza, parainfluenza, various enteroviruses) from the nasopharynx then seed and proliferate within the accumulated fluid causing pain, swelling, and characteristic bulging of the ear drum[27]. Children under three years old are most vulnerable because their Eustachian tubes are less effective at draining fluid and their immune systems are not yet fully developed. For children with a history of frequent, recurrent otitis media, surgical incision of the eardrum (myringotomy) and placement of a small tympanostomy tube to drain the infected fluid has been shown to reduce the frequency of recurrences and to improve quality of life[28,29].

Supplementary Note 3: Genetic correlations of infectious diseases

We investigated the amount of shared genetics among the common infections with a co-heritability analysis using LD score regression[30] (Supplementary Figure 4 and Supplementary Table 6). We computed LD scores as previously described[31] using the samples in the 1000 Genomes Project reference panel (phase 3 version 5a) with a MAF cutoff of 5% and imputation r-square cutoff of 0.9. We calculated the pairwise genetic correlation (r_g) on the GWAS summary statistics (including MHC region) using LD score regression[30]. The heat map of genetic correlation was plotted using python.

We found significant positive genetic correlations between strep throat and tonsillectomy (r_g=0.75, se=0.07, P=1.07x10^{-25}) and between childhood ear infection and myringotomy (r_g=0.79, se=0.10, P=1.61x10^{-14}), which are pairs of related phenotypes. We also observed a high genetic correlation between UTIs and yeast infections (rg=0.65, se=0.08, P=3.36x10^{-16}), as well as a phenotypic correlation (Pearson r=0.41, P<2.2x10^{-16}) among women who reported both UTIs and yeast infections in our cohort. This observation most likely stems from the fact that UTIs are usually treated with antibiotics, which are a strong risk factor for the development of subsequent yeast infections (likely reflecting overgrowth of yeasts when normal bacterial commensals are killed)[21]. Mumps, measles and rubella formed a cluster in the heat map plot in Supplementary Figure 4, where a high genetic correlation was found between mumps and rubella (rg=0.55, se=0.14, P=5.61x10^{-5}), between mumps and measles (rg=0.70, se=0.23, P=2.70x10^{-3}), and

between measles and rubella (rg=0.79, se=0.25, P=1.80×10^{-3}), although the last two pairs were not statistically significant after multiple test correction. The MMR vaccine was introduced in 1960s and participants younger than 60 were mostly vaccinated and free of MMR. Our case cohorts for mumps, measles and rubella contained a much higher percent of adults (age>60) than control cohorts (Supplementary Table 2). Age could be a strong confounding factor that creates a correlation between association statistics across the three diseases, which is a limitation in our study.

A highly significant genetic correlation was also found between tonsillectomy and childhood ear infections (r_g=0.45, se=0.05, P=8.85×10^{-23}). Tonsillitis and ear infections sometimes co-occur and may be caused by many of the same viral and bacterial pathogens. A previous twin study suggested a substantial overlap in genetic factors influencing risk for both ear infection and tonsillitis[32]. In our GWASes, we identified the missense mutation (rs72646967-C, N397H) in *TBX1* as a significant protective locus for both (Supplementary Figure 5B). Likewise, significant positive correlations were observed for pneumonia with strep throat (rg=0.66, se=0.10, P=4.88×10^{-12}), childhood ear infections (rg=0.32, se=0.07, P=2.65×10^{-6}), tonsillectomy (rg=0.26, se=0.07, P=9.64×10^{-5}) and common colds in the previous year (rg=0.42, se=0.11, P=2.00×10^{-4}). As described above, the most common route of infection for pneumonia is microaspiration of upper airway and nasopharyngeal pathogens, many of which (for example, *Streptococcus pneumoniae, Haemophilus influenzae,* and others) also causes sinusitis and otitis media. Moreover, many of the same viruses that cause colds and sore throats also

predispose to middle ear infections and sinusitis and can directly cause pneumonia[33] or act as co-pathogens with bacteria[34].

Supplementary Note 4: Heritability estimation of infectious diseases

We used LD score regression[31] and genomic-relatedness-based restricted maximum-likelihood method implemented genome-wide complex trait analysis (GCTA-GREML) tool[35] to estimate the proportion of phenotype variation that can be explained by genome-wide SNPs (h_g^2), which is the 'narrow sense heritability' or additive 'SNP-heritability'. For running LD score regression, only a subset of SNPs (~1 million) that have pre-calculated LD scores were used and no rare variants were included. For running the GCTA tools, only genotyped SNPs (~ 1 million) were used. GCTA requires genotype data as input and cannot be easily applied on large cohort, and thus we sampled 5000 cases + 5000 control (10,000 samples if quantitative trait). To calculate the heritability in liability scale, we set the disease prevalence according to the frequency of diseases in the original cohort. A prior study calculated the power of GCTA-GREML[36] and estimated that 10,000 samples will have enough power to detect any SNP heritability > 0.03 at various disease prevalence scenarios. The power decreases significantly when the sample size drops to fewer than 2000. Power for LD score regression are expected to be lower than GCTA-GREML due to the cost of using summary statistics rather than genotypes. The liability scale heritability estimated from LD score regression and GCTA-GREML are slightly different and larger confidence intervals were observed by GCTA-GRML analysis for some infections (Supplementary Table 7), which is probably due to the

sub-sampling of much smaller sample sizes used in GCTA-GRML analysis. For example, the SNP heritability of childhood ear infections from LD score regression was 11.8% (se=1.06%), while the GCTA-GREML had a non-significant estimate of 7.61% (se=6.11%). The SNP heritability of tonsillectomy is the highest among all the infectious phenotypes studied here; LD score regression estimated 11.23% (se=1.2%) and GCTA-GREML estimated 20.72% (se=5.61%). Earlier twin studies calculated that heredity accounts for over 70% of susceptibility to recurrent ear infections in children[37] and also suggested that there is a substantial genetic predisposition for recurrent tonsillitis[38] and sleep-disordered breathing[39]. First, it is important to remember that the h_g^2 is much smaller than an h^2 estimate from a twin study that includes the contributions of all genetic variation, not just common SNPs's additive contribution. The twin study estimator of h^2 can be seen as an upper bound while h_g^2 is a lower bound of genetic heritability estimate[40]. The actual genetic effect can be much larger due to rare SNPs that are not analyzed in the GWAS and SNP-SNP or SNP-environmental higher order interactions. Insignificant estimation of heritability (e.g. bacterial meningitis has only 842 cases) may also represent the lack of power. In addition, for infectious disease where a pathogen is required for the disease, the ideal study design would be case and controls being at equal risk of the necessary exposure. In our study, the controls sometimes contain vaccinated individuals or there is heterogeneity in the cases (e.g. more than 200 types of viruses can cause a cold), this could severely reduce the GWAS power and also the heritability estimation.

In addition, we calculated the heritability explained by the GWAS significant SNPs (index SNPs with $P<5x10^{-8}$) as previously described[41] for case-control phenotypes. For quantitative traits, we used the coefficient of determination R^2 from linear regression to calculate the phenotype variance explained by the GWAS significant associations. The heritability explained by the GWS SNPs is much less than 100% of estimated SNP heritability (Supplementary Table 7), suggesting additional disease-associations remain to be discovered through more carefully designed studies.

Supplementary References

1. Pruim, R. J. *et al.* LocusZoom: regional visualization of genome-wide association scan results. *Bioinforma. Oxf. Engl.* **26,** 2336–2337 (2010).
2. Whitley, RJ. 'Chickenpox and Herpes Zoster,'. in *Mandell, Douglas, and Bennett's principles and practice of infectious diseases.* (eds. Bennett, J. E., Dolin, R. & Blaser, M. J.) 1731–1737 (Elsevier/Saunders, 2015).
3. Marin, M., Marti, M., Kambhampati, A., Jeram, S. M. & Seward, J. F. Global Varicella Vaccine Effectiveness: A Meta-analysis. *Pediatrics* **137,** e20153741 (2016).
4. Gagliardi, A. M. Z., Gomes Silva, B. N., Torloni, M. R. & Soares, B. G. O. Vaccines for preventing herpes zoster in older adults. *Cochrane Database Syst. Rev.* **10,** CD008858 (2012).
5. Xu, F. *et al.* Trends in herpes simplex virus type 1 and type 2 seroprevalence in the United States. *JAMA J. Am. Med. Assoc.* **296,** 964–973 (2006).

6. Schiffer, JT & Corey, L. Herpes Simplex Virus. in *Mandell, Douglas, and Bennett's principles and practice of infectious diseases.* (eds. Bennett, J. E., Dolin, R. & Blaser, M. J.) 1713–1730 (Elsevier/Saunders, 2015).
7. Johannsen, EC & Kaye, KM. Epstein-Barr Virus (Infectious Mononucleosis, Epstein Barr Virus-Associated Malignant Diseases, and Other Diseases). in *Mandell, Douglas, and Bennett's principles and practice of infectious diseases.* (eds. Bennett, J. E., Dolin, R. & Blaser, M. J.) 1754–1771 (Elsevier/Saunders, 2015).
8. Rostgaard, K., Wohlfahrt, J. & Hjalgrim, H. A genetic basis for infectious mononucleosis: evidence from a family study of hospitalized cases in Denmark. *Clin. Infect. Dis. Off. Publ. Infect. Dis. Soc. Am.* **58,** 1684–1689 (2014).
9. Litman, N & Baum, SG. Mumps Virus. in *Mandell, Douglas, and Bennett's principles and practice of infectious diseases.* (eds. Bennett, J. E., Dolin, R. & Blaser, M. J.) 1942–1947 (Elsevier/Saunders, 2015).
10. Averhoff, F & Khudyakov, Y. Hepatitis A Virus. in *Mandell, Douglas, and Bennett's principles and practice of infectious diseases.* (eds. Bell, BP, Bennett, B., Dolin, R. & Blaser, B.) 2095–2112 (Elsevier/Saunders, 2015).
11. Thio, CL & Hawkins, C. Hepatitis B Virus and Hepatitis Delta Virus. in *Mandell, Douglas, and Bennett's principles and practice of infectious diseases.* (eds. Bennett, J. E., Dolin, R. & Blaser, M. J.) 1815–1839 (Elsevier/Saunders, 2015).
12. Schweitzer, A., Horn, J., Mikolajczyk, R. T., Krause, G. & Ott, J. J. Estimations of worldwide prevalence of chronic hepatitis B virus infection: a systematic review of data published between 1965 and 2013. *Lancet Lond. Engl.* **386,** 1546–1555 (2015).

13. Chen, D.-S. Hepatitis B vaccination: The key towards elimination and eradication of hepatitis B. *J. Hepatol.* **50,** 805–816 (2009).
14. Cardoso, J. C. & Calonje, E. Cutaneous manifestations of human papillomaviruses: a review. *Acta Dermatovenerol. Alp. Pannonica Adriat.* **20,** 145–154 (2011).
15. Mighty, K. K. & Laimins, L. A. The role of human papillomaviruses in oncogenesis. *Recent Results Cancer Res. Fortschritte Krebsforsch. Prog. Dans Rech. Sur Cancer* **193,** 135–148 (2014).
16. Dheda, K., Barry, C. E. & Maartens, G. Tuberculosis. *Lancet Lond. Engl.* **387,** 1211–1226 (2016).
17. Raviglione, M. & Sulis, G. Tuberculosis 2015: Burden, Challenges and Strategy for Control and Elimination. *Infect. Dis. Rep.* **8,** 6570 (2016).
18. Bryan, AE & Stevens, DL. Streptococcus pyogenes. in *Mandell, Douglas, and Bennett's principles and practice of infectious diseases.* (eds. Bennett, J. E., Dolin, R. & Blaser, M. J.) 2285–2299 (Elsevier/Saunders, 2015).
19. Cillóniz, C. *et al.* Microbial aetiology of community-acquired pneumonia and its relation to severity. *Thorax* **66,** 340–346 (2011).
20. Castelblanco, R. L., Lee, M. & Hasbun, R. Epidemiology of bacterial meningitis in the USA from 1997 to 2010: a population-based observational study. *Lancet Infect. Dis.* **14,** 813–819 (2014).
21. Sobel, J. D. Vulvovaginal candidosis. *Lancet Lond. Engl.* **369,** 1961–1971 (2007).
22. Liu, F., Liao, Q. & Liu, Z. Mannose-binding lectin and vulvovaginal candidiasis. *Int. J. Gynaecol. Obstet. Off. Organ Int. Fed. Gynaecol. Obstet.* **92,** 43–47 (2006).

23. Nicolle, L. E. Uncomplicated urinary tract infection in adults including uncomplicated pyelonephritis. *Urol. Clin. North Am.* **35,** 1–12, v (2008).

24. Valiquette, L. Urinary tract infections in women. *Can. J. Urol.* **8 Suppl 1,** 6–12 (2001).

25. Paradise, J. L. *et al.* Efficacy of tonsillectomy for recurrent throat infection in severely affected children. Results of parallel randomized and nonrandomized clinical trials. *N. Engl. J. Med.* **310,** 674–683 (1984).

26. Schappert, S. M. Office visits for otitis media: United States, 1975-90. *Adv. Data* 1–19 (1992).

27. Heikkinen, T. & Chonmaitree, T. Importance of respiratory viruses in acute otitis media. *Clin. Microbiol. Rev.* **16,** 230–241 (2003).

28. Rosenfeld, R. M. Surgical prevention of otitis media. *Vaccine* **19 Suppl 1,** S134–139 (2000).

29. McDonald, S., Langton Hewer, C. D. & Nunez, D. A. Grommets (ventilation tubes) for recurrent acute otitis media in children. *Cochrane Database Syst. Rev.* CD004741 (2008). doi:10.1002/14651858.CD004741.pub2

30. Bulik-Sullivan, B. *et al.* An atlas of genetic correlations across human diseases and traits. *Nat. Genet.* **47,** 1236–1241 (2015).

31. Bulik-Sullivan, B. K. *et al.* LD Score regression distinguishes confounding from polygenicity in genome-wide association studies. *Nat. Genet.* **47,** 291–295 (2015).

32. Kvestad, E. *et al.* Recurrent otitis media and tonsillitis: common disease predisposition. *Int. J. Pediatr. Otorhinolaryngol.* **70,** 1561–1568 (2006).

33. Cesario, T. C. Viruses associated with pneumonia in adults. *Clin. Infect. Dis. Off. Publ. Infect. Dis. Soc. Am.* **55,** 107–113 (2012).

34. Wu, X. *et al.* Incidence of Respiratory Viral Infections Detected by PCR and Real-Time PCR in Adult Patients with Community-Acquired Pneumonia: A Meta-Analysis. *Respiration* **89,** 343–352 (2015).

35. Yang, J., Lee, S. H., Wray, N. R., Goddard, M. E. & Visscher, P. M. GCTA-GREML accounts for linkage disequilibrium when estimating genetic variance from genome-wide SNPs. *Proc. Natl. Acad. Sci. U. S. A.* **113,** E4579–4580 (2016).

36. Visscher, P. M. *et al.* Statistical power to detect genetic (co)variance of complex traits using SNP data in unrelated samples. *PLoS Genet.* **10,** e1004269 (2014).

37. Casselbrant, M. L. *et al.* The heritability of otitis media: a twin and triplet study. *JAMA* **282,** 2125–2130 (1999).

38. Kvestad, E. *et al.* Heritability of recurrent tonsillitis. *Arch. Otolaryngol. Head Neck Surg.* **131,** 383–387 (2005).

39. Carmelli, D., Colrain, I. M., Swan, G. E. & Bliwise, D. L. Genetic and environmental influences in sleep-disordered breathing in older male twins. *Sleep* **27,** 917–922 (2004).

40. Zuk, O., Hechter, E., Sunyaev, S. R. & Lander, E. S. The mystery of missing heritability: Genetic interactions create phantom heritability. *Proc. Natl. Acad. Sci. U. S. A.* **109,** 1193–1198 (2012).

41. Morris, A. P. *et al.* Large-scale association analysis provides insights into the genetic architecture and pathophysiology of type 2 diabetes. *Nat. Genet.* **44,** 981–990 (2012).
